# Solid-Phase Synthesis of Phosphorothioate/Phosphonothioate and Phosphoramidate/Phosphonamidate Oligonucleotides

**DOI:** 10.3390/molecules24101872

**Published:** 2019-05-15

**Authors:** Ondřej Kostov, Radek Liboska, Ondřej Páv, Pavel Novák, Ivan Rosenberg

**Affiliations:** Institute of Organic Chemistry and Biochemistry AS CR, v.v.i., Flemingovo nám. 2, 166 10 Prague 6, Czech Republic; liboska@uochb.cas.cz (R.L.); pavel.novak@uochb.cas.cz (P.N.)

**Keywords:** *H*-phosphinate, *H*-phosphonate, phosphoramidite, oligonucleotide, phosphonate, phosphorothioate, phosphonothioate, phosphoramidate, phosphonamidate

## Abstract

We have developed a robust solid-phase protocol which allowed the synthesis of chimeric oligonucleotides modified with phosphodiester and *O*-methylphosphonate linkages as well as their *P-S* and *P-N* variants. The novel *O*-methylphosphonate-derived modifications were obtained by oxidation, sulfurization, and amidation of the *O*-methyl-(*H*)-phosphinate internucleotide linkage introduced into the oligonucleotide chain by *H*-phosphonate chemistry using nucleoside-*O*-methyl-(*H*)-phosphinates as monomers. The *H*-phosphonate coupling followed by oxidation after each cycle enabled us to successfully combine *H*-phosphonate and phosphoramidite chemistries to synthesize diversely modified oligonucleotide strands.

## 1. Introduction

Recently, we published a detailed study on the influence of the incorporation of 2′-deoxy-nucleoside 3′-*O*- and 5′-*O*-methylphosphonate units on the hybridization properties of modified DNA strands and on their ability to elicit *E. coli* RNase H activity in heteroduplexes [1]. Although the insertion of the bridging –CH_2_– group into the phosphodiester linkage should increase the total entropy of the system due to an additional degree of freedom, leading to duplex destabilization, only the incorporation of 3′-*O*-methylphosphonate units into the DNA strand decreased the stability of the appropriate heteroduplexes. In contrast, the presence of 5′-*O*-methylphosphonate units slightly stabilized the ^modif^DNA*RNA heteroduplexes. These oligonucleotides, known as MethylPhosphonate Nucleic Acids (MePNA), when they contained various ratios of nucleoside-5′-phosphate and 5′-*O*-methyl-phosphonate units in an alternating mode exhibited superior enhancement of the RNase H cleavage rate.

To improve the synthesis of MePNA, we developed the straightforward synthesis of nucleoside-*O*-methyl-(*H*)-phosphinates and 5′-deoxynucleoside-5′-*S*-methyl-(*H*)-phosphinates [2,3] that were closely related to the well-known nucleoside *H*-phosphonates, [4,5] and demonstrated their compatibility with the *H*-phosphonate chemistry of the oligonucleotide synthesis. Recently, Herdewijn [6] has exploited this methodology to introduce various nucleoside phosphonates into oligonucleotides.

**Figure 1:**
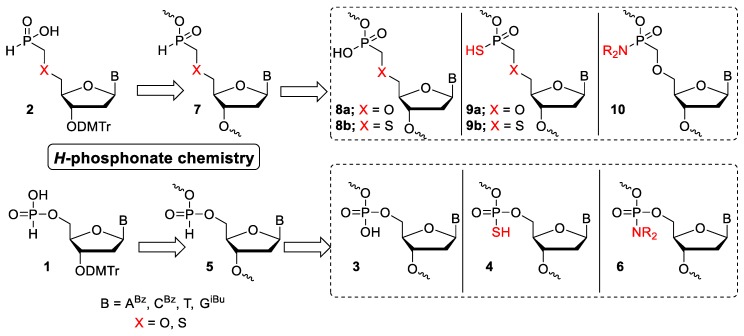
Overview of possible combinations of the phosphodiester **3**, **4**, **6** and phosphonate **8a**, **8b**, **9a**, **9b** and **10** linkages.

In this study, we present the extension of our methodology on the synthesis of modified oligonucleotides by a combination of phosphoramidite and *H*-phosphonate chemistries using nucleoside phosphoramidite, and *H*-phosphonate **1**, nucleoside-*O*-methyl-(*H*)-phosphinate, and 5′-deoxynucleoside-5′-*S*-methyl-(*H*)-phosphinate monomers **2** as building blocks (Figure 1), respectively. Standard phosphoramidite chemistry allows the synthesis of phosphate **3**, and phosphorothioate **4** internucleotide linkages. *H*-phosphonate chemistry affords even wider possibilities since the *H*-phosphonate internucleotide linkage in **5** can be oxidized, sulfurized, or amidated to form appropriate phosphate **3** [7,8], phosphorothioate **4** [9] or phosphoramidate **6** [10] internucleotide linkages (Figure 1). To be able to synthesize oligonucleotides modified with phosphodiester, *O*-methylphosphonate, and 5′-deoxy-5′-*S*-methylphosphonate linkages, we developed procedures which allowed the synthesis of *H*-phosphinate internucleotide linkage **7** (Figure 1). This linkage could be oxidized, sulfurized, or amidated to form the *O*-methylphosphonate **8a**, *O*-methylphosphonothioate **9a**, *O*-methylphosphonamidate **10**, *S*-methylphosphonate **8b** and *S*-methylphosphonothioate **9b** internucleotide linkages, respectively. The insertion of the oxidation step after each *H*-phosphonate coupling step [11,12] allowed us to combine easily phosphonate-derived units with the phosphodiester ones and to prepare unique set of modified oligonucleotides.

## 2. Results and Discussion

### 2.1. Study on Model Dimers

**Scheme 1:**
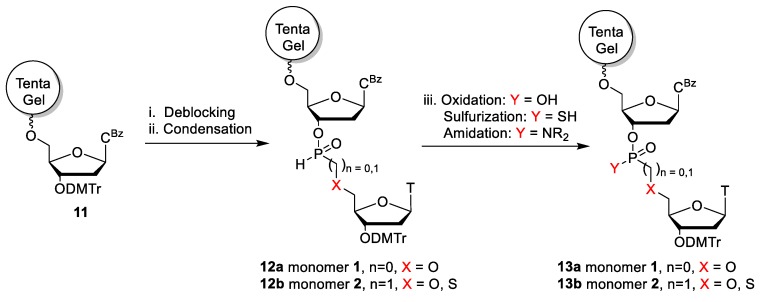
Model dimer for the optimization of *H*-phosphonate chemistry.

Our results on novel nucleoside-*O*-methyl-(*H*)-phosphinates [2,3] as monomers for *H*-phosphonate chemistry prompted us to develop robust synthetic protocols that would allow the synthesis of oligonucleotides bearing any combination of phosphodiester **3**, phosphorothioate **4**, phosphoramidate **6**, *O*-methylphosphonate **8a**, *O*-methylphosphonothioate **9a**, *O*-methyl-phosphonamidate **10**, *S*-methylphosphonate **8b** and *S*-methylphosphonothioate **9b** internucleotide linkages (Figure 1). The optimization of the oxidative couplings affording the abovementioned modified bonds was performed on a model dimer. Thus, TentaGel modified with 4-*N*-benzoyl-2′-deoxy-3′-*O*-DMTr-cytidine-5′-hemisuccinate **11** was extended with thymidine-*O*-methyl-(*H*)-phosphinate or thymidine-5′-deoxy-5′-*S*-methyl-(*H*)-phosphinate monomer **2** to provide TentaGel-attached (dCT) dimer with *H*-phosphinate internucleotide linkage **12b**. This linkage was subjected to oxidative procedures (oxidation/sulfurization/amidation) under various conditions (Scheme 1). All experiments with *H*-phosphinate monomers **2** were always compared to the standard thymidine *H*-phosphonate **1** as a control of the efficacy of the reaction conditions (For more details see Supporting Information).

#### 2.1.1. *H*-Phosphonate Coupling

The optimization of coupling reaction of *H*-phosphinate monomers **2** [2,3] was performed at 0.1 M concentration in acetonitrile-pyridine mixture (1:1) with a series of activating agents [13] commonly used in *H*-phosphonate chemistry such as pivaloyl chloride, adamantanecarbonyl chloride, 2-chloro-5,5-dimethyl-1,3,2-dioxaphosphorinane 2-oxide (DMOCP), diphenyl chloro-phosphate (DPCP), and bis(2-oxo-3-oxazolidinyl)phosphinic chloride (OXP) at 0.3 M concentration in acetonitrile-pyridine mixtures (95:5). Of all tested coupling agents, we selected DMOCP as the activator of choice for the condensation of both *H*-phosphonate **1** and *H*-phosphinates **2**. The only difference was the duration of the coupling step. The *H*-phosphinate monomers required 10 min condensation time compared to 5 min *H*-phosphonate condensation.

#### 2.1.2. Oxidation/Amidation/Sulfurization of *H*-Phosphinate Internucleotide Linkage

As we published previously [2,3], the use of water-containing oxidation mixtures such as 0.1 M iodine in pyridine-water mixture (98:2) reported for the oxidation of *H*-phosphonate linkage **12a** [7,8] could not be used for the oxidation of *H*-phosphinate bond **12b** due to its lability under aqueous conditions and very fast hydrolytic cleavage. The use of 0.1 M iodine in pyridine-methanol (50:50) mixture led to only 80% yield of the expected *MeO-P^(V)^* product. However, Atherton-Todd reaction [14,15,16] conditions (CCl_4_-pyridine-methanol) provided an acceptable 95% yield of *MeO-P^(V)^* product. Moreover, no hydrolysis of internucleotide linkage was observed.

Our further research focused on the composition of the CCl_4_-based oxidation mixture, especially with respect to the base used and its basicity/nucleophilicity. The replacement of pyridine with 1-methylimidazole or 1-methylimidazole-triethylamine mixture, led to a quantitative oxidation of both *H*-phosphonate and *H*-phosphinate linkages. We found that methanol and 3-hydroxypropionitrile exhibited similar reactivity to provide methyl and 2-cyanoethyl esters, respectively, however, the 2-cyanoethyl ester represented more stable ester group. Since 3-hydroxypropionitrile is immiscible with tetrachloromethane, DCM was used as a co-solvent in oxidation mixture (Table 1).

Encouraged by these results and based on the fact that the amines were commonly used nucleophiles in Atherton-Todd reaction [15,16], we examined the preparation of electroneutral oligonucleotides with non-bridging *P–N* bond using oxidative amidation of the *H*-phosphinate linkage. In contrast to the reported phosphoramidate oligonucleotides [14,17,18], the phosphonamidate oligonucleotides have not been published so far. Two representative amines, the primary *N*,*N*-dimethylethylenediamine which was already incorporated into phosphoramidate oligonucleotides [17,18] and morpholine [19] as a representative of secondary amines were selected as amidation reagent. We examined various ratios of tetrachloromethane and the amines in the mixture, and the time of the amidation reaction. A quantitative amidation of both *H*-phosphonate and *H*-phosphinate linkages was achieved after 180 min using a mixture of CCl_4_/amine/DCM in 3:2:5 ratio (DCM was used to keep forming amine hydrochloride in the solution).

The sulfurization of *H*-phosphinate **12b** linkage to afford phosphonothioate bond was achieved using two methods. The first method employed elemental sulfur in various solvents and in the presence of base [9], and provided directly expected charged dimers. The reaction performed with 0.5 M solution of elemental sulfur in pyridine provided the desired products quantitatively within 20 min. Since this type of sulfurization could be only used as the last step of the synthetic cycles, we also focused on the second method employing sulfur-transfer reagent (*N*-[(2-cyanoethyl)-sulfanyl]succinimide, CSS) [20,21]. This method was applicable either in the last *H*-phosphonate cycle or after each synthetic cycle affording *S*-(2-cyanoethyl) esters which were then cleaved by β-elimination to provide phosphonothioate linkages. Thus the sulfurization performed with 0.2 M CSS in a mixture of acetonitrile and silylating agent BSTFA [20] (29:1) provided the *S*-(2-cyanoethyl)-protected dimers quantitatively within 30 min. β-Elimination reaction was performed at the end of the synthesis with 1 M DBU in acetonitrile for 3 min. The optimal reaction conditions of the individual oxidation steps are summarized in Table 1.

#### 2.1.3. Stability of Modified Internucleotide Linkages

During the optimization of the condensation and oxidation steps, we observed different behavior and reactivity of both *H*-phosphonate and *H*-phosphinate internucleotide linkages. Therefore we decided to examine the stability of these linkages using a series of modified d(CT) dimers. All experiments were performed on LCMS at several time intervals.

The stability of phosphorothioate and phosphonothioate linkages was examined in the presence of two potential desulfurizing agents—sodium periodate (0.05 M aqueous solution) and iodine (0.05 M solution in pyridine-water mixture (98:2)).

It was reported that the periodate anion partially desulfurized the phosphorothioate TpT dimer [22]. In agreement with this, the treatment of phosphorothioate d(CT) dimer **14a** with sodium periodate quantitatively afforded the product of desulfurization, d(CT) phosphate dimer **15a**, after 12 h. In case of phosphonothioate dimer **14b**, complete cleavage of the internucleotide linkage to phosphonate **16b** was observed. On the other hand, *S*-methylthiophosphonate dimer **14c** was cleaved to phosphonate **16c** only partially (53%). The rest of the dimer underwent desulfurization and oxidation of *S*-methylphosphonate linkage to *S(=O)*-methylphosphonate **15d** (47%).

**Scheme 2:**
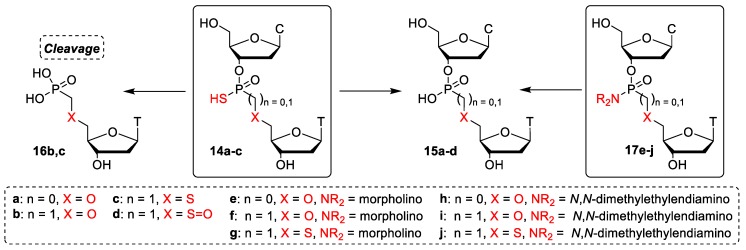
Stability of phosphorothioate/phosphonothioate and phosphoramidate/phosphonamidate internucleotide linkages.

In case of iodine-promoted desulfurization, all tested dimers **14a**–**c** were quantitatively and cleanly transformed to phosphate/phosphonate dimers **15a**–**c** within 5 min of treatment. Neither cleavage nor oxidation of *S*-methylphosphonate in the case of dimer **14c** was observed (Scheme 2).

We also tested the stability of *P–N* bonds of dimers **17e**–**h** under acidic conditions (pH = 1 for 5 h; Scheme 2). Comparing the stability of phosphoramidate and phosphonamidate linkages, phosphoromorpholidate dimer **17e** exhibited higher stability (35% hydrolysis to dimer **15a**) than phosphonomorpholidate dimer **17f** (78% hydrolysis to dimer **15b**). Suprisingly, *S*-methyl-phosphonomorpholidate dimer **17g** showed the highest stability, with only 18% hydrolysis to dimer **15c**.

Moreover, phosphonamidate linkages derived from primary amines were much more stable than the linkages derived from secondary amines (Scheme 2). Dimethylethylendiamino-phosphonamidate dimer **17i** and dimethylethylendiamino-*S*-methylphosphonamidate dimer **17j** exhibited similar stability, with only 6% hydrolysis to the dimers **15b** and **15c**, respectively. Dimethylethylendiamino-phosphoramidate dimer **17h** was hydrolyzed to the same extent as its morpholino derivative **17e** (36% hydrolysis to dimer **15a**).

### 2.2. Synthetic Protocols for Oligonucleotide Synthesis

Based on the results obtained on the level of dimers, we have developed three synthetic protocols (Synthetic protocols A, B, and C), and tested their efficacy on model homo-oligothymidylates **dT_10_1**–**dT_10_7** modified with various internucleotide linkages (Table 2).

#### 2.2.1. Synthetic Protocol A

**Scheme 3:**
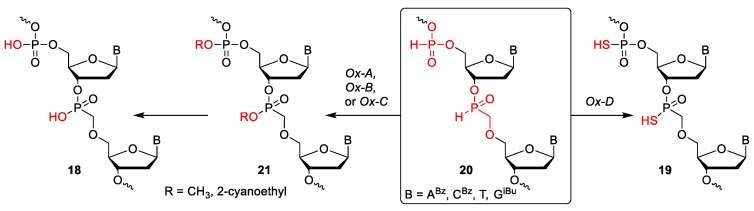
Synthetic protocol A—synthesis of fully modified strands.

This protocol was used for the synthesis of oligonucleotides fully modified with either phosphonate/phosphate **18** or thiophosphonate/thiophosphate **19** linkages (Scheme 3, Table 2). The nucleoside *H*-phosphonates **1** and *O*-methyl-(*H*)-phosphinates **2** were coupled on solid support using *H*-phosphonate chemistry, to provide oligonucleotide **20**. Since the use of *H*-phosphonate capping [23] led, in our hands, to the formation of substantial amount of side products, this step was omitted. After last synthetic cycle, all internucleotide linkages were either oxidized or sulfurized to afford oligonucleotides **21** or **19**, respectively (Scheme 3). The use of non-aqueous oxidation mixtures (Table 1; *Ox-A*, *Ox-B*, and *Ox-C*) that oxidized *P–H* bonds to *P–OR* bond (R = CH_3_, 2-cyanoethyl) led to complete oxidation. The methyl and 2-cyanoethyl ester groups were easily removed using thiophenol/TEA/DMF and gaseous ammonia, respectively, to afford the final product **18**.

The sulfurization of the *H*-phosphonate/*H*-phosphinate linkages proceeded smoothly using a solution of elemental sulfur in pyridine (Table 1; *Ox-D*). It is important to note that the elimination of traces of water from the oxidation and sulfurization mixtures was of the utmost importance. Therefore, all the oxidation mixtures were dried over 3 Å molecular sieves for 16 h prior the use. This protocol (For more details see Materials and Methods, Supporting Information) was successfully applied for the preparation of oligonucleotides **dT_10_1**, **dT_10_2**, **ON-8**, and **ON-9** (Table 2 and Table 3).

#### 2.2.2. Synthetic Protocol B

**Scheme 4:**
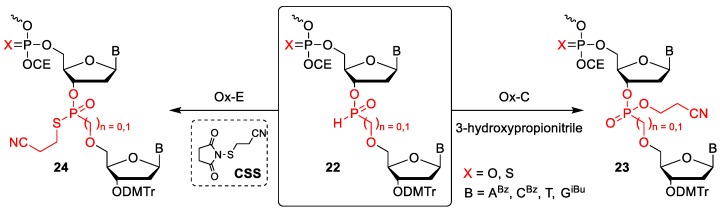
Synthetic protocol B—synthesis of oligonucleotides modified at specific positions.

This protocol combined phosphoramidite and *H*-phosphonate chemistries to introduce the phosphodiester/phosphorothioate and *O*-methylphosphonate/*O*-methylphosphonothioate inter-nucleotide linkages, respectively, at specific positions of the oligonucleotide strand (Scheme 4). The *P^III^* triester bonds were oxidized with 1M *t*-BuOOH in DCM or sulfurized using Sulfurizing Reagent II (DDTT, Glen Research, Sterling, VA, USA) after each phosphoramidite coupling step. The oxidation of the *H*-phosphinate internucleotide linkages in oligonucleotide **22** was performed after each step with the mixture *Ox-C* utilizing 3-hydroxypropionitrile (Table 1) to afford product **23**. The use of the oxidation mixtures *Ox-A* and *Ox-B* containing methanol did not afford the desired oligonucleotide. The explanation for this behaviour might lie in the facts that neutral methyl esters of phosphonates could serve as powerful methylating agents in the presence of a nucleophile such as sulfur compound or pyridine, and that the cleavage of the methyl protecting group of the phosphonate moiety gave rise to a *P–OH* reactive center that interfered with the coupling steps leading to a complex mixture of products.

The synthesis of *S*-protected phosphonothioate internucleotide linkages in oligonucleotide **24** was achieved using the sulfur-transfer reagent (CSS) [20,21] (Table 1; *Ox-E*) to form *S*-(2-cyanoethyl) ester of the phosphonothioate linkage. After the synthesis of the entire oligonucleotide, the *S*-(2-cyanoethyl) ester groups were removed by β-elimination using 1 M DBU in acetonitrile. Surprisingly, gaseous ammonia could not be used for the β-elimination reaction since it caused cleavage of protected phosphonothioate internucleotide linkages to afford the degradation of the oligonucleotide strand.

The introduction of oxidation or sulfurization after each coupling step allowed the use of standard acetic anhydride-based capping procedure. The protocol (For more details see Materials and Methods, Supporting Information) was successfully applied for the preparation of oligonucleotides **dT_10_1**, **dT_10_2**, **dT_10_3**, **dT_10_4**, **ON-4**, **ON-5**, **ON-6**, **ON-7**, **ON-10**, and **ON-11** (Table 2 and Table 3).

#### 2.2.3. Synthetic Protocol C

**Scheme 5:**
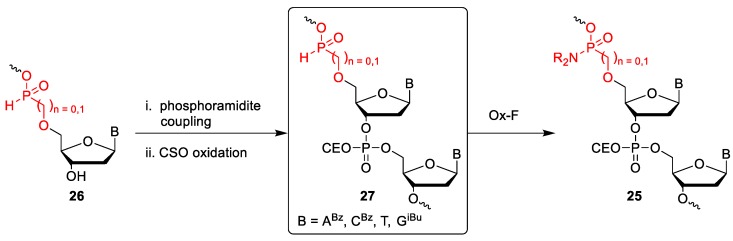
Synthetic protocol C—synthesis of phosphoramidate/phosphonamidate strands.

This protocol combined phosphoramidite and *H*-phosphonate chemistries, and allowed the introduction of phosphoramidate and phosphonamidate linkages at specific sites of the oligonucleotide **25** (Scheme 5). We found that the *H*-phosphonate and *O*-methyl-(*H*)-phosphinate internucleotide linkages in oligonucleotide **26** were neither cleaved nor oxidized by oxaziridine CSO [24,25,26] which represented a mild phosphoramidite oxidation agent. This reagent allowed selective oxidation of *P^III^* linkage in the presence of *H*-phosphonate or *H*-phosphinate bonds. The amidation of the *P–H* bond in **27** has to be performed at the end of the oligonucleotide synthesis. This synthetic arrangement did not allow the use of acetic anhydride-based capping due to the instability of *H*-phosphonate and *H*-phosphinate linkages in **27**. The use of UniCap [27] could be a solution, however, in our hands we did not observe any improvement.

The amidation of *P–H* bonds with morpholine or *N*,*N*-dimethylenediamine in **27** (Scheme 5) was performed using CCl_4_/amine/DCM mixture (Table 1; *Ox-F*). Such mixed sequences containing *P-morpholine* and *P–OR* linkages cannot be prepared using protocol B because each detritylation step was accompanied with a partial hydrolysis of the *P-morpholine* bond due to the residual water content in detritylation mixture. Recently, Damha and Vlaho [28] reported the preparation of a series phosphoramidate oligonucleotides via oxidative amidation using primary amines (*N*,*N*-dimethyl-ethylendiamine, 3-(dimethylamino)propylamine, 4-dimethylaminobutylamine, isopentylamine) which were completely resistant to detritylation conditions.

This protocol (For more details see Materials and Methods, Supporting Information) was successfully applied for preparation of oligonucleotides **dT_10_3**, **dT_10_4**, **dT_10_5**, **dT_10_6**, **dT_10_7**, **ON-12**, **ON-13**, **ON-14**, and **ON-15** (Table 2 and Table 3).

#### 2.2.4. Synthesis of Hetero-Oligonucleotides

To verify general usefulness of the above mention protocols, we also prepared a set of hetero-oligonucleotides modified at specific positions with phosphodiester/phosphonate, phosphorothioate/phosphonothioate and phosphoramidate/phosphonamidate linkages (Table 3). These oligonucleotides were prepared in excellent yields and purity. Therefore, the developed protocols represent novel and effective approaches to the synthesis of variety of phosphonate oligonucleotides modified with diverse types of linkages (For more details, and hybridization properties of **ON-1**–**ON-15** see Supporting Information).

## 3. Materials and Methods

### 3.1 General Information

The 5′→3′ trityl-off synthesis of oligonucleotide was performed using phosphoramidite and *H*-phosphonate method on GeneSyn and MOS synthesizers (IOCB Prague, Prague, Czech Republic) using 3′-*O*-dimethoxytritylnucleoside-5′-hemisuccinate-modified LCAA-CPG and TentaGel solid supports in a 0.5 µmol scale. For the phosphoramidite chemistry all monomers, and oxidation and sulfurization agents were used according to the supplier’s recommendations (Glen Research). Condensation of *H*-phosphinate monomers were performed according the synthetic protocols in Table 4, Table 5, and Table 6 (for more details see Supporting Information).

### 3.2. H-Phosphonate Chemistry

Pyridine-acetonitrile (1:1) solutions of 0.1 M monomers (110 µL) and 0.3 M DMOCP (110 µL) were used for each coupling step (10 min). The oxidation of the oligonucleotide chain was achieved using conditions in Table 2. In case of methyl esters, the support was dried in vacuo, treated with freshly prepared thiophenol-Et_3_N-DMF mixture (23:32:45; *v*/*v*) for 4 h, rinsed with dry acetonitrile, dried under vacuum, and treated with gaseous NH_3_ (0.7 MPa) at rt overnight. In case of 2-cyanoethyl esters was thiophenol treatment omitted. The deprotected and released oligonucleotide was eluted from CPG with 0.1 M-TEAB in acetonitrile/water (1 mL; 1:1, *v*/*v*) and analyzed on a DNAPac PA100 column (Thermo Fisher Scientific, Waltham, MA, USA, 4 × 250 mm) at a flow rate of 1 mL/min using different gradients. The purification of oligonucleotide was performed on a DNAPac PA100 column (Thermo Fisher Scientific), 9 × 250 mm at a flow rate of 3 mL/min,). Pure oligonucleotide was desalted on a Luna C18 column (Phenomenex, Torrance, CA, USA, 5 μm; 10 × 50 mm;) at a flow rate of 5 mL/min using a linear gradient of acetonitrile (0→50%, 20 min) in 0.1 M aq. triethylammonium hydrogencarbonate buffer (pH 8); effluent containing oligonucleotide was evaporated to dryness on a CentriVap apparatus (Labconco, Kansas City, MO, USA) and co-evaporated with MeOH (3 × 1 mL).

## 4. Conclusions

In this study, we present the use of protected nucleoside-*O*-methyl-(*H*)-phosphinates as monomers for oligonucleotide synthesis using *H*-phosphonate chemistry. The formed *O*-methyl-(*H*)-phosphinate internucleotide linkages could be oxidized, sulfurized, or amidated similarly to the *H*-phosphonate bonds. We developed appropriate oxidation mixtures and three synthetic protocols allowing the combination of *H*-phosphonate and phosphoramidite chemistries to incorporate individual modifications either into the entire chain or at specific positions of the modified oligonucleotide. A series of DNA oligonucleotides modified with a combination of the phosphonate, phosphonothioate, phosphonamidate, phosphorothioate, phosphoramidate, and phosphodiester internucleotide linkages have been successfully synthesized to verify the robustness of the presented synthetic protocols. To our best knowledge, this is the first report of the synthesis of oligonucleotides bearing phosphonamidate and phosphonothioate internucleotide linkages. The developed protocols allow the synthesis of variety of phosphonate oligonucleotides that might be used as tools in biochemistry and biology, and in research and development of oligonucleotide-based drugs.

## Figures and Tables

**Table 1:** The conditions of oxidation, sulfurization and amidation.

Code	Mixture	Oxidation Time (min)	
		*H*-Phosphonate	*H*-Phosphinate
*Ox-A*	CCl_4_/MeOH/MeIm (7:2:1)	15	30
*Ox-B*	CCl_4_/MeOH/MeIm/Et_3_N (7:2:0.5:0.5)	10	20
*Ox-C*	0.1 M HOCH_2_CH_2_CN in CCl_4_/DCM/MeIm (6:2:2)	15	30
*Ox-D*	0.5 M sulfur in pyridine	20	20
*Ox-E*	0.2 M CSS in ACN/BSTFA (29:1)	30	30
*Ox-F*	CCl_4_/amine */DCM (3:2:5)	180	180

* Morpholine or *N*,*N*-dimethylethylendiamine.

**Table 2:** Summary of model homo-oligothymidylates **dT_10_1**–**dT_10_7**.

Internucleotide Linkages	Code	Sequence 5′→3′	Synthetic Protocol (HPLC Purity)	MALDI TOF Calcd Found	
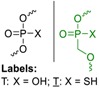	**dT_10_1**	**d(T T T T T T T T T T)**	**A** (70%), **B** (74%), **C** (64%)	3020.5516	3020.5733
	**dT_10_2**	**d(TTTTTTTTTT)**	**A** (59%), **B** (77%)	3164.3460	3164.3219
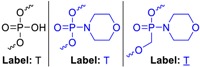	**dT_10_3**	**d(T T T T T T T T T T)**	**B** (36%), **C** (88%)	3187.3049	3187.1703
	**dT_10_4**	**d(T T T T T T T T T T)**	**B** (42%), **C** (70%)	3229.3859	3229.1063
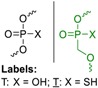	**dT_10_5**	**d(T T T T T T T T T T)**	**C** (30%)	3070.2479	3070.7329
	**dT_10_6**	**d(T T T T T T T T T T)**	**C** (75%)	2979.9839	2980.3970
	**dT_10_7**	**d(T T T T T T T T T T)**	**C** (77%)	3028.1696	3028.4048

**Table 3:** Summary of hetero-oligonucleotides **ON-1**–**ON-15**.

Internucleotide Linkages	Code	Sequence 5′→3′	Synthetic Protocol (HPLC Purity)	MALDI TOF	
				Calcd	Found
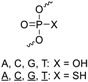	**ON-1**	**d(G C A T A T C A C)**	**B** (86%)	2729.4322	2729.5331
	**ON-2**	**d(G C A T A TC A C)**	**B** (84%)	2761.3865	2761.1589
	**ON-3**	**d(G CATATCAC)**	**B** (81%)	2809.3180	2809.3292
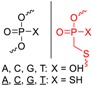	**ON-4**	**d(G C A T A T C A C)**	**B** (21%)	2771.4792	2771.3983
	**ON-5**	**d(G CATATCAC)**	**B** (56%)	2899.2964	2899.2376
	**ON-6**	**d(G C A T A T C A C)**	**B** (51%)	2819.4106	2819.2887
	**ON-7**	**d(G CATA TCA C)**	**B** (43%)	2851.3650	2851.3559
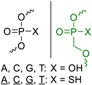	**ON-8**	**d(G C A T A T C A C)**	**A** (76%)	2723.5477	2723.7076
	**ON-9**	**d(G CATATCAC)**	**A** (84%)	2851.3650	2851.2513
	**ON-10**	**d(G C A T A T C A C)**	**B** (86%)	2771.4791	2771.3636
	**ON-11**	**d(G CATA TCA C)**	**B** (86%)	2803.4335	2803.5659
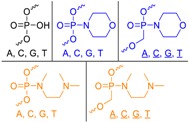	**ON-12**	**d(G C A T A T C A C)**	**C** (59%)	2930.7213	2930.6797
	**ON-13**	**d(G C A T A T C A C)**	**C** (66%)	2975.8631	2975.7741
	**ON-14**	**d(G C A T A T C A C)**	**C** (78%)	2888.6743	2888.8365
	**ON-15**	**d(G C A T A T C A C)**	**C** (79%)	2933.8162	2933.7591

**Table 4:** Synthetic Protocol A (For more details see Supporting Information).

Procedure	Reagents	Volume/Time
*H*-Phosphonate/*H*-Phosphinate Condensation Step		
Deblocking	3% DCA in DCM	Flow through the column 6 mL/2 min
Condensation	0.1M monomer in ACN/Py (1:1)	120 µL/10min
	0.3M DMOCP in ACN/Py (95:5)	
Oxidation	*Ox-A*, *Ox-B*, *Ox-D*	220 µL/30 min
Methyl-ester cleavage *	PhSH/Et_3_N/DMF(1:1.4:2)	300 µL/4 h
Cleavage from solid support	Gaseous ammonia at 0.7 MPa	Gas chamber/16 h

* Applicable only in case of Ox-A and Ox-B mixtures containing methanol.

**Table 5:** Synthetic Protocol B (For more details see Supporting Information).

Procedure	Reagents	Volume/Time
*H*-Phosphonate/*H*-Phosphinate Condensation Step		
Deblocking	3% DCA in DCM	Flow through the column 6 mL/2 min
DCA washout	5% pyridine in methanol	
Condensation	0.1M monomer in ACN/Py (1:1)	120 µL/10 min
	0.3M DMOCP in ACN/Py (95:5)	
Oxidation or Sulfurization	*Ox-C* or *Ox-E*	220 µL/30 min
Capping	Ac_2_O/Py/THF (1:1:1:17)	2 × 150 µL/3 min
	MeIm/THF (1:9)	
Phosphoramidite Condensation Step		
Deblocking	3% DCA in DCM	Flow through the column 6 mL/2 min
DCA washout	5% pyridine in methanol	
Condensation	0.1M monomer in ACN	120 µL/5 min
	0.25M ETT in ACN	
Oxidation or Sulfurization	CSO, tBuOOH or DDTT	220 µL/3.5 min
End of Synthetic Cycle		
S-cyanoethyl-ester cleavage *	1M DBU in ACN	220 µL/3 min
Cleavage from solid support	Ammonia gas at 0.7 MPa	Gas chamber/16 h

* Applicable only in case of CSS sulfurization mixtures.

**Table 6:** Synthetic Protocol C (For more details see Supporting Information).

Procedure	Reagents	Volume/Time
*H*-Phosphonate/*H*-Phosphinate Condensation Steps		
Deblocking	3% DCA in DCM	Flow through the column 6 mL/2 min
Condensation	0.1 M monomer in ACN/Py (1:1)	120 µL/10 min
	0.3 M DMOCP in ACN/Py (95:5)	
Phosphoramidite Condensation Step		
Deblocking	3% DCA in DCM	Flow through the column 6 mL/2 min
DCA washout	5% pyridine in methanol	
Condensation	0.1 M monomer in ACN	120 µL/5 min
	0.25 M ETT in ACN	
Oxidation	CSO	220 µL/3.5 min
End of Synthetic Cycle		
Amidation of *P*–*H* bond	*Ox-F*	220 µL/180 min
Cleavage from solid support	Ammonia gas at 0.7 MPa	Gas chamber/16 h

## Supplementary Materials

The following are available online. The supplementary materials containing all experimental details.
