# Supplementary material for: Solid-Phase Synthesis of Phosphorothioate/Phosphonothioate and Phosphoramidate/Phosphonamidate Oligonucleotides
Source: Molecules. 2019 May 15;24(10):1872. doi: 10.3390/molecules24101872 (PMC6571627; doi:10.3390/molecules24101872)
Supplement: Supplementary file 1 [file molecules-24-01872-s001.pdf]

SOLID-PHASE SYNTHESIS OF PHOSPHOROTHIOATE/PHOSPHONOTHIOATE AND PHOSPHORAMIDATE/PHOSPHONAMIDATE OLIGONUCLEOTIDES

1. Optimizing conditions for synthesis of modified oligonucleotides 1
   1.1 Starting conditions 1
      1.1.1 Monomers 1
      1.1.2 Oligonucleotide synthesizer 1
      1.1.3 Deblocking step 2
      1.1.4 Solid support 3
   1.2 Study on model dimers 4
      1.2.1 Coupling optimization 4
      1.2.2 Oxidation 6
      1.2.3 Amidation 10
      1.2.4 Sulfurization 13
      1.2.5 Summary of oxidation procedures 15
   1.3 Stability of modified internucleotide linkages 16
      1.3.1 Stability of non-bridging P-SH bond 16
      1.3.2 Stability of non-bridging P-N bond 20
      1.3.3 Stability of H-phosphonate/phosphinate ester linkage 23
   1.4 Preparation of modified oligonucleotides 26
      1.4.1 Synthetic Protocol A 26
      1.4.2 Synthetic Protocol B 28
      1.4.3 Synthetic Protocol C 33
2. Physicochemical properties of ONs 37
3. Characterization of oligonucleotides 44
4. REFERENCES 57
5. APPENDIX 59

1. OPTIMIZING CONDITIONS FOR SYNTHESIS OF MODIFIED OLIGONUCLEOTIDES

1.1 Starting conditions

1.1.1 Monomers

We have recently published [1, 2] the preparation of 4-toluenesulfonyloxymethyl-(*H*)-phosphinic acid (**1**) that can be reacted with 2'-deoxy-3´-*O*-dimethoxytrityl-nucleosides **2** to form 2'-deoxyribonucleoside-*O*-methyl-(*H*)-phosphinate monomers **3**. In addition, the (*H*)-phosphinate **1** can be reacted with thiourea to form (*H*)-phosphinomethylisothiouronium tosylate **4**, which can be further reacted with 2'-deoxy-3'-*O*-dimethoxytrityl-5'-*O*-tosylnucleosides **5** to form the 5'-deoxy-5'-*S*-methyl-(*H*)-phosphinate monomers **6** (Scheme 1).

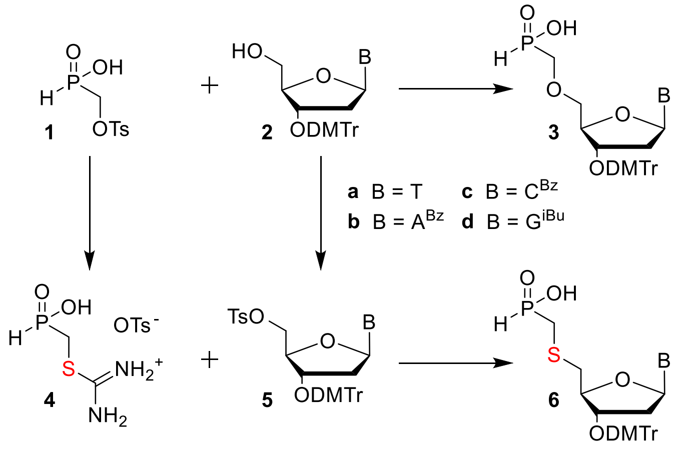

Scheme 1

1.1.2 Oligonucleotide synthesizer

To combine phosphoramidite and phosphotriester and/or *H*-phosphonate chemistries we built, in collaboration with the Institute of mechanic and electronic workshops, two special synthesizers where we can change independently all parameters of the individual synthetic procedures and create any other procedures

required. Usually two chemically different methods of oligonucleotide synthesis have been combined in dependence on monomers types.

1.1.3 Deblocking step

The detritylation step (deblocking) brought complication resulting in a low yield of the coupling step. The solution of 3% dichloroacetic acid **7** in DCM used for detritylation contained, depending on the content of water, variable amount of dichloroacetic anhydride **8**. Its presence partially acylated hydroxyl groups of deprotected oligonucleotide **9** resulting in dichloroacetate-capped oligonucleotide **10** (Scheme 2) [3]. The anhydride **8** is in equilibrium with **7** in dry DCM solution, so that the use of deblocking solution with 150-200 ppm of water (Karl-Fisher) prevented acylation of hydroxyl groups.

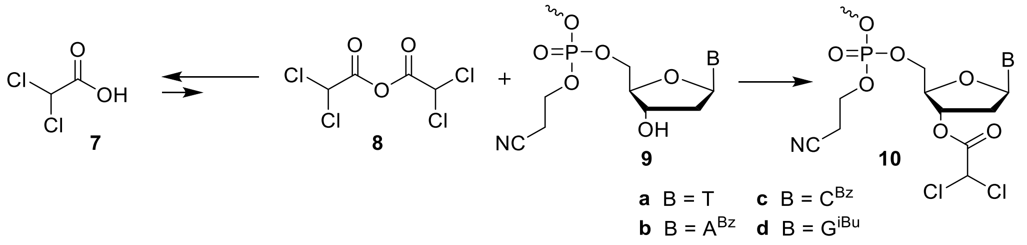

Scheme 2. Acylation during deblocking step.

Anhydride free deblocking mixture increased condensation yield of the next condensation step in case of phosphoramidite method, but showed another complication, based on the non-covalent binding of dichloroacetic acid **7** to the oligonucleotide backbone [4]. This complication concerns namely advanced phosphotriester and *H*-phosphonate chemistries, because the monomer is activated *via* mixed-anhydride with TIPSCl and DMOCP, respectively. These reagents may activate dichloroacetic acid, non-covalently bond to oligonucleotide chain so that partially dichloroacetate-capped oligonucleotide chain was formed. To overcome these problems, the increase of washing procedures was recommended after deblocking step [5] (*e.g.*, the use of washing solvents with defined amount of water [6], or use other solvent mixture such as pyridine-methanol).

To monitor the coupling step during the synthesis we measured electric conductivity of the effluent from the reactor *via* flow cell during the deblocking of the dimethoxytrityl groups. The effluent contained dimethoxytritylium dichloroacetate ion pair which is conductive in DCM. The obtained peak was integrated, and the area compared to the previous step to check step-by-step yields.

1.1.4 Solid support

Before optimizing synthetic protocols for new monomers, we focused on comparison of two most commonly used solid supports, the LCAA-CPG (Long Chain Alkylamine Controlled Pore Glass) and TentaGel S NH_2 (polystyrene-based resin grafted with polyethylene glycol with amino functional groups), where in both cases the nucleoside was attached to the free amino group through the hemisuccinate (Fig. 1). In contrast to CPG, TentaGel showed considerable particle size changes (swelling) in different solvents.

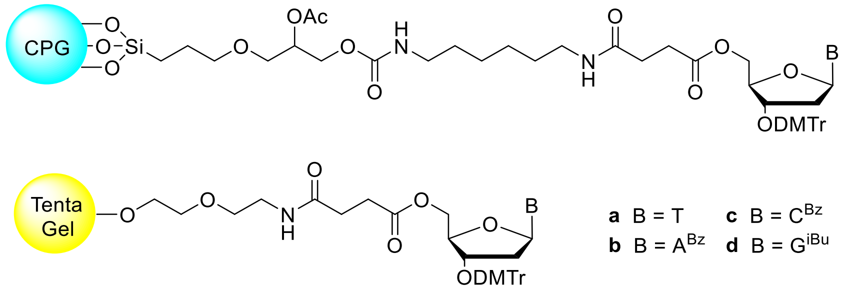

Figure 1. Solid supports tested for dimethoxytrityl response.

However, this fact was not a problem in the case of our synthesizer. We worked with 20 mg of solid support for each synthesis in a column with internal volume of 400 μl (20 mg of TentaGel represents app. 150-200 μl). Much more interesting was that the dimethoxytrityl response indicated several first coupling yields ranking 180-200% for the CPG. In the case of TentaGel, the dimethoxytrityl response showed yields in the range of 99-100%. The identical profile of products obtained from both solid supports suggested nonspecific interactions of the CPG with activated monomers which are washed out from CPG during deblocking step. For

the optimization of condensation steps we preferred, therefore, the use of TentaGel over LCAA-CPG.

1.2 Study on model dimers

1.2.1 Coupling optimization

The first coupling experiments with nucleoside-*O*-methyl-(*H*)-phosphinates **3**, and 5´-deoxynucleoside-5´-*S*-methyl-(*H*)-phosphinates **6,** we performed under conditions described for standard nucleoside-*H*-phosphonate. The use of pivaloyl chloride as the former coupling agent showed that unlike *H*-phosphonate, which condensed around 98-100%, monomers **3**, and **6** condensed in the range of 0-40%. This fact indicated a different reactivity of *H*-phosphonate and *H*-phosphinate groups.

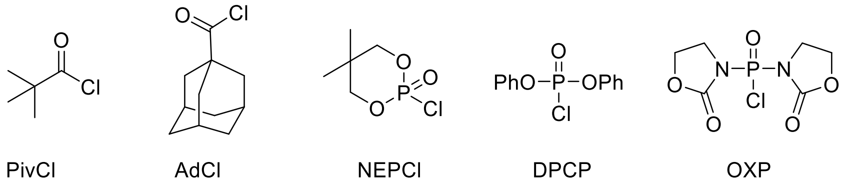

Figure 2. Examples of the most common condensing agents in *H*-phosphonate chemistry.

Therefore, we tested a series of activating agents [7] commonly used in *H*-phosphonate chemistry (Fig. 2), several solvents and their mixtures, bases, and concentration of reagents. In this case, we have tested monomers **3**, and **6** as compared to standard *H*-phosphonates. All condensing agents were tested in different solvent systems and bases (Tab. 1.) at two concentrations of monomers (0.1M monomer and 0.3M coupling agent, and 0.05M monomer and 0.15M coupling agent). The coupling time was 5, 15 and 30 min at rt. The replacement of acetonitrile (ACN) with tetrahydrofuran (THF) or dichloromethane (DCM) significantly reduced the efficiency of the condensation step for the standard *H*-phosphonate (as well as for monomers **3**, and **6**). The only alternative to ACN is nitromethane. There is also possibility to avoid the second solvent and use pyridine as the only solvent.

Table 1. Reaction conditions tested in the condensation step with different condensing agents.

	Used solvent mixtures and bases							
Monomer (c = 0.1M, or 0.05M)	Pyridine	ACN / Pyridine 1:1	DCM / Pyridine 1:1	THF / Pyridine 1:1	CH_3NO_2 / Pyridine 1:1	ACN / 2,6-Lutidine 1:1	ACN / MeIm 95:5	ACN / Et_3N 95:5
Condensation agent (c = 0.3M, or 0.15M)	Pyridine	ACN / Pyridine 95:5	DCM / Pyridine 95:5	THF / Pyridine 95:5	CH_3NO_2 / Pyridine 95:5	ACN / 2,6-Lutidine 95:5	ACN / MeIm 95:5	ACN / Et_3N 95:5
	*Yields (%) calculated from dimethoxytrityl conductivity response for H-phosphonate monomer and average value of monomers (**3**, **6**)*							
PivCl or AdCl	100<br>(~5)	100<br>(~10)	75<br>(~3)	88<br>(~6)	100<br>(~9)	70<br>(~4)	83<br>(~3)	48<br>(~8)
DMOCP	100<br>(~94)	100<br>(~100)	72<br>(~39)	93<br>(~56)	100<br>(~99)	66<br>(~28)	87<br>(~67)	45<br>(~32)
DPCP	95<br>(~84)	100<br>(~90)	58<br>(~29)	87<br>(~51)	100<br>(~89)	53<br>(~10)	84<br>(~66)	39<br>(~26)
OXP	88<br>(~18)	100<br>(~20)	39<br>(~9)	89<br>(~6)	77<br>(~17)	66<br>(~8)	70<br>(~5)	28<br>(~6)

We have also found that replacing pyridine in an acetonitrile mixture with triethylamine (Et_3N) or *N*-methylimidazole (MeIm) dramatically reduces the efficiency of condensation of *H*-phosphonate and *H*-phosphinate monomers. The use of 2,6-lutidine in place of pyridine reduced the reaction rate of the condensation step, probably due to a steric hindrance. While the standard *H*-phosphonate was acceptably activated with all condensing agents (Fig. 2) in the range of 80-100%, the individual *H*-phosphinate monomers provided very different results. None of the monomers **3**, and **6** exhibited condensation capability higher than 10% with pivaloyl chloride (PivCl) and 1-adamantanecarbonyl chloride (AdCl), and very weak condensation capability (10-20%) with bis(2-oxo-3-oxazolidinyl)phosphinic chloride (OXP). The best coupling yields for monomers **3**, and **6** were obtained with

diphenyl chlorophosphate (DPCP) and 5,5-dimethyl-2-oxo-2-chloro-1,3,2-dioxa-phosphinane (DMOCP) as coupling agents ranging from 80-90% and 95-100%, respectively (Tab. 1). Of all tested conditions and reagents, we finally selected DMOCP as the activator of choice for the condensation of both *H*-phosphonate and *H*-phosphinates **3**, and **6** in acetonitrile-pyridine mixture. The only difference was in the coupling time (Tab. 2).

Table 2. Optimized condensation conditions for coupling of *H*-phosphonate and *H*-phosphinate monomers **3**, and **6**.

Monomer	*Solvent mixture - 0.1M monomers*	*Activating agent*	*Solvent mixture - 0.3M activating agent*	*Reaction time*
H-phosphonate	ACN/pyridine 1:1	DMOCP	ACN/pyridine 95:5	5 min
*H-phosphinates **3**, and **6***	ACN/pyridine 1:1	DMOCP	ACN/pyridine 95:5	10 min

1.2.2 Oxidation

The first oxidation procedure of *H*-phosphonate/phosphinate linkages was performed with 0.1 M iodine in pyridine-water mixture (98:2) [8, 9]. LC-MS analysis confirmed very different reactivity and stability of both internucleotide linkages. In contrast to the quantitative oxidation of the *H*-phosphonate linkage to phosphodiester, the *H*-phosphinate linkage was completely hydrolyzed releasing nucleoside *O*-methyl-(*H*)-phosphinate. LC-MS revealed only the presence of 2´-deoxycytidine; no desired dimer was detected. It was clear that this linkage is extremely sensitive to water under basic conditions and that anhydrous oxidation conditions could have improve the oxidation process. For further experiment we replaced original pyridine-water (98:2) mixture for pyridine-methanol (50:50) mixture. Since the oxidation of *P-H* moiety of **11** with iodine proceeded *via* reactive *P-I* intermediate **12**, the replacement of water for methanol resulted in the formation of methyl ester **13b** which was subsequently demethylated with thiophenol-triethylamine-DMF mixture to give the desired product **13a** (Scheme 3). The iodine-methanol-pyridine mixture quantitatively oxidized *H*-phosphonate linkage, however, *H*-phosphinate bond was oxidized in range of 75-85%. Since the coupling

yield less than 90% is practically unusable for the synthesis of oligonucleotides we turned our attention to other oxidation mixtures.

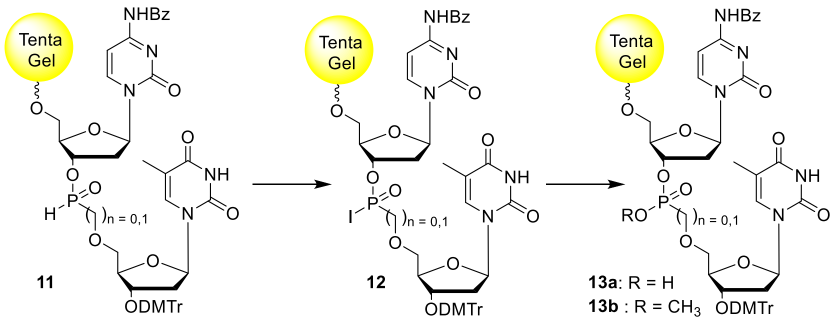

Scheme 3. Oxidation of internucleotide linkage mediated with iodine.

We examined an alternative approach based on the silylation of the *P=O* moiety in dimer **91** (formation of *P-O-SiMe*$_3$ bond) to form a P^{III} intermediate **14** followed by oxidation with the phosphoramidite protocol-based oxidizing agents [10]. Thus, several silylating agents, as *N,O*-bis(trimethylsilyl)acetamide (BSA), *N,O*-bis(trimethylsilyl)trifluoroacetamide (BSTFA), chlorotrimethylsilane and their mixture in combination with commonly used oxidizing agents as (*tert*-butyl hydroperoxide, 2-(phenylsulfonyl)-3-(3-nitrophenyl)oxaziridine (PNO), (1*R*)-(-)-(10-camphorsulfonyl)oxaziridine (CSO), and (-)-(8,8-dichlorocamphorsulfonyl) oxaziridine (DCSO), were used for the oxidation step to obtain the desired dimer **13a** (Scheme 4). We carried out many experiments with various reagents and arrangements, such as separate delivery of the silylating mixture onto the column first, followed by the oxidation agent. The combination of BSTFA with CSO or DCSO provided identical extent of the oxidation of *H*-phosphonate and *H*-phosphinate linkages within one hour, but with low yields ranged between 70-80% suggesting a partial bond cleavage.

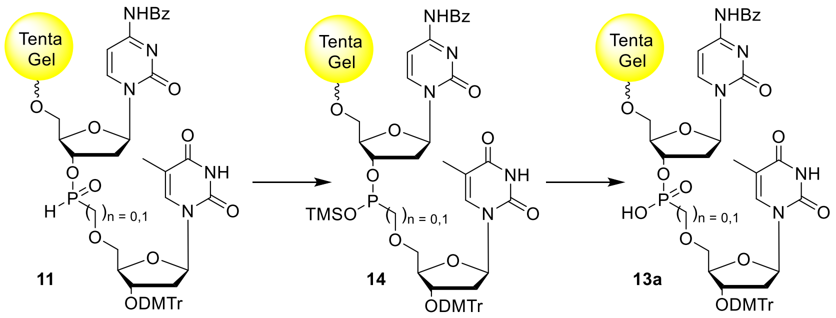

Scheme 4. Oxidation of internucleotide linkage *via* P^{III} intermediate **14**.

Another possible alternative for the oxidation of *H*-phosphonate/phosphinate linkages in oligonucleotides used was the Atherton-Todd reaction [11-13]. In this reaction, the internucleotide linkage of the *H*-phosphonate dimer **11** is oxidized with carbon tetrachloride under basic conditions to the corresponding *P-Cl* intermediate **15**, which then reacted with a nucleophile (*e.g.*, water, alcohol) to form the appropriate dimers **13a**,**b** and **16** (Scheme 5).

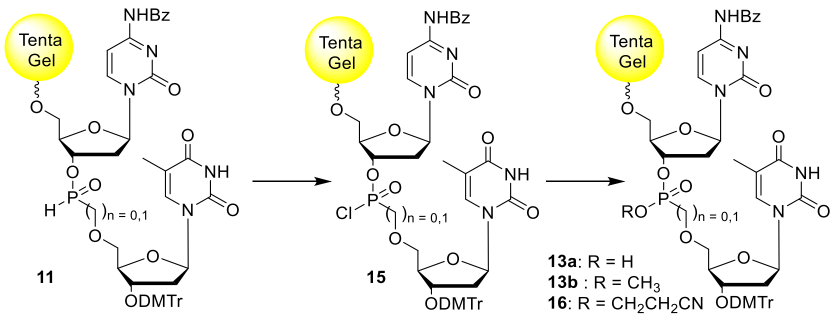

Scheme 5. Oxidation of internucleotide linkage using Atherton-Todd reaction.

We tried a water containing oxidation mixture, but also as in case of iodine-mediated oxidation, the *H*-phosphonate linkage was oxidized almost quantitatively and *H*-phosphinate bond was hydrolyzed much faster than oxidized. For Atherton-Todd reaction it is known that an alcohol can also act as a nucleophile [14]. The replacement of water for methanol significantly increased the efficiency of the oxidation mixture on the test dimer to 90-95% in the case of *H*-phosphinate and maintained the quantitative conversion of *H*-phosphonate. Our further research was focused on the composition of the oxidation mixture (CCl_4-base-alcohol), especially with respect to bases and their basicity/nucleophilicity. The replacement

of pyridine for 1-methylimidazole or 1-methylimidazole-triethylamine mixture, led to a quantitative oxidation of *H*-phosphonate and *H*-phosphinate linkages (Fig. 3).

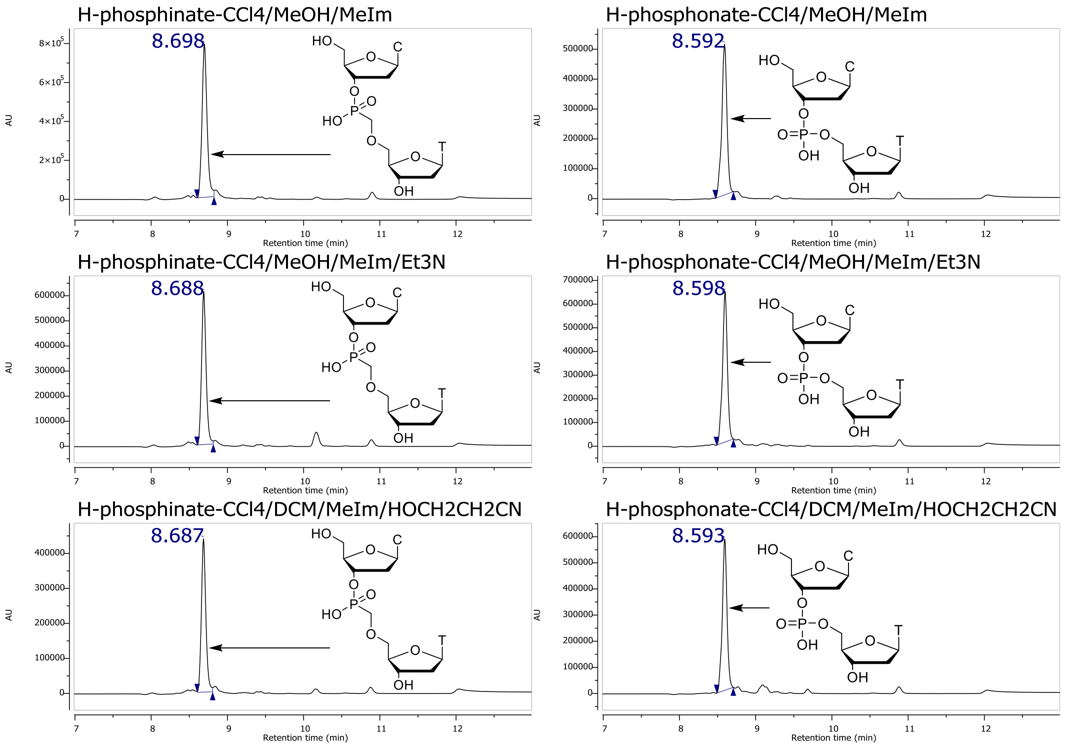

Figure 3. HPLC profiles of the crude d(CT) dimers prepared from solid support-attached dimers **11** under various oxidation conditions.

We found that methanol and 3-hydroxypropionitrile as nucleophiles in the oxidation mixture exhibited identical reactivity to provide methyl **13b** and 2-cyanoethyl **16** esters, respectively. Whereas the removal of 2-cyanoethyl ester group of **16** is accomplished *via* β-elimination in gaseous ammonia, the demethylation of **13b** to **13a** was performed with thiophenol-triethylamine-DMF mixture. In case of 3-hydroxypropionitrile-based oxidation mixture we solved a problem of homogeneity of oxidation mixture. 3-Hydroxypropionitrile is immiscible with tetrachloromethane so DCM has to be added as a co-solvent.

To select a necessary oxidizing time, all oxidation procedures were performed for 5, 10, 15, 20, 30, and 60 min. Of all oxidation mixtures tested, we succeeded in the selection of three mixtures that were applicable to *H*-phosphonate and *H*-phosphinate linkages, differing only in the oxidation time. All tested mixtures

are shown in Table 3 together with (i) yields of the dimer **13a** related to the starting nucleoside attached to TentaGel, and (ii) the time required for oxidation. The obtained results showed the different reactivity of *H*-phosphonate/*H*-phosphinate linkages in the dimer **11** toward oxidation.

Table 3. Tested oxidation mixtures with yields and oxidation times for *H*-phosphonate and *H*-phosphinate linkages in dimer **11**.

Oxidation mixture	*H-phosphonate monomer - yield / time of oxidation*	*H-phosphinate monomer **3** - yield / time of oxidation*
0.1M I_2 in Py/H_2O (98:2)	99-100% / 5 min	0% / 60 min
0.1M I_2 in Py/H_2O (98:2)	99-100% / 5 min	75-80% / 60 min
1M tBuOOH in DCM/BSTFA (9:1)	30-50% / 60 min	10-30% / 60 min
0.5M PNO in ACN/BSTFA (9:1)	40-60% / 60 min	20-35% / 60 min
0.5M CSO or DCSO in ACN/BSTFA (9:1)	90-95% / 15 min	60-65% / 30 min
CCl_4/MeOH/MeIm (7:2:1)	99-100% / 15 min	99-100% / 30 min
CCl_4/MeOH/MeIm/Et_3N (7:2:0.5:0.5)	99-100% / 10 min	99-100% / 20 min
0.1M $HOCH_2CH_2CN$ in CCl_4/DCM/MeIm (3:1:1)	99-100% / 15 min	99-100% / 30 min

1.2.3 Amidation

Encouraged by success in previously described oxidative coupling and based on the fact that the primary or secondary amines are most commonly used as a nucleophile in Atherton-Todd reaction [12, 13], we examined the preparation of electroneutral oligonucleotides with non-bridging *P-N* bond by oxidative amidation of *P-H* moiety in *H*-phosphinate linkage. Natural oligonucleotides with a phosphoroamidate arrangement were already prepared, and their hybridization properties studied [11, 15, 16]. We investigated differences between phosphoro-amidate and phosphonoamidate linkages. Two amines, the primary *N*,*N*--dimethylethylenediamine which was already incorporated into phosphoroamidate oligonucleotides [15, 16] and morpholine as a representative of secondary amines were selected. The morpholino six-membered ring could exhibit interesting steric properties if built into oligonucleotides. The mechanism of the amidation is

essentially the same as for *P-H* to *P=O* oxidation – first the *P-Cl* intermediate **15** is formed by reaction *H*-phosphinate dimer **11** with tetrachloromethane followed by the nucleophilic attack of an amine to produce the desired *P-N* dimer **17a**,**b** (Scheme 6). The amidation mixture used for this reaction was more simple than the oxidation mixture for the preparation of *P=O* bond, because the amine which was acting in the reaction as a nucleophile is simultaneously hydrogen chloride scavenger.

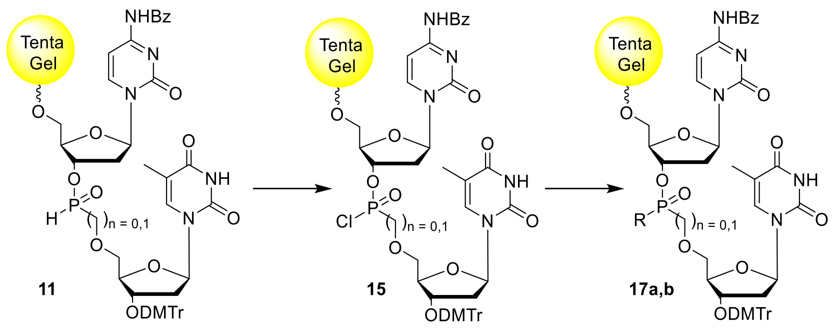

Scheme 6. Amidation of internucleotide linkage using Atherton-Todd reaction.

We tested a various ratio of tetrachloromethane and primary or secondary amines in the mixture and time for amidation. We achieved varying degree of conversion at various reaction time used (5, 10, 15, 20, 30, and 60 min), but with a ratio of 6:4 (CCl_4/amine) a quantitative amidation in 30 min was achieved for *H*-phosphonate and *H*-phosphinate linkages (Fig. 4). In case of the secondary amine, we detected a by-product which originated from hydrolysis of the *P-N* linkage with dichloroacetic acid and traces of water present in deblocking solution. These findings highlighted again the different stability of *H*-phosphonoamidate *–O-P(NR´R´´)(O)-O-* and *H*-phosphinoamidate *–CH$_2$-P(NR´R´´)(O)-O-* linkages. Whereas the former linkage was hydrolyzed from 1.74% to provide product **13a**, the extent of hydrolysis of the latter bond was 14.41%. Interestingly, the *H*-phosphonoamidate and *H*-phosphinoamidate linkages built from the primary amine (*N,N*-dimethylethylenediamine) exhibited complete hydrolytic stability.

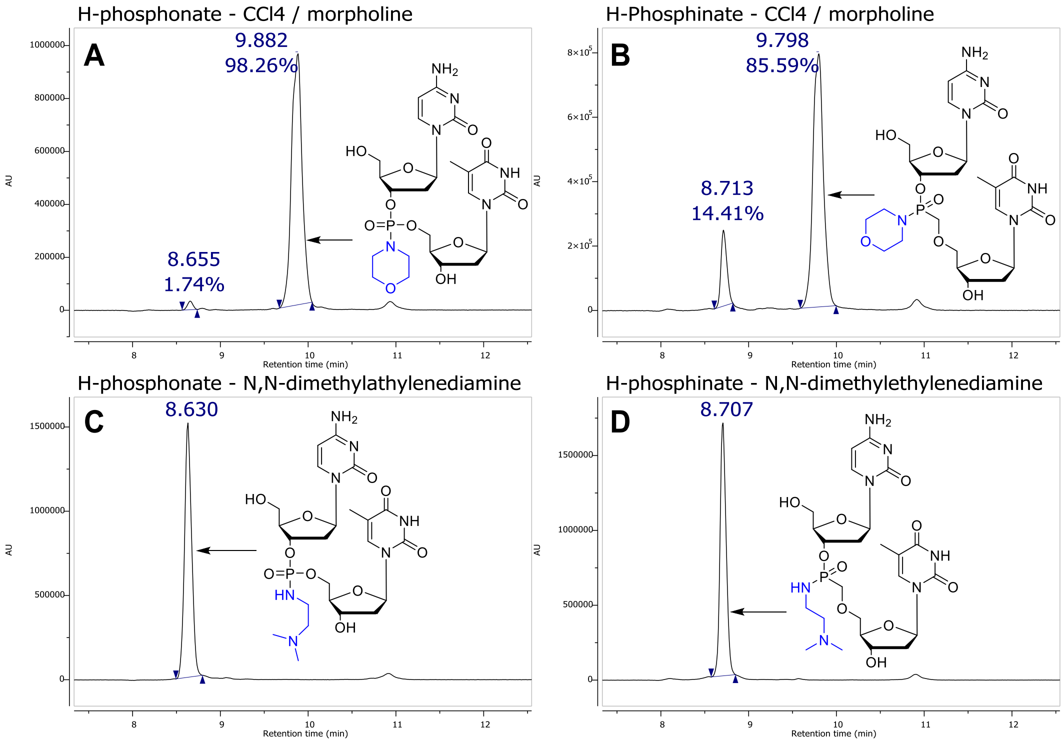

Figure 4. HPLC profiles of crude modified d(CT) dimers obtained after deprotection of **17a** (**A**, **B**), and **17b** (**C**, **D**).

The reason for that could be seen in a low electron density on the nitrogen atom of the *P-NH* moiety which prevents the protonation of the amide nitrogen atom by dichloroacetic acid as the first step of the hydrolytic mechanism; the protonation is necessary for the subsequent nucleophilic attack with water molecule.

The results obtained on amidation of *H*-phosphonate and *H*-phosphinate linkages are summarized in Table 4.

Table 4. Conditions for amidation of *H*-phosphonate and *H*-phosphinate linkages.

Amidation mixture (30min reaction time)	*H-phosphonate monomer **83** – yield of product / side product*	*H-phosphinate monomer **25a** – yield of product / side product*
CCl_4/morpholine/DCM (3:2:5)	98 / 2	85 / 14
*CCl_4/*N,N-*dimethylethylenediamine/DCM (3:2:5)*	100 / 0	100 / 0

1.2.4 Sulfurization

The sulfurization (*P-H* → *P-SH*) of *H*-phosphonate and *H*-phosphinate linkages to *H*-phosphonothioate and *H*-phosphinothioate bond, respectively, was carried out using two methods. The first, employing elemental sulfur in various solvents and in the presence of bases [17] provided directly dimer **18** from **11**. The second method based on sulfur-transferring reagent (*N*-[(2-cyanoethyl)sulfanyl] succinimide, CSS) [18, 19] which upon reaction with *P-H* bond of **11** generated uncharged *S*-(2-cyanoethyl) ester **19** in which the cyanoethyl group was removed by β-elimination to afford phosphonothioate **18** (Scheme 7).

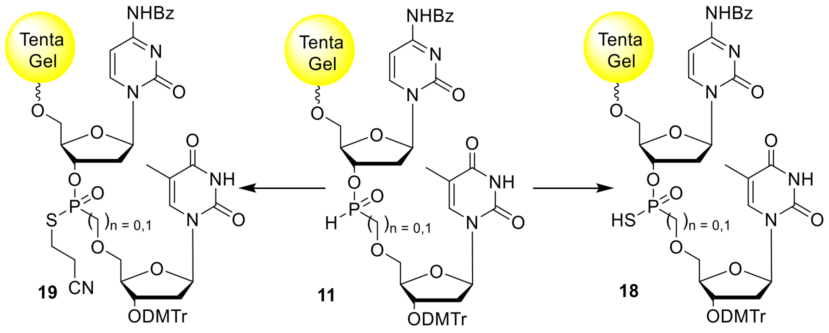

Scheme 7. Sulfurization of internucleotide linkage using elemental sulfur producing charged dimer **18**, or sulfur-transferring reagent producing S-cyanoethyl ester group on phosphorus atom of dimer **19**.

Sulfurization of **11** performed with 90% saturated solution of elemental sulfur in pyridine provided within 20 min quantitatively desired products **18** (we checked various reaction times up to 60 min). This type of sulfurization may be used only at the end of the oligonucleotide synthetic cycle. To examine a sulfur-transferring reagent (CSS) which would allow sulfurization after each synthetic cycle we prepared CSS according to the published procedure [19]. Thus the sulfurization performed with 0.2M CSS in a mixture of acetonitrile and silylating agent BSTFA [18] (29:1) provided within 30 min quantitatively the dimer **19** with the *S*-(2-cyanoethyl) ester group. Because gaseous ammonia at 0.7 MPa did not eliminate the *S*-(2-cyanoethyl) group in **19** (in contrast to *O*-(2-cyanoethyl) ester), we performed the β-elimination with 1M DBU in acetonitrile for 3 minutes. Then the TentaGel was exposed to gaseous ammonia to remove acyl-protecting groups and

to cleave the product from TentaGel. The HPLC profiles of the products **18** obtained with two sulfurization procedures were identical (Fig. 5).

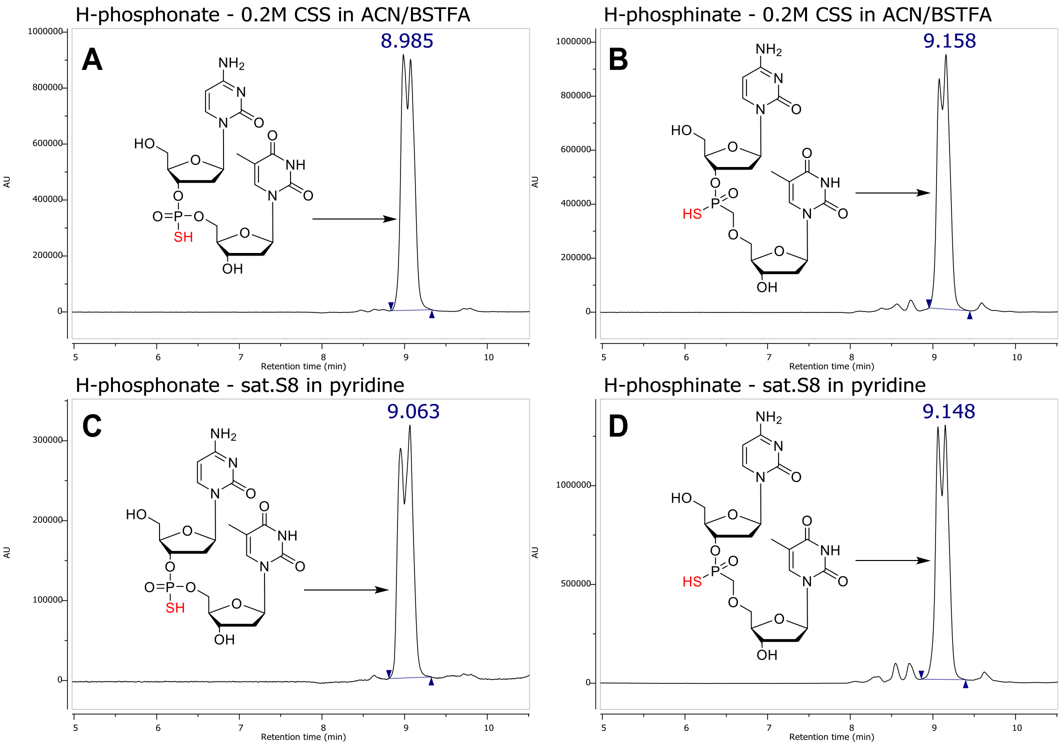

Figure 5. HPLC profiles of crude modified d(CT) dimers obtained after deprotection of **18** (**C**, **D**) and **19** (**A**, **B**).

Table 5. Conditions for sulfurization of *H*-phosphonate and *H*-phosphinate linkages in dimers **11**.

Sulfurization mixture	*H-phosphonate monomer - yield / time of sulfurization*	*H-phosphinate monomer **3** – yield / time of sulfurization*
Pyridine saturated with S_8	100% / 20 min	100% / 20 min
0.2M CSS in ACN/BSTFA (29:1)	100% / 30 min / additional DBU treatment	100% / 30 min / additional DBU treatment

Conditions for sulfurization of *H*-phosphonate and *H*-phosphinate linkages in dimers **11** are summarized in Table 5, together with reaction time, yield, and additional treatment.

1.2.5 Summary of oxidation procedures

Study on coupling reactivity of monomers **3** and capability of the formed *H*-phosphinate *3´-O-CH₂-P(O)(H)-O-5´* linkage to be oxidized, sulfurized, and/or amidated provided excellent results.

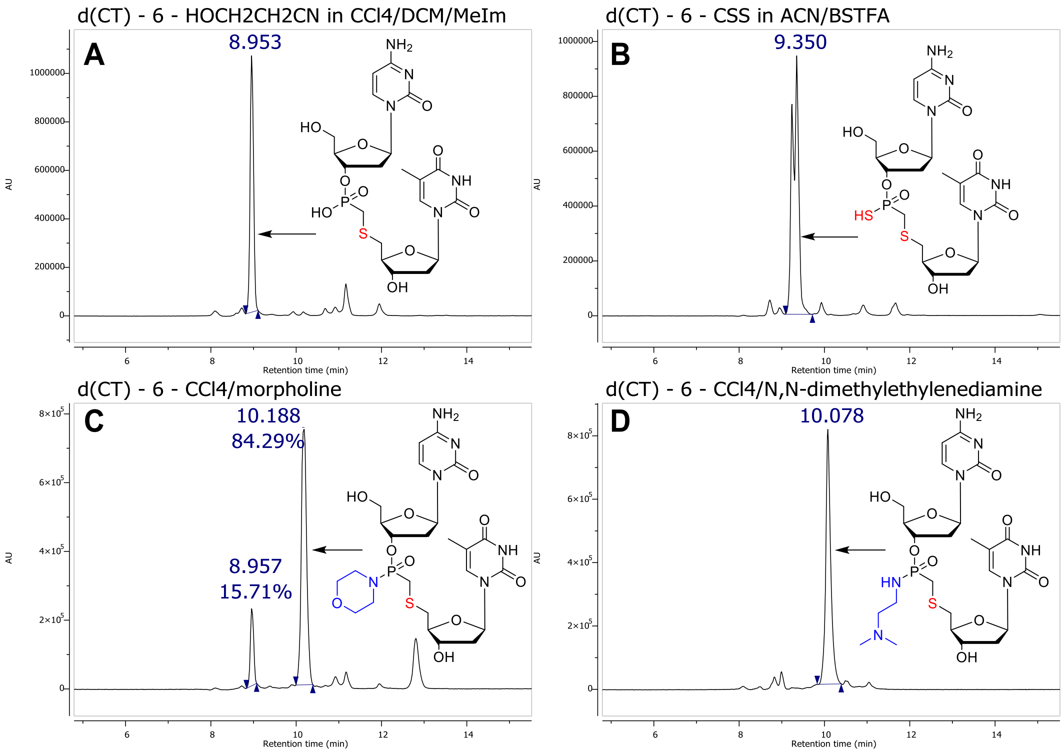

Figure 6. HPLC profiles of the crude d(CT) dimers with *3´-O-P-CH*$_2$*S-5´* internucleotide linkages (prepared from monomer **6**).

Similar results we also obtained with monomers **6** whose 5´-oxygen atom was replaced with sulfur. We did not observe a significantly different coupling efficiency, only the oxidation, sulfurization, and amidation of the *3´-O-P(O)(H)-CH*$_2$*-S-5´* linkage showed a slight increase of the unspecified by-products (Fig. 6).

In Table 6 all successfully used oxidation/sulfurization/amidation mixtures together with type of modified linkages are summarized.

Table 6. Summary of oxidation/sulfurization/amidation mixtures and their abbreviations (*R*, *R´* - depend on used amine).

Mixture		3´O-P(O)(H)-O5´	3´O-P(O)(H)-CH_2-O5´	3´O-P(O)(H)-CH_2-S5´
Ox-A	CCl_4/MeOH/MeIm (7:2:1)	*3´O-P(O)(OH)-O5´*	*3´O-P(O)(OH)-CH_2-O5´*	*3´O-P(O)(OH)-CH_2-S5´*
Ox-B	CCl_4/MeOH/MeIm/ Et_3N (7:2:0.5/0.5)	*3´O-P(O)(OH)-O5´*	*3´O-P(O)(OH)-CH_2-O5´*	*3´O-P(O)(OH)-CH_2-S5´*
Ox-C	0.1M $HOCH_2CH_2CN$ in CCl_4/DCM/MeIm (3:1:1)	*3´O-P(O)(OH)-O5´*	*3´O-P(O)(OH)-CH_2-O5´*	*3´O-P(O)(OH)-CH_2-S5´*
Ox-D	Py sat. with S_8	*3´O-P(O)(SH)-O5´*	*3´O-P(O)(SH)-CH_2-O5´*	*3´O-P(O)(SH)-CH_2-S5´*
Ox-E	0.2M CSS in ACN/BSTFA (29:1)	*3´O-P(O)(SH)-O5´*	*3´O-P(O)(SH)-CH_2-O5´*	*3´O-P(O)(SH)-CH_2-S5´*
Ox-F	CCl_4/morpholine/ DCM (3:2:5)	*3´O-P(O)(NRR´)-O5´*	*3´O-P(O)(NRR´)-CH_2-O5´*	*3´O-P(O)(NRR´)-CH_2-S5´*
Ox-G	CCl_4/*N,N*-dimethylethylene-diamine/DCM (3:2:5)	*3´O-P(O)(NRR´)-O5´*	*3´O-P(O)(NRR´)-CH_2-O5´*	*3´O-P(O)(NRR´)-CH_2-S5´*
Ox-H	CCl_4/*N,N,N´*-trimethylethylene-diamine/DCM (3:2:5)	*3´O-P(O)(NRR´)-O5´*	*3´O-P(O)(NRR´)-CH_2-O5´*	*3´O-P(O)(NRR´)-CH_2-S5´*

1.3 Stability of modified internucleotide linkages

During the optimization of the condensation and oxidation steps, we observed a different behavior of both *H*-phosphonate and *H*-phosphinate types of internucleotide linkages. Therefore we decided to examine stability of linkages using a series of modified d(CT) dimers. All subsequent experiments were performed on HPLC/MS at regular time intervals, namely 3, 6, 9, and 12 hours. The regularity was ensured by an autosampler, which injected regularly the aliquot of the same samples.

1.3.1 Stability of non-bridging P-SH bond

We found that aqueous 0.05M sodium periodate desulfurated within 12 h almost completely (~92%) phosphorothioate *3´-O-P(O)(SH)-O-5´* linkage in d(CT) dimer

to the phosphodiester *3´-O-P(O)(OH)-O-5´* bond without any bond cleavage. On the other hand, the phosphonothioate *3´-O-P(O)(SH)-CH*$_2$*-O-5´* linkage in d(CT) dimer was at completely cleaved by sodium periodate releasing 2´-deoxycytidine and thymidine-5´-*O*-methylphosphonate (Fig. 7).

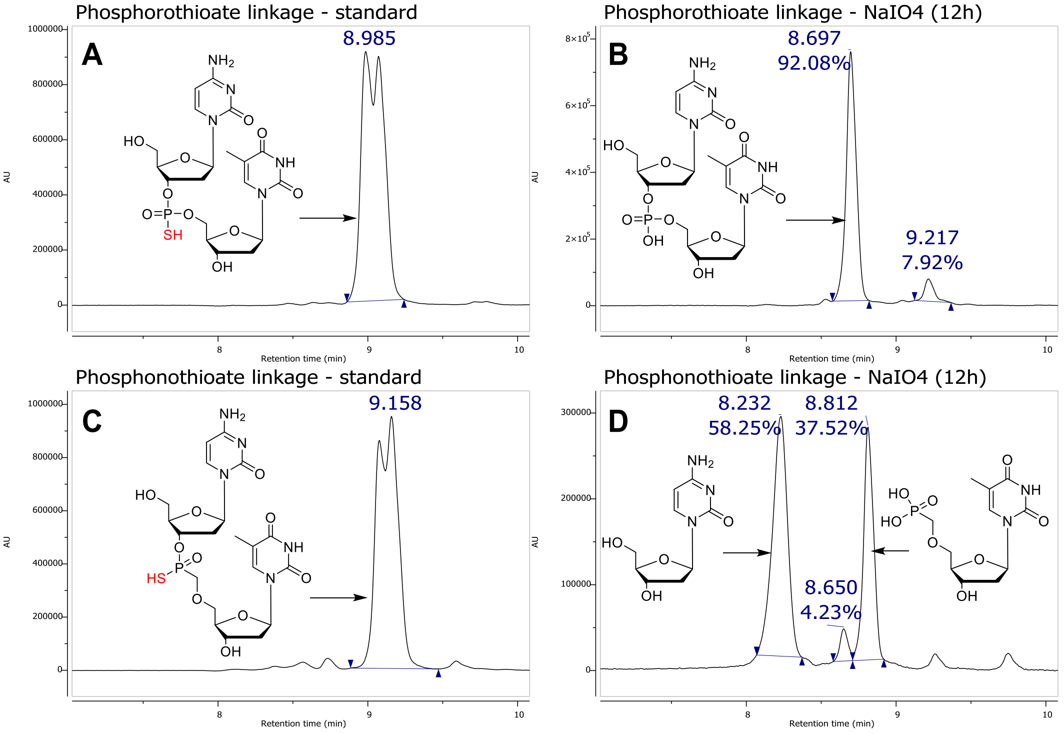

Figure 7. Hydrolytic stability of d(CT) dimers with phosphorothioate and phosphonothioate linkages in the presence of $NaIO_4$.

As we expected the treatment of sodium periodate with d(CT) dimer with *3´-O--P(O)(OH)-CH*$_2$*-S-5´* linkage led to a quantitative conversion to *S*-oxide without any *3´-O-P(O)(OH)-CH*$_2$*-S(O)-5´* bond cleavage. On the other hand d(CT) dimer with *3´-O-P(O)(SH)-CH*$_2$*-S-5´* linkage was partially desulfurized (*P-SH* → *P-OH*) providing *S*-oxide dimer as in the previous case, and partially cleaved releasing 2´-deoxycytidine. The obtained results suggested that the substitution of oxygen for sulfur in the 5'-position of the phosphonate linkage significantly increased the stability of this linkage against hydrolytic cleavage (Fig. 8).

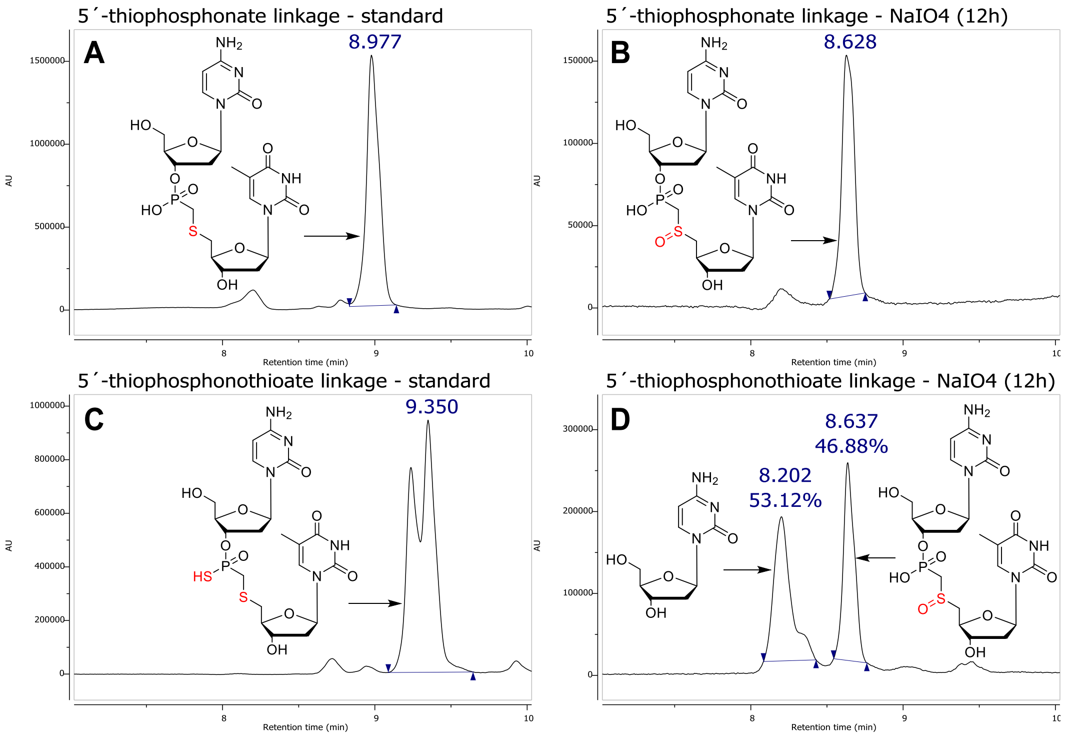

Figure 8. Hydrolytic stability of d(CT) dimers with *3´-O-P(O)(OH)-CH$_2$-S-5´* and *3´-O-P(O)(SH)-CH$_2$-S-5´* linkages in the presence of $NaIO_4$.

As another type of oxidation agent we tested 0.1M solution of iodine in water-acetonitrile-pyridine (1:1:1) mixture. Surprisingly, only desulfurization of both phosphorothioate and phosphonothioate linkages to phosphodiester and phosphonate bonds, respectively, proceeded in quantitative yields within 5 minutes without evidence of the cleavage of the internucleotide linkages (Fig. 9). In contrast to sodium periodate, the use of iodine showed no difference between reactivity of phosphorothioate and phosphonothioate linkages.

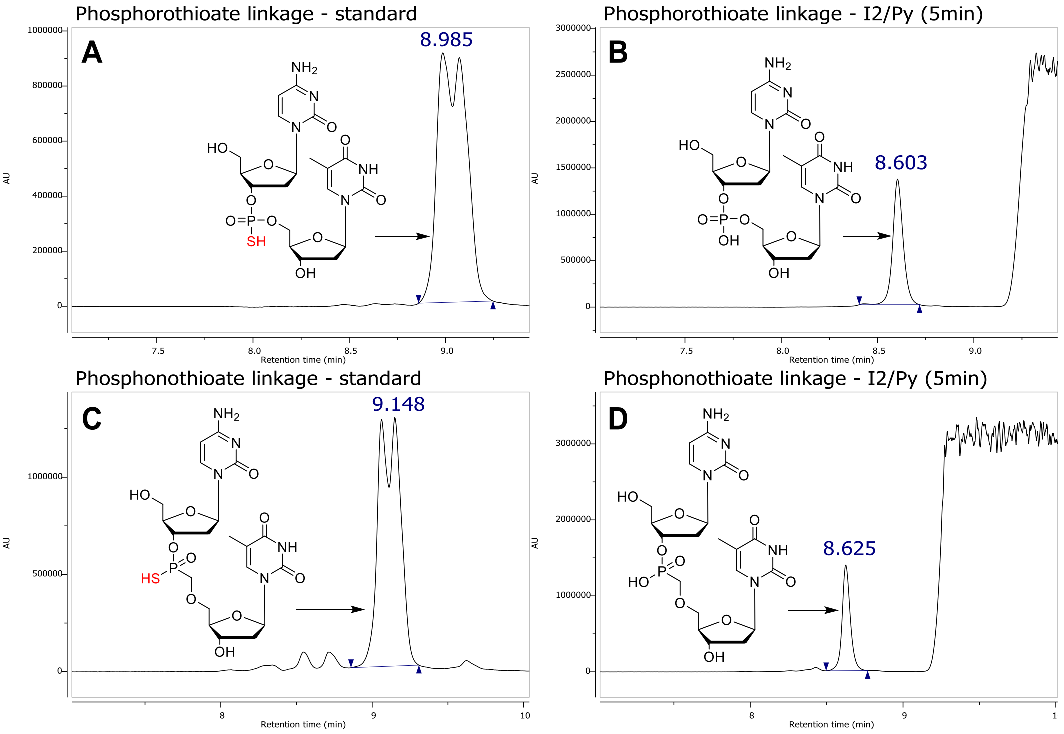

Figure 9. Hydrolytic stability of d(CT) dimers with phosphorothioate and phosphonothioate linkages in the presence of 0.1M solution of iodine in water-acetonitrile-pyridine (1:1:1) mixture.

In view of these results, we exposed these oxidation conditions to the d(CT) dimer with *3´-O-P(O)(SH)-CH*$_2$*-S-5´* linkage, where the higher stability of this internucleotide bond has been observed in the case of sodium periodate. Also in this case, the HPLC profile revealed within 5 minutes complete desulfurization of *P-SH* to *P-OH* moieties without any cleavage of the internucleotide linkage.

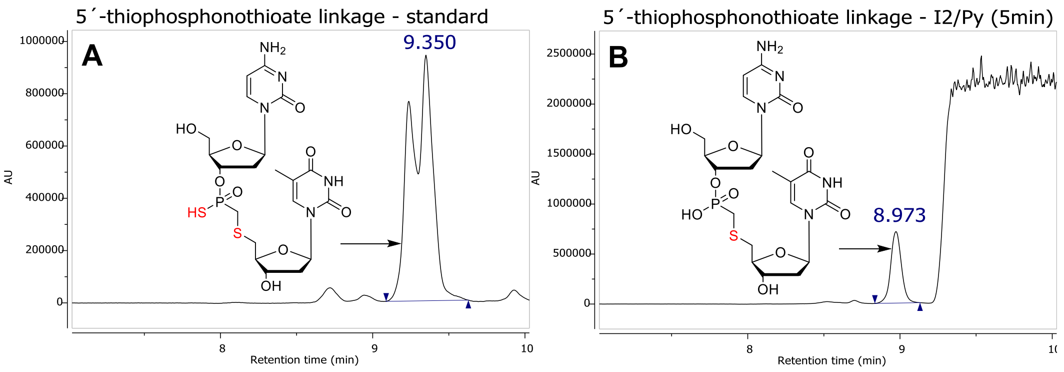

Figure 10. Hydrolytic stability of d(CT) dimer with *3´-O-P(O)(SH)-CH*$_2$*-S-5´* linkage in the presence of 0.1M solution of iodine in water-acetonitrile-pyridine (1:1:1) mixture.

Interestingly, in this case we observed no oxidation of sulfur in the 5'-position to sulfoxide moiety, even within 12 hours of treatment (Fig. 10). Our effort to find suitable conditions for sulfur oxidation at the 5'-position to obtain quantitatively sulfoxide-containing linkage failed.

1.3.2 Stability of non-bridging P-N bond

The pH stability of the prepared d(CT) dimers with non-bridging amide *P-N* bonds was performed under slightly acidic conditions at pH 5 in 50 mM $(NH_4)H_2PO_4$ and at pH 1 in 0.1M-hydrochloric acid, in both cases in 50% aqueous acetonitrile. Neither hydrolysis of non-bridging *P-N* bond (morpholidate, *N,N*-dimethyaminoethyleneamidate) nor internucleotide linkage cleavage occurred at pH 5 after 12 hours. At pH 1 no cleavage of the internucleotide linkages of d(CT) amidate dimers was observed within 9 hours.

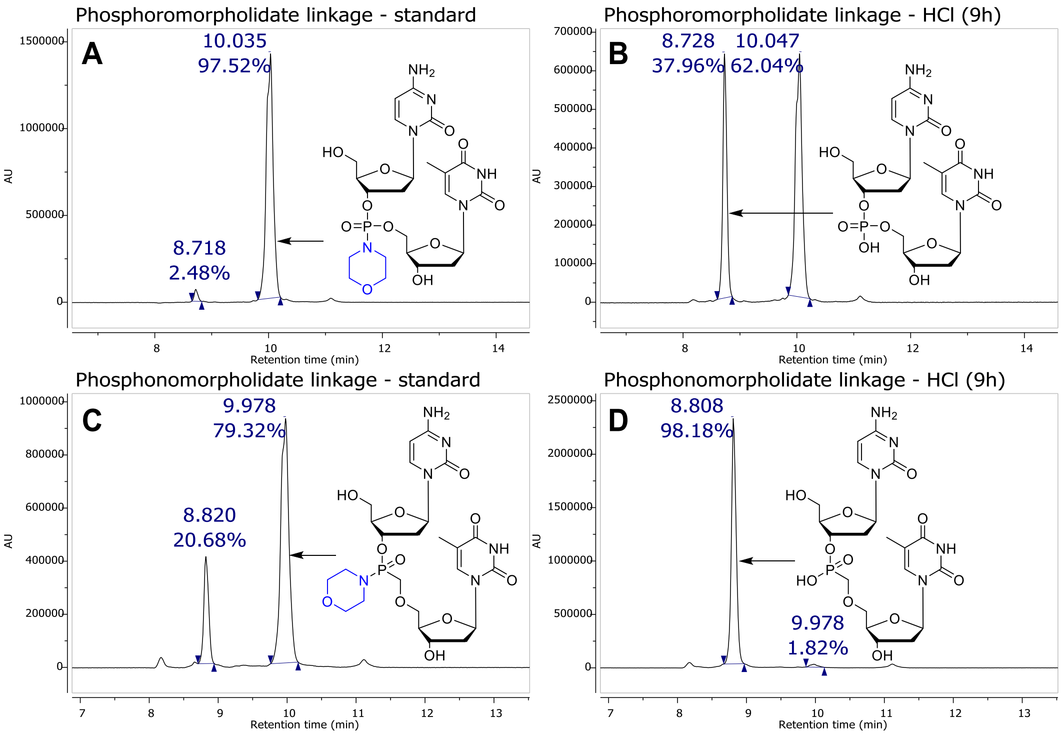

Figure 11. Hydrolytic stability of d(CT) dimers with (**B**) *3´-O-P(O)(morpholide)-O-5´* and (**D**) *3´-O-P(O)(morpholide)-CH_2-O-5´* internucleotide linkages in 0.1M-HCl.

Only non-bridging phosphoromorpholidate and phosphonomorpholidate bonds were cleaved from 38% and 98%, respectively (Fig. 11). The HPLC profile of crude d(CT) dimers with phosphoromorpholidate and phosphonomorpholidate linkages

showed 2.5% and 21% of hydrolyzed products, respectively, resulting from cleavage of the *P-N* bond during detritylation step by action of DCAA and residual water content.

In contrast to a low acid-catalyzed hydrolytic stability of the *P-N* bond of *3´-O-P(O)(morpholidate)-CH*$_2$*-O-5´* linkage (Fig. 11), we found significantly increased stability (only 18% within 9 hours) of the P-N bond of *3´-O-P(O)(morpholidate)-CH*$_2$*-S-5´* linkage where the 5´-oxygen is replaced with sulfur atom (Fig. 12). The higher hydrolytic stability could be seen in lower electron density on the morpholidate nitrogen atom which is less basic and thus its protonation as first step of the hydrolytic process is much more difficult. The HPLC profile of crude d(CT) dimer with *3´-O-P(O)(morpholidate)-CH*$_2$*-S-5´* linkage showed the presence of 16% of hydrolyzed product resulting from detritylation step, as mentioned above, and considerable amount of by-products that will increase after acid treatment (Fig. 13).

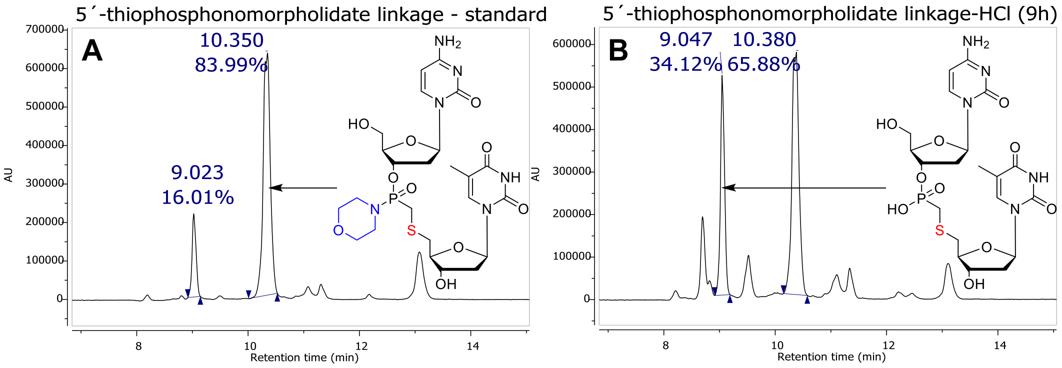

Figure 12. Hydrolytic stability of d(CT) dimers with *3´-O-P(O)(morpholide)-CH*$_2$*-S-5´* internucleotide linkages in 0.1M-HCl (**A** represent starting dimers).

Similarly to d(CT) dimers with morpholidate moiety, we also examined the hydrolytic stability of dimers with non-bridging *P*-(*N,N*-dimethylaminoethylene)amide moiety. This type of *P-NHR* amide linkage, originated from the primary amine (*N,N*-dimethylaminoethyleneamine, RNH_2) exhibited much higher hydrolytic stability under acidic conditions than P-morpholide bond in modified d(CT) dimers.

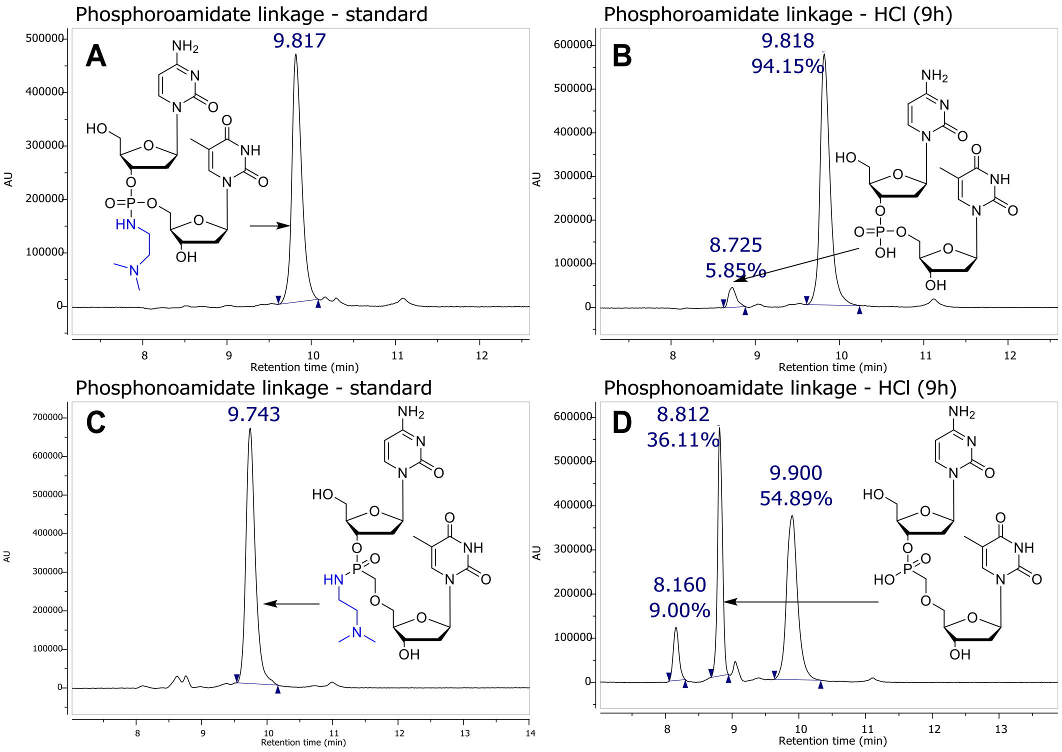

Figure 13. Hydrolytic stability of d(CT) dimers with (**B**) *3´-O-P(O)(NHR)-O-5´* and (**D**) *3´-O-P(O)(NHR)-CH₂-O-5´* internucleotide linkages in 0.1M-HCl.

Thus, after 9 hours, the extent of hydrolysis of *P-NHR* bond in *3´-O-P(O)(NHR)-O-5´* (Fig. 13; **A**, **B**), *3´-O-P(O)(NHR)-CH₂-O-5´* (Fig. 13; **C**, **D**), and *3´-O-P(O)(NHR)-CH₂-S-5´* (Fig. 14) internucleotide linkages was 6% (38), 36% (98), and 6% (34), respectively (numbers in parenthesis correspond to P-morpholidate bond cleavage).

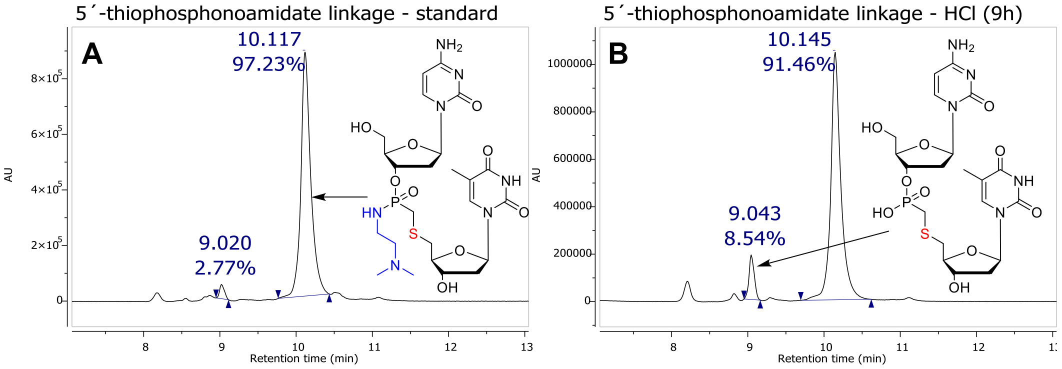

Figure 14. Hydrolytic stability of d(CT) dimers with *3´-O-P(O)(NHR)-CH₂-S-5´* internucleotide linkages in 0.1M-HCl.

1.3.3 Stability of H-phosphonate/phosphinate ester linkage

We were also interested in the stability of non-oxidized *H*-phosphonate and *H*-phosphinate ester linkages, especially in case when the next nucleotide unit has to be attached by phosphoramidite chemistry. This experiment should demonstrate possibility to combine *H*-phosphonate and phosphoramidite chemistries at the synthesis of one oligonucleotide chain.

For this study, we have chosen β-elimination type of solid support on which a dimer **20a**,**b** could be prepared (Scheme 8). The synthesis started with the *H*-phosphonate condensation of nucleoside *H*-phosphonate and *H*-phosphinate monomers, in both cases without *H*-phosphonate oxidation step, followed by phosphoramidite coupling of the standard nucleoside phosphoramidite monomer.

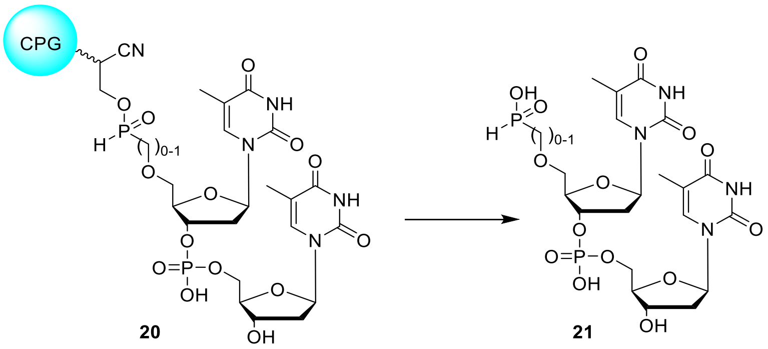

Scheme 8. Synthesis of dimer with non-oxidized 5´-end *H*-phosphonate/*H*-phosphinate linkages.

Two phosphoramidite chemistry-compatible oxidation agents were used to oxidize P^{III}-triester internucleotide linkage: (i) 1M solution of *tert*-butyl hydroperoxide in dichloromethane and (ii) 0.5M solution of CSO in acetonitrile. The *tert*-butyl hydroperoxide cleaved quantitatively the *H*-phosphonate/*H*-phosphinate linkages on the solid support and thus no dimethoxytrityl response was observed during the deblocking step. When the P^{III} linkage was oxidized with CSO, in both cases a standard dimethoxytrityl response was obtained.

The prepared dimers **20a**,**b** were released from the solid support using gaseous ammonia (0.7 MPa, 16h) to provide dimers **21a**,**b** with 5´-end *H*-phosphonate/*H*-phosphinate groups. The HPLC profiles of the solid support eluate showed the pure

desired product in the case of *H*-phosphinate and two expected products in case of *H*-phosphonate (Fig. 15). Both major products of the cleavage were recognized as desired 5´-end *H*-phosphonate dimer and the dimer without 5´-end *H*-phosphonate group. . Subsequent experiment with different β-elimination conditions used, namely 10% diethylamine in acetonitrile, already provided a pure dimer with 5´-end *H*-phosphonate (not shown).

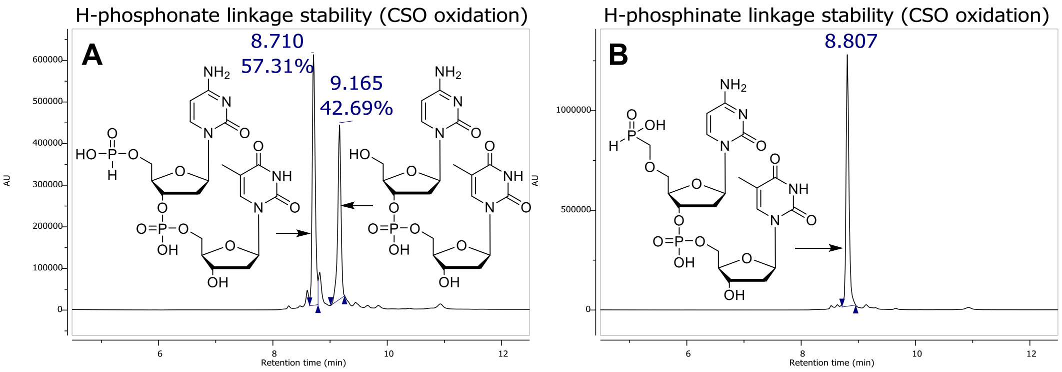

Figure 15. Stability of non-oxidized *H*-phosphonate/*H*-phosphinate linkages during phosphoramidite oxidation step.

This experiment confirmed our assumption that there is possibility to combine *H*-phosphonate and phosphoramidite chemistries at the synthesis of one oligonucleotide chain. All *H*-phosphonate/*H*-phosphinate linkages could be oxidized after last deblocking step, while the phosphoramidite monomers had to be oxidized in each phosphoramidite step. Furthermore, this approach could also be very beneficial for the synthesis of cyclic dinucleotides that are of interest through their ability to activate the immune response in the cells [20, 21].

Table 7 summarizes results on stability of modified linkages. The hydrolysis of *P-SH* bonds using $NaIO_4$ is possible in the case of phosphorothioate, but if the methylene bridge is present on the phosphorus, the linkage is cleaved. On the other hand, the hydrolysis of *P-SH* bonds in the presence of iodine and pyridine is quantitative in all linkages studied, as we verified on longer oligonucleotides (with all nucleobases). Under these conditions it is possible to quantitatively convert the oligonucleotide with phosphoro/phosphonothioate linkages into the oligonucleotide with phosphate/phosphonate linkages. This finding allows for the transformation of prepared oligonucleotide with *P-SH* bonds to equivalent of this

oligonucleotide with only *P=O* bonds. A study on the amide bonds hydrolytic stability revealed significant differences between individual types but did not bring any practical use for transformation of longer amidate oligonucleotides. The stability of *H*-phosphonate and *H*-phosphinate esters under conditions of condensation and oxidation of the phosphoramidite unit has proved to be very beneficial and will be discussed later in the text.

Table 7. Summary of internucleotide linkages stability.

Linkage	***$NaIO_4$ – 12h***	***I_2/Py – 5min***
3´-O-P(*O*)(*SH*)-O-5´	*P-SH* → *P-OH* (92%)	*P-SH* → *P-OH* (100%)
3´-O-P(*O*)(*SH*)-CH_2-O-5´	Cleavage (100%)	*P-SH* → *P-OH* (100%)
3´-O-P(O)(OH)-CH_2-S-5´	*-CH_2-S-5´* → *-CH_2-S(O)-5´* (100%)	*Stable, no oxidation*
3´-O-P(*O*)(*SH*)-CH_2-S-5´	*3´-O-P(O)(SH)-CH_2-S-5´* → *3´-O-P(O)(OH)-CH_2-S(O)-5´ (47%); Cleavage (53%)*	*P-SH* → *P-OH* (100%)
Linkage	**0.1M HCl – 9h**	
3´-O-P(O)(morpholide)-CH_2-S-5´	*P(O)(morpholide) →P(O)(OH) - (18%)*	
3´-O-P(O)(morpholide)-O-5´	*P(O)(morpholide) →P(O)(OH) - (35%)*	
3´-O-P(O)(morpholide)-CH_2-O-5´	*P(O)(morpholide) →P(O)(OH) - (78%)*	
3´-O-P(O)(NHR)-CH_2-S-5´	*P(O)(NHR) →P(O)(OH) – (6%)*	
3´-O-P(O)(NHR)-O-5´	*P(O)(NHR) →P(O)(OH) – (36%)*	
3´-O-P(O)(NHR)-CH_2-O-5´	*P(O)(NHR) →P(O)(OH) – (6%)*	
Linkage	**Phosphoramidite condensation + CSO oxidation (capping omitted)**	
3´-O-P(O)(H)-O-5´	*Stable*	
3´-O-P(O)(H)-CH_2-O-5´	*Stable*	

1.4 Preparation of modified oligonucleotides

From the knowledge gained from the dimer study, we optimized the synthesis of longer oligonucleotides, firstly modified oligothymidylate (decamers, **dT$_{10}$**). In oligothymidylate, we tried to introduce different types of modifications of internucleotide linkages using protected thymidine *H*-phosphonate, *H*-phosphinate, and phosphoramidite as monomers. The individual use of these monomers necessitated the use of various approaches for the preparation of modified oligothymidylates **dT$_{10}$1** - **dT$_{10}$7**.

1.4.1 Synthetic Protocol A

The first choice for the preparation of oligonucleotides using *H*-phosphinate monomers was their combination with *H*-phosphonate monomers using the *H*-phosphonate approach. The synthetic cycles proceeded without the capping steps and oxidation of the whole oligonucleotide chain was performed after the synthesis. Thus, the internucleotide linkage can be oxidized to *P-OH* or *P-SH* groups. On oligothymidylate **dT$_{10}$1** with three phosphonate linkages, we first tested methanol-containing oxidation mixtures (Ox-A, or Ox-B) selected previously on d(CT) dimers. The formed methyl ester groups were removed by treatment with a mixture of PhSH/Et$_3$N/DMF, the oligonucleotide was released from the solid support with gaseous ammonia and eluted from the column by 50% aqueous acetonitrile.

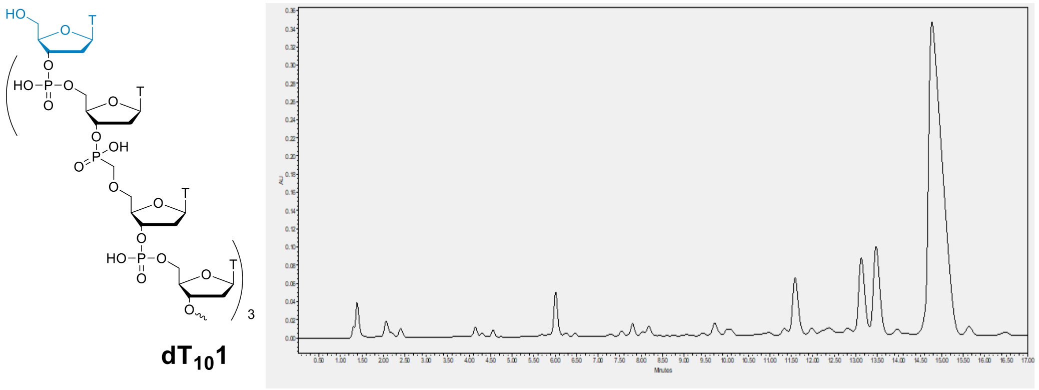

Figure 16. IEC HPLC profile of crude decamer **dT$_{10}$1**, obtained by oxidation of whole decamer after synthesis by Ox-A.

The eluate was analyzed by ion exchange chromatography (IEC HPLC), and we obtained for both oxidation conditions the identical IEC HPLC profile (Fig. 16) in which the main peak was identified as desired decamer **dT$_{10}$1** (MALDI), containing three phosphonate modifications. For detailed **Synthetic Protocol A** see Tab. 8.

Table 8. **Synthetic Protocol A** Methyl-ester cleavage procedure is only in case of MeOH oxidation mixtures.

Procedure	***Reagents***	***Vol./time***	***Solvent***
Washing	-	2 mL / 80 s	DCM
Deblocking	3% DCAA in DCM	6 mL flowing through the column /120 s	-
Washing	-	3.5 mL / 140 s	DCM
Washing	-	2 mL / 80 s	ACN
Washing	-	3 mL / 120 s	DCM
Drying	Argon	Gas flow / 160 s	-
Condensation	0.1M monomer	120 µL / 10min	ACN/Py (1:1)
(monomer + activator mixed)	0.3M DMOCP	120 µL / 10min	ACN/Py (95:5)
Washing	-	4 mL / 160 s	DCM
Washing	-	4 mL / 160 s	ACN
Washing	-	4 mL / 160 s	DCM
Drying	Argon	Gas flow / 160 s	-
END OF SYNTHETIC CYCLE			
Oxidation	Ox-A, Ox-B, Ox-D	220 µL / 30 min	-
Washing	-	6 mL / 240 s	DCM
Drying	Argon	Gas flow / 160 s	-
(Methyl-ester cleavage)	PhSH/Et_3N/DMF	0.3 mL / 4 h	-
(Washing)	-	6 mL / 240 s	ACN
(Drying)	Argon	Gas flow / 160 s	-
Cleavage from solid support	Ammonia gas at 0.7 MPa	Gas chamber / 16 h	-

The same procedure we used for preparation **dT$_{10}$2** where the backbone was fully sulfurized to give rise to the phosphonothioate/phosphorothioate linkages by the sulfurization mixture (Ox-D) used at the end of the synthesis. The crude product was analyzed on IEC HPLC (Fig. 17) and the major peak was identified as the desired phosphoro/phosphonothioate linkages-containing **dT$_{10}$2** (MALDI).

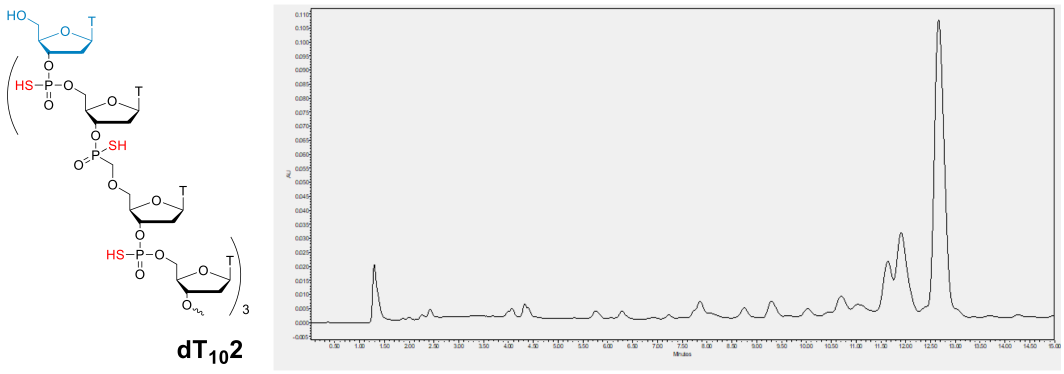

Figure 17. IEC HPLC profile of crude decamer **dT$_{10}$2**, oxidized after synthesis with Ox-D.

1.4.2 Synthetic Protocol B

When the combination of *H*-phosphonate and phosphoramidite chemistries was used for the introduction of modified and natural nucleotide units into one oligonucleotide chain, respectively, the oxidative coupling steps generating an esterified *H*-phosphonate internucleotide linkage, had to be used. The methanol-containing oxidation mixtures mentioned above for the final chain oxidation were not suitable due to low stability of the formed methyl ester phosphonate *3´O-P(O)(OCH$_3$)-CH$_2$-O5´* linkages. The random cleavage of methyl ester groups resulted in negatively charged phosphonate linkages, which can be activated in the *H*-phosphonate coupling step by DMOCP to form a mixed-anhydride, followed by the chain cleavage or branched chain growth (HPLC pattern is not shown). Therefore we used oxidation mixture where methanol was replaced with 3-hydroxypropionitrile. The formed 2-cyanoethyl ester has been shown to be stable within the subsequent synthetic cycles. Under these conditions and using capping steps we obtained a satisfactory yield of **dT$_{10}$1** (Fig. 18).

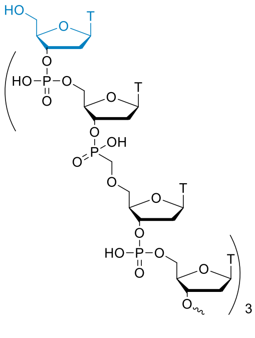

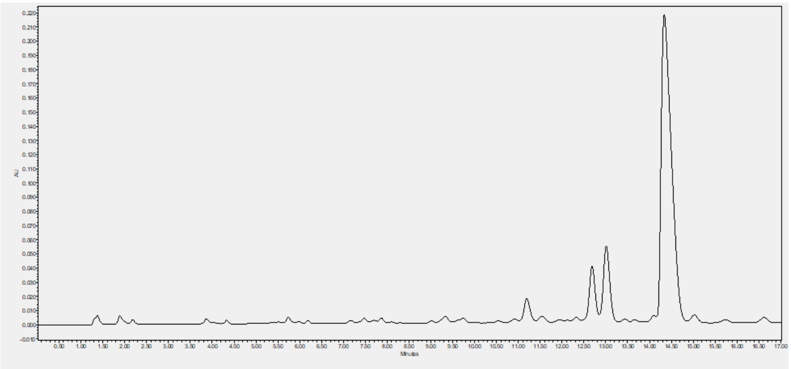

Figure 18. IEC HPLC profile of crude decamer **dT101**. *H*-phosphinate linkage oxidized with Ox-C.

Alternatively, we tested the preparation of fully sulfurized **dT102** (Fig. 33) containing three phosphonothioate *3´O-P(O)(SH)-CH2-O5´* linkages. After each *H*-phosphonate coupling step, the sulfurization mixture (Ox-E) was applied to oxidize *H*-phosphinate *3´O-P(H)(O)-CH2-O5´* bond to phosphonothioate linkage bearing *S*-(2-cyanoethyl) ester group which was stable within all steps of oligonucleotide synthesis including a capping step. In contrast to *O*-(2-cyanoethyl), the β-elimination of the *S*-(2-cyanoethyl) ester group required more nucleophilic conditions than gaseous ammonia. The oligonucleotides still attached on solid support were treated with 1M DBU in acetonitrile for 3 min to ensure a complete β-elimination of *S*-(2-cyanoethyl) ester groups.

This approach provided even slightly worse IEC HPLC profile (Fig. 19) because of accumulation of failure sequences into one region (product shorter by one unit), but the yield of the desired **dT102** was higher than at the use of fully *H*-phosphonate approach. The mentioned failure sequence was shortened by one phosphonate unit, indicating a partial cleavage of the phosphorothioate linkage, possibly during the capping step. However, this fact was not proved, since the model dimer did not show this phenomenon.

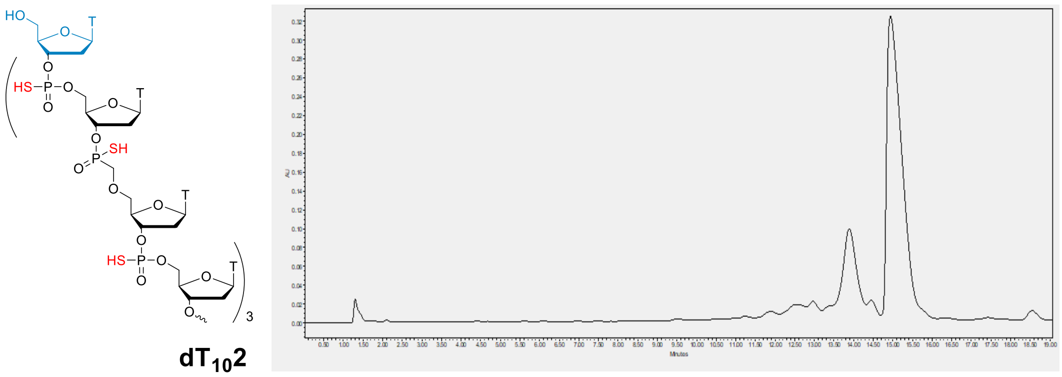

Figure 19. IEC HPLC profile of crude decamer **dT$_{10}$2**. After each *H*-phosphonate cyclus, the *H*-phosphinate linkage was sulfurized with Ox-E; after each phosphoramidite cyclus, the linkage was sulfurized with Sulfur reagent II (Glen Research).

The combination of two different chemistries of oligonucleotide synthesis opened possibility for the introduction of phosphorothioate and phosphonothioate internucleotide linkages into a particular position in the oligonucleotide, unlike the fully *H*-phosphonate approach with sulfurization/oxidation at the end of the synthesis.

These findings have led us to the preparation of **dT$_{10}$3** and **dT$_{10}$4** where the phosphoromorpholidate and phosphonomorpholidate linkages, respectively, are present at selected positions.

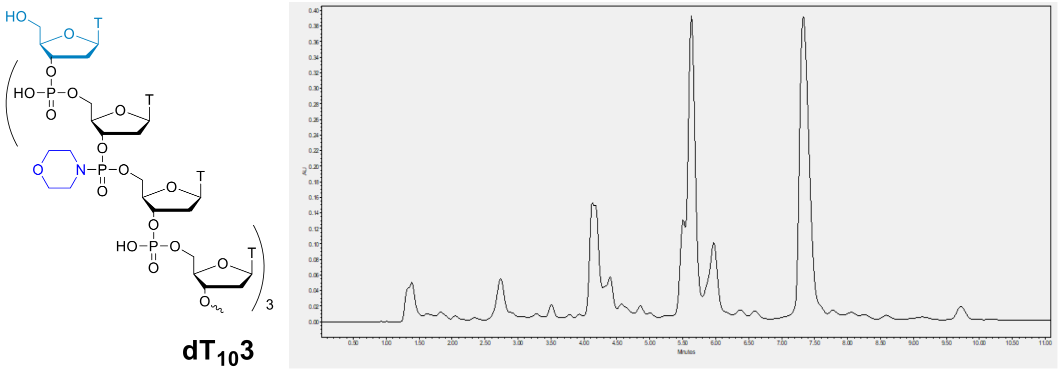

Figure 20. IEC HPLC profile of crude decamer **dT$_{10}$3**. After each *H*-phosphonate cycle, the *H*-phosphonate linkage was amidated with Ox-F; after each phosphoramidite cycle, the linkage was oxidized with *tert*-butyl hydroperoxide.

In the case of decamer **dT₁₀3**, three phosphoromorpholidate linkages originating from amidation of *H*-phosphonate bonds with Ox-F mixture, are present. IEC HPLC profile (Fig. 20) shows a large quantities of failure sequences.

Even worse results were obtained at the preparation of **dT₁₀4** (Fig. 21) containing three phosphonomorpholidate linkages originating from amidation of *H*-phosphinate bonds with Ox-F mixture. IEC HPLC profile (Fig. 21) shows again a large amount of failure sequences which came from acid-catalyzed hydrolysis of phosphonomorpholidate linkage which was repeatedly exposed to the deblocking step (deblocking solution contained 150-200 ppm of water).

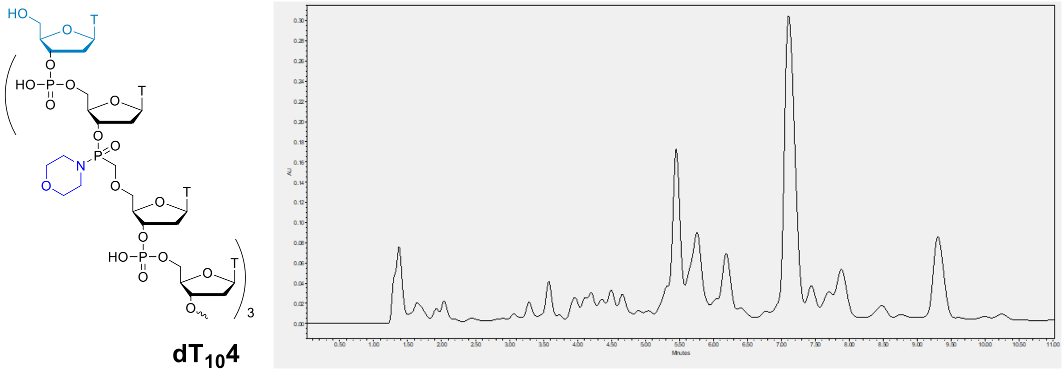

Figure 21. IEC HPLC profile of crude decamer **dT₁₀4**. After each *H*-phosphonate cycle, the *H*-phosphinate linkage was amidated with Ox-F; after each phosphoramidite cycle, the linkage was oxidized with *tert*-butyl hydroperoxide.

We also attempted to prepare two decamers using an amidation mixture where morpholine was substituted for *N*, *N*-dimethylethylenediamine, but in this case, we encountered analytical difficulties. None of the mobile phases used on IEC HPLC were able to separate these zwitter-ionic oligonucleotides. However, when *N*, *N*-dimethylethylenediamine was replaced with *N*, *N*, *N'*-trimethylethylene-diamine in the amidation mixture, and the strongly basic mobile phase was used in IEC HPLC, it was possible to separate those oligonucleotides, where the amide hydrogen atom was replaced by a methyl group.

For the synthesis of oligonucleotides that combine the *H*-phosphonate with the phosphoramidite chemistry where all internucleotide linkages are oxidized in each synthetic cycle, we have developed a **Synthetic Protocol B** (Tab. 9). This protocol

only contains procedures for *H*-phosphonate/*H*-phosphinate monomers because phosphoramidite monomers (Glen Research) have been condensed according to the standard protocol. All washing procedures used for each step are identical to those of **Synthetic Protocol A** (Tab. 8).

Table 9. **Synthetic Protocol B** Protocol for *H*-phosphonate/*H*-phosphinate monomers condensation and oxidation/sulfurization/amidation, without washing procedures (used the same washing sequences listed in Table 8) S-cyanoethyl-ester cleavage procedure is only in case of CSS sulfurization mixtures.

H-PHOSPHONATE / H-PHOSPHINATE CONDENSATION STEPS			
Procedure	**Reagents**	**Vol./time**	**Solvent**
Deblocking	3% DCAA in DCM	6mL flowing through the column / 120 s	-
Additional washing procedure for oxidized internucleotide linkage	-	6 mL / 240 s	Pyridine / methanol (95:5)
(Washing)	-	6 mL / 240 s	DCM
(Drying)	Argon	Gas flow / 160 s	-
Condensation	0.1M monomer	120 μL / 10 min	ACN/Py (1:1)
(monomer + activator mixed)	0.3M DMOCP	120 μL / 10 min	ACN/Py (95:5)
Oxidation; Sulfurization; Amidation	Ox-C; Ox-E; Ox-F; Ox-G; Ox-H CCl_4/amine	220 μL / 30 min	-
Capping	Ac_2O/Py/MeIm/THF (1:1:1:17)	0.3 mL / 3 min	
Washing	-	4 mL / 160 s	ACN
Washing	-	4 mL / 160 s	DCM
Drying	Argon	Gas flow / 160 s	-
END OF SYNTHETIC CYCLE			
(S-cyanoethyl-ester cleavage)	1M DBU in CAN	220 μL / 3 min	-
(Washing)	-	6 mL / 240 s	ACN
(Drying)	Argon	Gas flow / 160 s	-
Cleavage from solid support	Ammonia gas at 0.7 MPa	Gas chamber / 16 h	-

1.4.3 Synthetic Protocol C

Because of low yields at the preparation of phosphoroamidate/phosphonoamidate dT_{10} where synthesis was complicated by acidic hydrolysis of the amide linkages in the deblocking step, we changed the synthetic strategy to overcome these obstacles to obtain the desired oligonucleotides in a better yield.

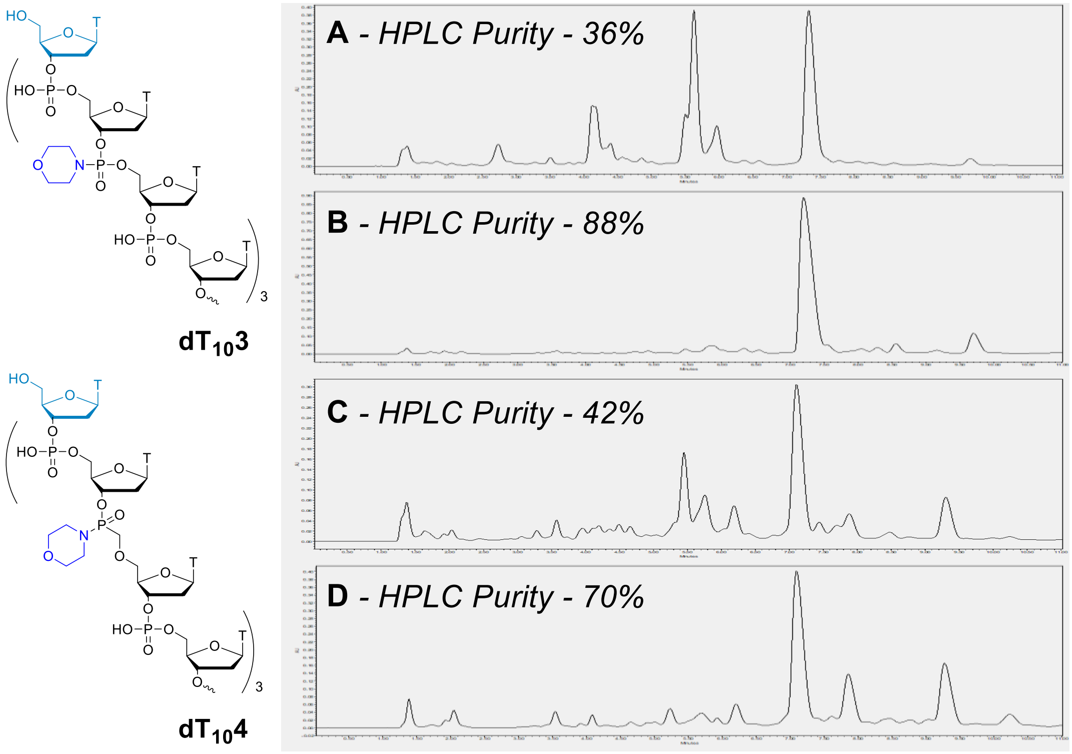

Figure 22. IEC HPLC profile of crude decamer **dT₁₀3** (**A**, **B**), and **dT₁₀4** (**C**, **D**); **A**, **C** – Synthetic Protocol A (P-H bond amidated with Ox-F); **B**, **D** – Synthetic Protocol C (P-H bond amidated with Ox-F).

We found that the oxidation procedure of the phosphoramidite chemistry need not necessarily cleave *H*-phosphonate and *H*-phosphinate linkages, when oxaziridine CSO (or DCSO) is used as an oxidant and the acetic anhydride-based capping step is omitted. Thus, all P^{III} triester internucleotide linkages generated by phosphoramidite chemistry were oxidized to P^{V} with CSO, and *H*-phosphonate/*H*-phosphinate linkages were left unoxidized and also uncleaved under these conditions. After the last deblocking step, all *H*-phosphonate/*H*-phosphinate linkages generated by the *H*-phosphonate chemistry were oxidized, sulfurized, or amidated at the same time **Synthetic Protocol C** (Tab. 10). IEC HPLC profiles

dT$_{10}$3 (Fig. 22; **A**, **B**) and **dT$_{10}$4** (Fig. 22; **C**, **D**) showed remarkable improvement of the synthesis.

Table 10. **Synthetic Protocol C** *H*-phosphonate/*H*-phosphinate monomers condensation and oxidation/sulfurization/amidation at the end of syntetic cycle. The same washing procedures used as in **Synthetic Protocol A** (Table 8).

H-PHOSPHONATE / H-PHOSPHINATE CONDENSATION STEPS			
Procedure	**Reagents**	**Vol./time**	**Solvent**
Deblocking	3% DCAA in DCM	6 mL flowing through the column / 120 s	-
Condensation (monomer + activator mixed)	0.1M *H*-phosphonate or *H*-phosphinate monomer	120 μL / 10 min	ACN/Py (1:1)
	0.3M DMOCP	120 μL / 10 min	ACN/Py (95:5)
PHOSPHORAMIDITE CONDENSATION STEP			
Procedure	**Reagents**	**Vol./time**	**Solvent**
Deblocking	3% DCAA in DCM	6mL flowing through the column / 120s	-
Condensation (monomer + activator mixed)	0.1M phosphoramidite monomer	120 μL / 5 min	ACN
	0.25M ETT	120 μL / 5 min	ACN
Oxidation P^{III} to P^{V}	0.5M CSO or DCSO in ACN	220 μL / 3.5 min	-
Washing	-	4 mL / 160 s	DCM
Drying	Argon	Gas flow / 160 s	-
END OF SYNTHETIC CYCLE			
Oxidation; Sulfurization; Amidation of P-H bond	0.1M $HOCH_2CH_2CN$ in CCl_4/DCM/MeIm; pyridine saturated with S_8; CCl_4/amine	220 μL / 30 min	-
Washing	-	6 mL / 240 s	DCM
Drying	Argon	Gas flow / 160 s	-
Cleavage from solid support	Ammonia gas at 0.7 MPa	Gas chamber / 16 h	-

By IEC HPLC profiles we compared efficacy of developed synthetic protocols for the synthesis of **dT₁₀1** (Fig. 23). The comparison of HPLC profiles demonstrated that the application of **Synthetic protocol B** provided the highest yield of **dT₁₀1**.

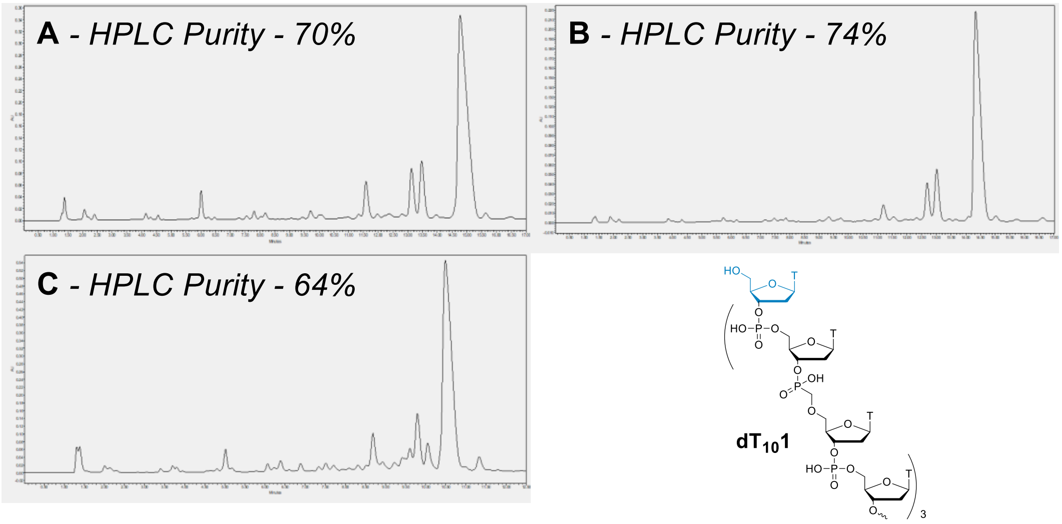

Figure 23. IEC HPLC profile of crude decamer **dT₁₀1**, prepared by: **A** - Synthetic Protocol A (*P-H* bond oxidized with Ox-A); **B** - Synthetic Protocol B (*P-H* bond oxidized with Ox-C); **C** - Synthetic Protocol C (*P-H* bond oxidized with Ox-C).

We also compared the efficiency of the sulfurization procedures. For IEC HPLC profiles of fully and partially sulfurized **dT₁₀2** and **dT₁₀5**, respectively, see Fig. 24.

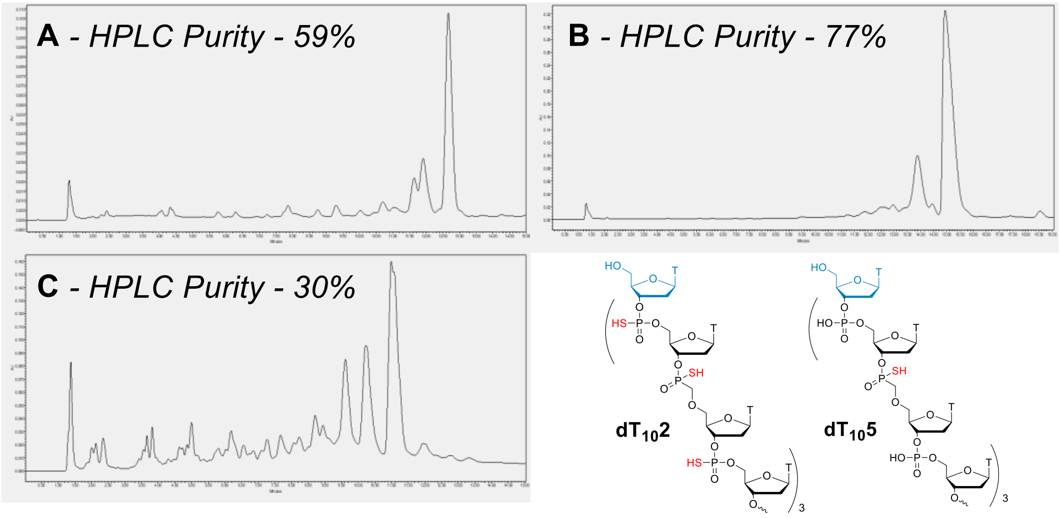

Figure 24. IEC HPLC profile of crude decamers: **A - dT₁₀2** - Synthetic Protocol A (*P-H* bond sulfurized with Ox-D); **B - dT₁₀2** - Synthetic Protocol B (*P-H* bond sulfurized with Ox-E); **C - dT₁₀5** - Synthetic Protocol C (*P-H* bond sulfurized with Ox-D).

Last but not least, we compared the effectiveness of the combined phosphoramidite method (CSO oxidation per cycle) with the *H*-phosphonate method (oxidation/ sulfurization/amidation after last cycle), where only *H*-phosphonate monomers were used (Fig. 25).

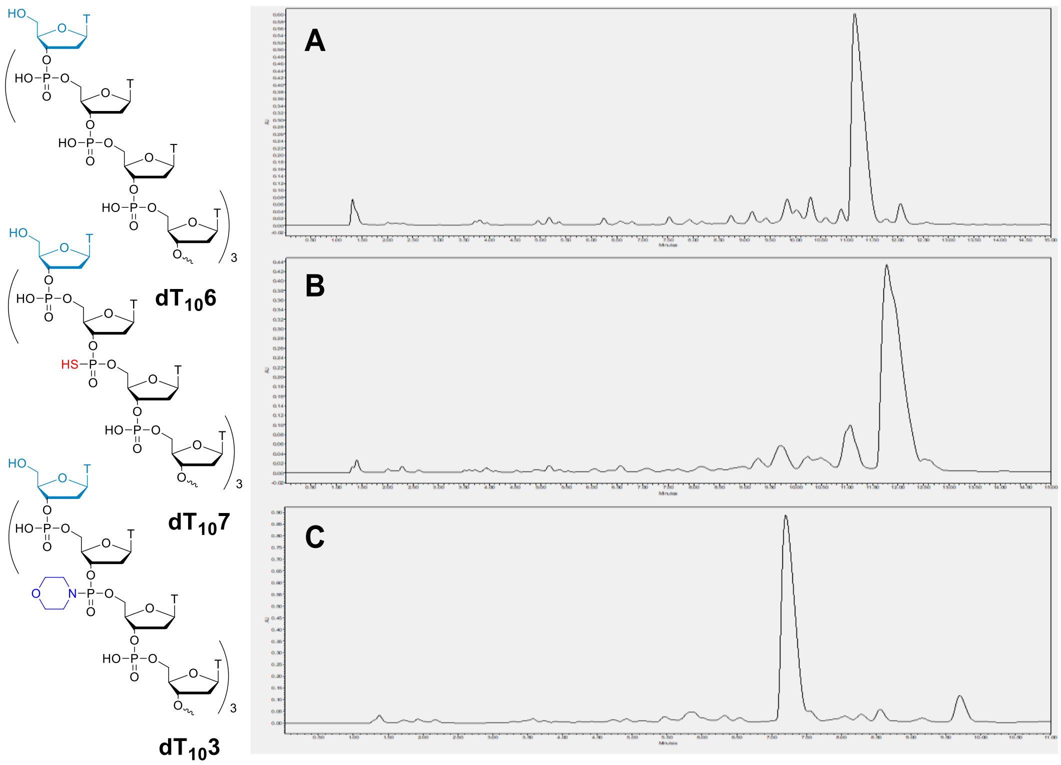

Figure 25. IEC HPLC profile of crude decamers. **A** - **dT$_{10}$6** - Synthetic Protocol C (*P-H* bond oxidized with Ox-C); **B** - **dT$_{10}$7** - Synthetic Protocol C (*P-H* bond sulfurized with Ox-D); **C** – **dT$_{10}$4** - Synthetic Protocol C (*P-H* bond amidated with Ox-F).

Table 11. The overview of oxidative methods used in Synthetic protocols for generation of modified internucleotide linkages.

Linkage	***Synthetic Protocol A***	***Synthetic Protocol B***	***Synthetic Protocol C***
3´O-P(O)(OH)-O5´	Ox-A, Ox-B, Ox-C	Ox-C	Ox-A, Ox-B, Ox-C
3´O-P(O)(SH)-O5´	Ox-D, Ox-E	Ox-E	Ox-D, Ox-E
3´O-P(O)(NRR´)-O5´	-	-	Ox-F, Ox-H
3´O-P(O)(OH)-CH$_2$-O5´;<br>*3´O-P(O)(OH)-CH$_2$-S5´*	Ox-A, Ox-B, Ox-C	Ox-C	Ox-A, Ox-B, Ox-C
3´O-P(O)(SH)-CH$_2$-O5´;<br>*3´O-P(O)(SH)-CH$_2$-S5´*	Ox-D, Ox-E	Ox-E	Ox-D, Ox-E
3´O-P(O)(NRR´)-CH$_2$-O5´	-	-	Ox-F, Ox-H

2. PHYSICOCHEMICAL PROPERTIES OF ONS

Thermal experiments were performed at 260 and 295 nm on a CARY 100 Bio UV Spectrophotometer (Varian Inc.) equipped with a Peltier temperature controller and thermal analysis software. The samples were prepared by mixing equal molar amounts of modified and natural strands to give a 4μM final concentration in 100mM NaCl, 50mM NaH_2PO_4, 1mM EDTA, pH 7.2. A heating-cooling cycle in the range 5–60 °C with a gradient of 0.2 °C min^{-1} was applied. T_m values were determined from the maxima of the first derivative plots of absorbance versus temperature (T_m± 0.5 °C). In all cases, single transition profiles were observed.

Table 12. Summary of the prepared modified oligodeoxy-nucleotides.

	Modification of internucleotide linkage	*Code*	*Sequence 5'→3'*	*T_m [°C]* Target *DNA*	*T_m [°C]* Target *RNA*
Group A	**A,T,G,C** = *3´O-P(O)-O5´*<br>**A,T,C** = *3´O-P(S)-O5´*	**ON-1**	**d(GCA TAT CAC)**	35	32
		**ON-2**	**d(GCA TAT CAC)**	29	27
		**ON-3**	**d(GCA TAT CAC)**	26	24
Group B	**A** = *3´O- P(O)-CH_2-S5´*<br>**T,C,G** = *3´O-P(O)-O5´*<br>**A** = *3´O- P(S)-CH_2-S5´*<br>**T,C** = *3´O-P(S)-O5´*	**ON-4**	**d(GCA TAT CAC)**	24	32
		**ON-5**	**d(GCA TAT CAC)**	15	23
		**ON-6**	**d(GCA TAT CAC)**	24	30
		**ON-7**	**d(GCA TAT CAC)**	14	25
Group C	**A** = *3´O-P(O)-CH_2-O5´*<br>**T,C** = *3´O-P(S)-O5´*<br>**A** = *3´O-P(S)-CH_2-O5´*<br>**T,C,G** = *3´O-P(O)-O5´*	**ON-8**	**d(GCA TAT CAC)**	38	44
		**ON-9**	**d(GCA TAT CAC)**	27	34
		**ON-10**	**d(GCA TAT CAC)**	35	41
		**ON-11**	**d(GCA TAT CAC)**	30	39
Group D	**A** = *3´O-P(NRR´)-CH_2-O5´*<br>**A** = *3´O-P(NRR´)-O5´*<br>**T,C,G** = *3´O-P(O)-O5´*	**ON-12**[1]<br>**ON-13**[2]	**d(GCA TAT CAC)**	30<br>33	29<br>31
		**ON-14**[1]<br>**ON-15**[2]	**d(GCA TAT CAC)**	26<br>29	21<br>23

[1] *NRR´* means morpholine; [2] *NRR´* means *N,N,N´*-trimethylethylendiamine

Individual oligonucleotides were grouped (Tab. 12) by type of modification and the obtained curves were overlayed. First, we compared the melting points of ^{modif}DNA*DNA duplexes with those found for unmodified DNA*DNA duplex.

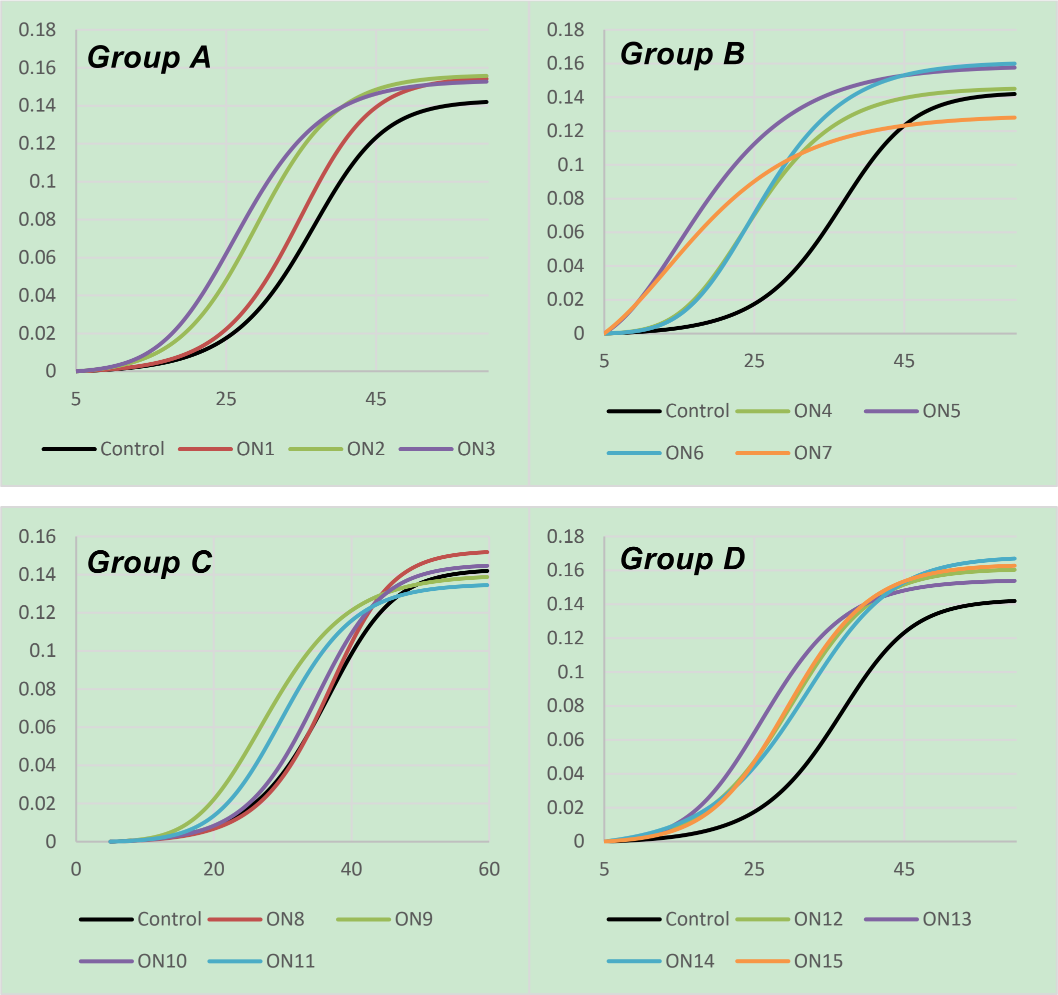

Figure 26. Thermal characteristics (melting curves) of ^{modif}DNA*DNA duplexes compared with unmodified control DNA*DNA duplex: For structure of ^{modif}DNA **ON1-ON15** see Table 12 (Group A-Group D).

The increasing number of odifications in the modified DNA strands **ON1-ON3** decreased melting points of ^{modif}DNA*DNA duplexes (Tab. 12; Fig. 26 *Group A*). Strongly destabilizing effect exhibited also **ON4-ON7** with *3´O-P(S)-CH$_2$-S5´* and *3´O-P(S)-O5´* linkages (Tab. 12; Fig. 26 *Group B*). Interestingly, there is only a little difference in T_m values between natural DNA*DNA duplex and **ON8***DNA

and **ON10***DNA duplexes containing *3´O-P(O)-CH_2-O5´* and *3´O-P(S)-CH_2-O5´* internucleotide linkages, respectively (Tab. 12; Fig. 26 *Group C*).

Very nice results we obtained with ^{modif}DNA*RNA heteroduplexes composed of natural RNA target and **ON8-ON11** with combination of *3´O-P(O)-O5´*, *3´O-P(S)-O5´*, *3´O-P(O)-CH_2-O5´*, and *3´O-P(S)-CH_2-O5´* internucleotide linkages (Tab. 12; Fig. 27 *Group C*). The phosphonate oligonucleotide **ON8** without *P-SH* bond prepared earlier in our lab served as the second reference oligonucleotide, exhibited in heteroduplex much higher T_m value than T_m value of the natural DNA*RNA heteroduplex. Also **ON9-ON11** with various number of P-SH bonds exhibited in heteroduplexes T_m values surpassing T_m value of natural heteroduplex.

Moreover we found that the thermal stability of the **ON9***RNA duplex with three *3´O-P(S)-CH_2-O5´* and five *3´O-P(S)-O-5´* internucleotide linkages surpassed the stability of **ON3***RNA heteroduplex with the eight phosphorothioate *3´O-P(S)-O-5´* internucleotide linkages. The same trend was observed in case of **ON9** and **ON3** with three *3´O-P(S)-CH_2-O5´* and five *3´O-P(S)-O-5´* internucleotide linkages, respectively. It suggested that the *P(-SH)-CH_2* moiety can reduce the destabilizing effect of the present *P-SH* bonds to the extent that three phosphonothioate linkages compensate for the destabilizing effect of eight phosphorothioate *P-SH* bonds. This fact is very encouraging and very useful since oligonucleotides with suitably located phosphonothioate linkages may still exhibit high thermal stability of ^{modif}DNA*RNA heteroduplexes. In case of phosphonoamidates (**ON12** and **ON14**) and phosphoroamidates (**ON13** and **ON15**) we can conclude that the former compounds are more thermally stable in heteroduplex that the latter phosphoroamidates (Tab. 12; Fig. 27 *Group D*).

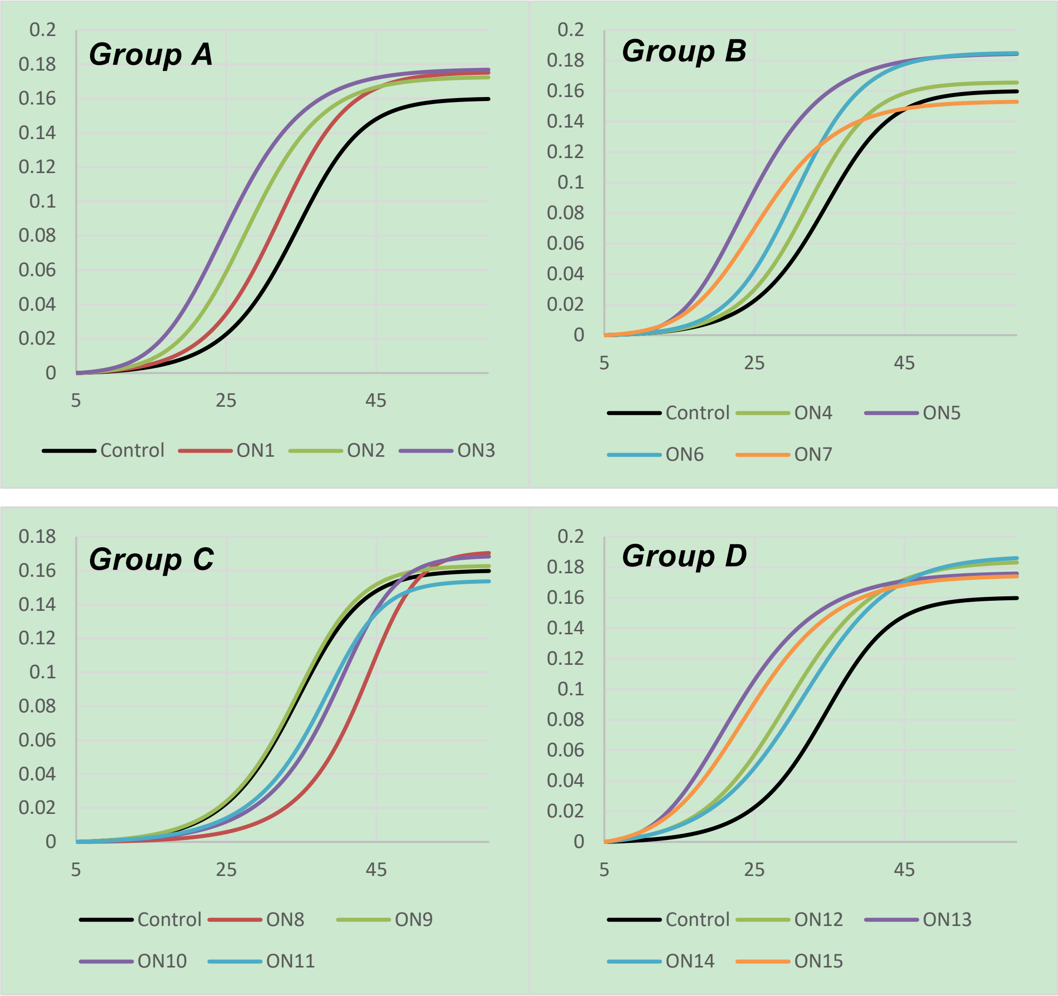

Figure 27. Thermal characteristics (melting curves) of ^{modif}DNA*RNA duplexes compared with unmodified control DNA*RNA duplex: For structures of ^{modif}DNA **ON1-ON15** see Table 12(*Group A-Group D*).

Histograms summarized, in terms of ΔT_m values, the obtained results on measurements of thermal characteristic of prepared ^{modif}DNA*DNA homoduplexes and ^{modif}DNA*RNA heteroduplexes. From the calculated melting points (T_m values), the difference to the control DNA*DNA (Fig. 29) and DNA*RNA (Fig. 29) duplexes was determined (ΔT_m) and further divided by the number of modifications to obtain a change of T_m value *per* one modification (ΔT_m */modif.*) (Fig. 30; Fig. 31). Additionally, differences between T_m values for ^{modif}DNA*DNA and ^{modif}DNA*RNA (Fig. 32) for duplexes indicated the selectivity of the modified DNA strand to DNA or RNA complementary strands (Tab. 13).

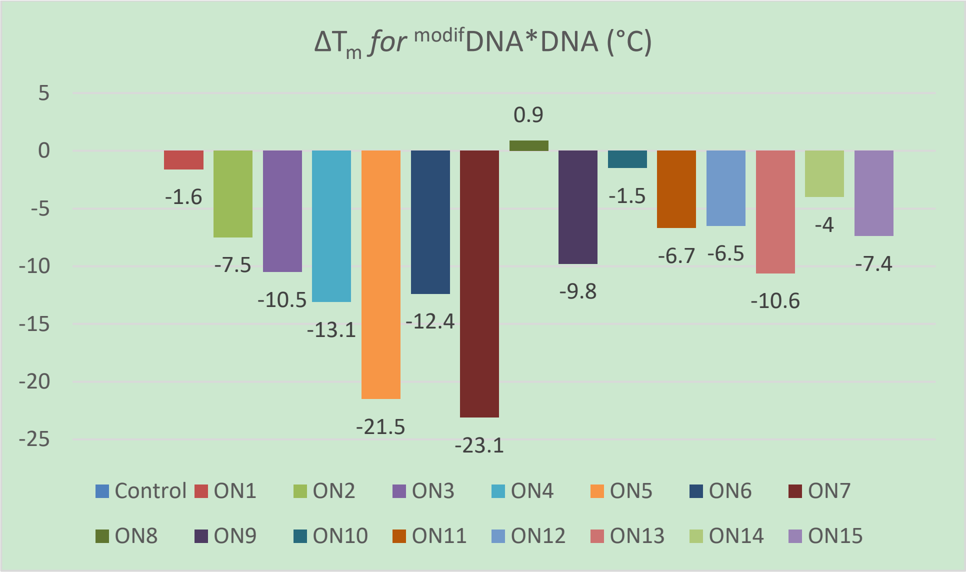

Figure 28. Histogram of ΔT_m values for ^{modif}DNA*DNA; **ON1-ON3** (*Group A*), **ON4-ON7** (*Group B*), **ON8-ON11** (*Group C*), **ON12-ON15** (*Group D*), For structures of ^{modif}DNA **ON1-ON15** see Table 12(*Group A-Group D*).

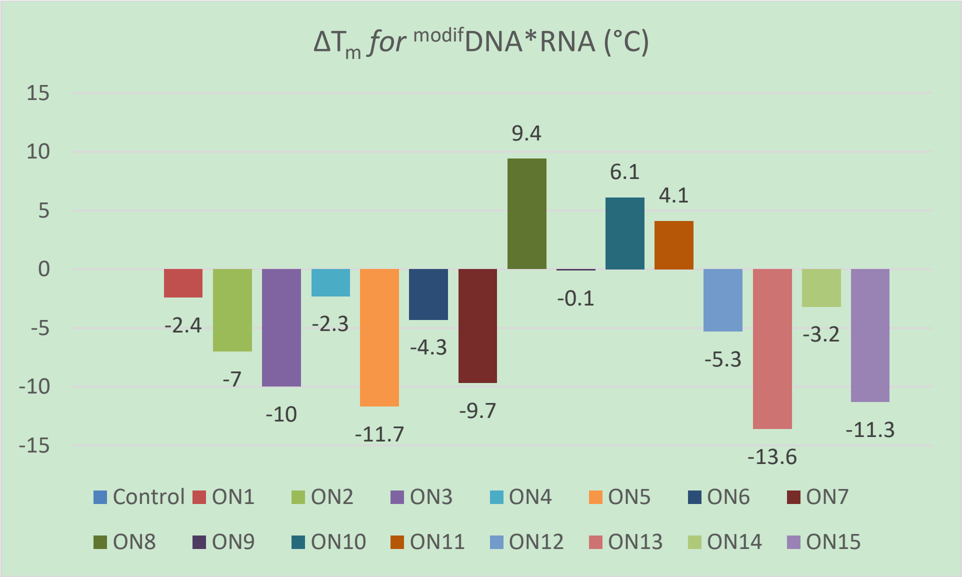

Figure 29. Histogram of ΔT_m values per modification for ^{modif}DNA*RNA; **ON1-ON3** (*Group A*), **ON4-ON7** (*Group B*), **ON8-ON11** (*Group C*), **ON12-ON15** (*Group D*), For structures of ^{modif}DNA **ON1-ON15** see Table 12(*Group A-Group D*).

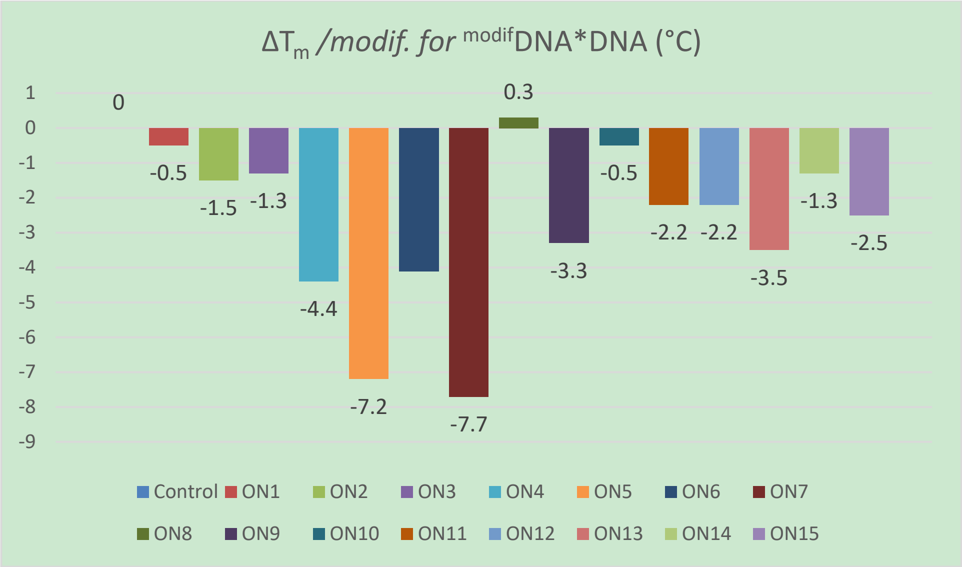

Figure 30. Histogram of ΔT_m values per modification for [modif]DNA*DNA; **ON1-ON3** (*Group A*), **ON4-ON7** (*Group B*), **ON8-ON11** (*Group C*), **ON12-ON15** (*Group D*), For structures of [modif]DNA **ON1-ON15** see Table 12(*Group A-Group D*).

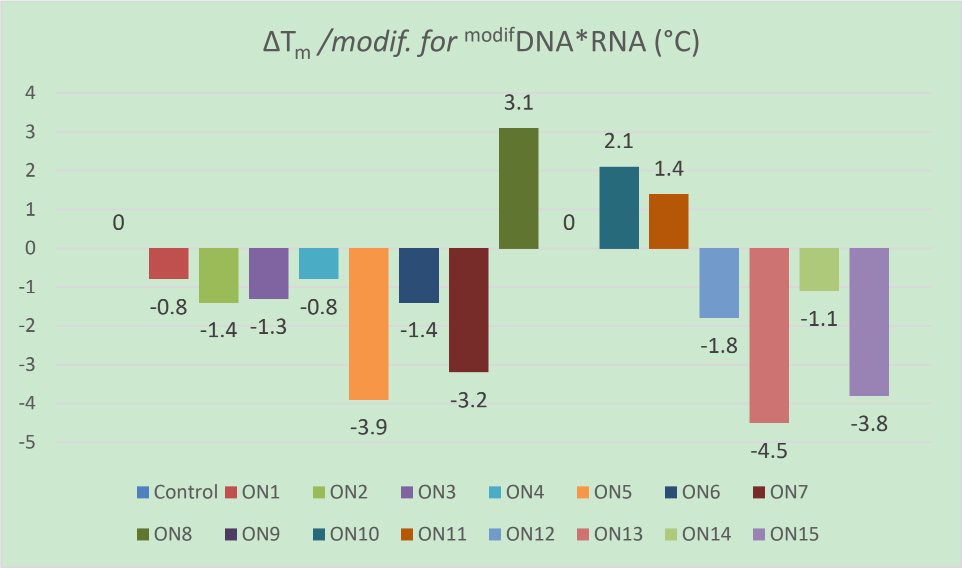

Figure 31. Histogram of ΔT_m values per modification for [modif]DNA*RNA; **ON1-ON3** (*Group A*), **ON4-ON7** (*Group B*), **ON8-ON11** (*Group C*), **ON12-ON15** (*Group D*), For structures of [modif]DNA **ON1-ON15** see Table 12(*Group A-Group D*).

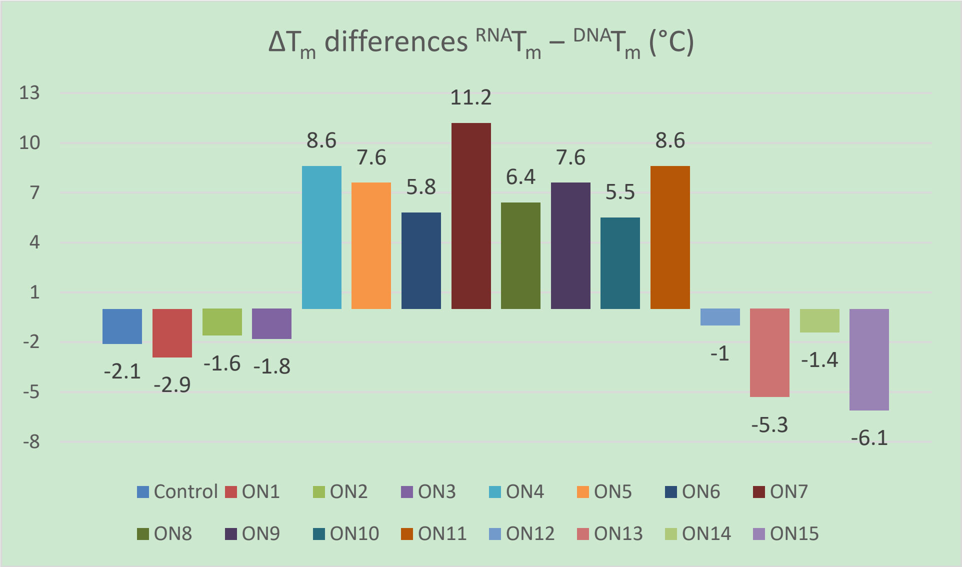

Figure 32. Histogram of ΔT_m differences ($^{RNA}T_m$ – $^{DNA}T_m$) indicating selectivity of the modified oligonucleotide strand to DNA or RNA complementary strands; **ON1-ON3** (*Group A*), **ON4-ON7** (*Group B*), **ON8-ON11** (*Group C*), **ON12-ON15** (*Group D*), For structures of ^{modif}DNA **ON1-ON15** see Table 12(*Group A-Group D*).

Table 13. Summary of duplex thermal stability of modified oligonucleotides compared to their DNA and RNA complements.

Data in °C	*T_m DNA*	*ΔT_m DNA*	*ΔT_m/modif DNA*	*T_m RNA*	*ΔT_m RNA*	*ΔT_m/modif RNA*	*$^{RNA}T_m$ - $^{DNA}T_m$*
Control	36.6	0	0	34.5	0	0	-2.1
ON1	35	-1.6	-0.5	32	-2.4	-0.8	-2.9
ON2	29	-7.5	-1.5	27	-7	-1.4	-1.6
ON3	26	-10	-1.3	24	-10	-1.3	-1.8
ON4	24	-13	-4.4	32	-2.3	-0.8	8.6
ON5	15	-22	-7.2	23	-12	-3.9	7.6
ON6	24	-12.4	-4.1	30	-4.3	-1.4	5.8
ON7	14	-23	-7.7	25	-9.7	-3.2	11
ON8	38	0.9	0.3	44	9.4	3.1	6
ON9	27	-9.8	-3.3	34	-0.1	0	7.6
ON10	35	-1.5	-0.5	41	6.1	2.1	5.5
ON11	30	-6.7	-2.2	39	4.1	1.4	8.6
ON12	30	-6.5	-2.2	29	-5.3	-1.8	-1
ON13	26	-11	-3.5	21	-14	-4.5	-5.3
ON14	33	-4.0	-1.3	31	-3.2	-1.1	-1.4
ON15	29	-7.4	-2.5	23	-11.3	-3.8	-6.1

3. CHARACTERIZATION OF OLIGONUCLEOTIDES

The 5´→3´ trityl-off synthesis of oligonucleotide was performed by phosphoramidite method (standard conditions recommended by the supplier) and *H*-phosphonate method on GeneSyn and MOS synthesizers (both product of IOCB, Prague) using 3´-*O*-dimethoxytritylnucleoside-5´-hemisuccinate-modified LCAA-CPG (or TentaGel) solid support in a 0.5 µmol scale.

Phosphoramidite chemistry: All monomers, oxidation and sulfurization agents were used according to the supplier's recommendations (Glen Research).

H-phosphonate chemistry: Pyridine-acetonitrile (50:50) solutions of 0.1M monomers (110 µL) and 0.3M 2-chloro-5,5-dimethyl-1,3,2-dioxaphosphorinane-2-oxide (110 µL) were used for each coupling step (10 min). Currently used capping with isopropyl-(*H*)-phosphonate was omitted because of inconclusive effect on the chromatographic profile of deprotected oligonucleotides (data not shown). The oxidation of the oligonucleotide chain differed in each example. In case of methyl ester, the support was dried *in vacuo*, treated with freshly prepared thiophenol-Et_3N-DMF mixture (23:32:45; v/v) for 4 h, rinsed with dry acetonitrile, dried under vacuum (in case of cyanoethyl ester was thiophenol treatment omitted), and treated with gaseous NH_3 (0.7 MPa) at rt overnight. The deprotected and released oligonucleotide was eluted from CPG with acetonitrile/water (1 mL; 1:1, *v/v*) and analyzed on a DNAPac PA100 column (4 × 250 mm; Dionex) at a flow rate of 1 mL/min using different gradients. The purification of oligonucleotide was performed on a DNAPac PA100 column (9 × 250 mm at a flow rate of 3 mL/min). Pure oligonucleotide was desalted on a Luna C18 column (5 µm; 10 × 50 mm; Phenomenex) at a flow rate of 5 mL/min using a linear gradient of acetonitrile (0 →50%, 20 min) in 0.1 M aq. triethylammonium hydrogencarbonate buffer (pH 8); effluent containing oligonucleotide was evaporated to dryness on CentriVap and co-evaporated with MeOH (3 x 1 mL). The identity was confirmed with MALDI-TOF.

Mobile phases:

Mobile phase **I**: A, 20 mM NaOAc and 20 mM NaCl in MeCN-H_2O (10:90); B, 20 mM NaOAc and 1.5 M NaCl in MeCN-H_2O (10:90).

Mobile phase **II**: A, 25 mM TRIS/HCl and 10 mM $NaClO_4$ in MeCN-H_2O (10:90); B, 25 mM NaOAc and 0.5 M $NaClO_4$ in MeCN-H_2O (10:90).

Mobile phase **III**: A, 0.02 M NaOH and 20 mM NaCl in MeCN-H_2O (10:90); B, 20 mM NaOH and 1.5 M NaCl in MeCN-H_2O (10:90).

dT $_{10}$ ***1 – IEC HPLC profile and MALDI TOF***

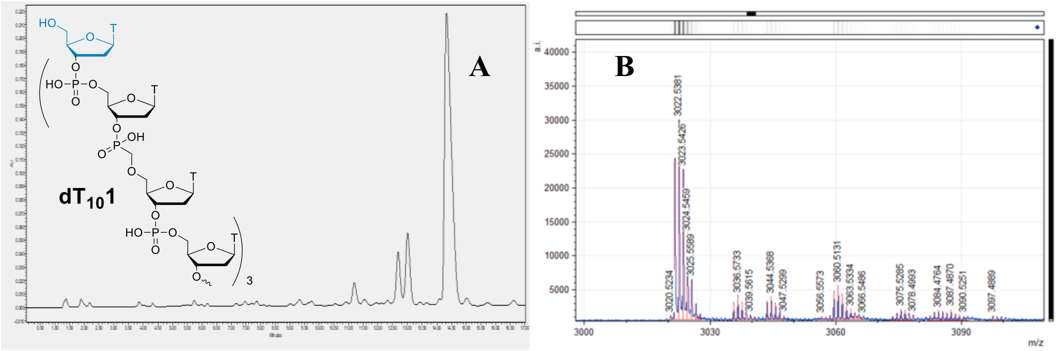

	Method	*HPLC Purity*	*Amount**	*M - Calc.*	*MALDI-TOF*
	**Synthetic Protocol A**	70%	330.9 nmol		
dT $_{10}$ ***1***	**Synthetic Protocol B**	74%	345.3 nmol	3020.5516	3020.5733
	**Synthetic Protocol C**	64%	310.7 nmol		

Figure 33. (**A**) IEC HPLC of crude **dT**$_{10}$**1**, mobile phase **I** (GRAD 0% B to 15% B in 20min IEC C5); (**B**) MALDI TOF of **dT**$_{10}$**1**; (**C**) Table of results, *amount was calculated from the peak area and extinction coefficient by nearest neighbor method [22, 23] (Sequer app.).

dT$_{10}$2 – IEC HPLC profile and MALDI TOF

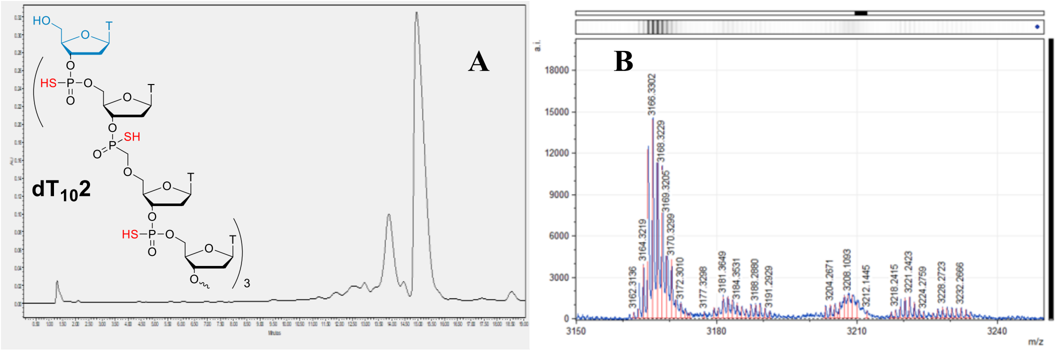

	Method	HPLC Purity	Amount*	M - Calc.	MALDI-TOF
dT$_{10}$2	**Synthetic Protocol A**	59%	248.1 nmol	3164.3460	3164.3219
	**Synthetic Protocol B**	77%	341.0 nmol		

Figure 34. (**A**) IEC HPLC of crude **dT$_{10}$2**, mobile phase **II** (GRAD 0% B to 50% B in 20min IEC C6); (**B**) MALDI TOF of **dT$_{10}$2**; (**C**) Table of results, * amount was calculated from the peak area and extinction coefficient by nearest neighbor method [22, 23] (Sequer app.).

dT$_{10}$3 – IEC HPLC profile and MALDI TOF

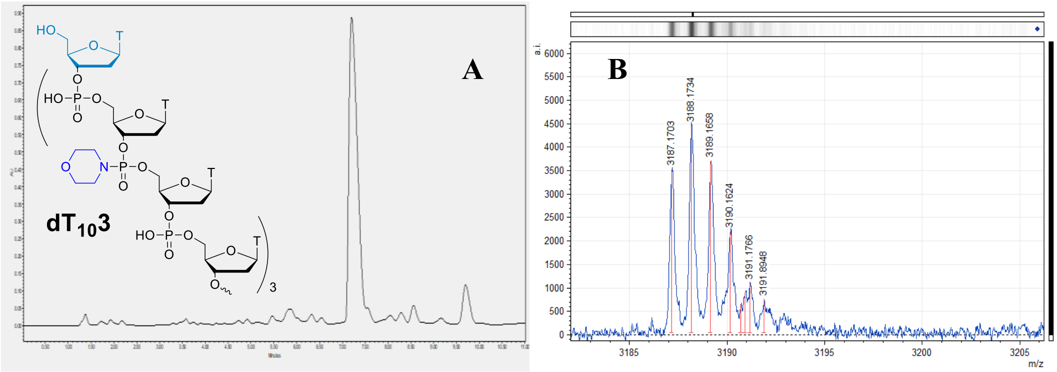

	Method	HPLC Purity	Amount*	M - Calc.	MALDI-TOF
dT$_{10}$3	**Synthetic Protocol B**	36%	159.6 nmol	3187.3049	3187.1703
	**Synthetic Protocol C**	88%	478.4 nmol		

Figure 35. (**A**) IEC HPLC of crude **dT$_{10}$3**, mobile phase **I** (GRAD 0% B 15% B in 20min IEC C5); (**B**) MALDI TOF of **dT$_{10}$3**; (**C**) Table of results, * amount was calculated from the peak area and extinction coefficient by nearest neighbor method [22, 23] (Sequer app.).

$dT_{10}4$ – IEC HPLC profile and MALDI TOF

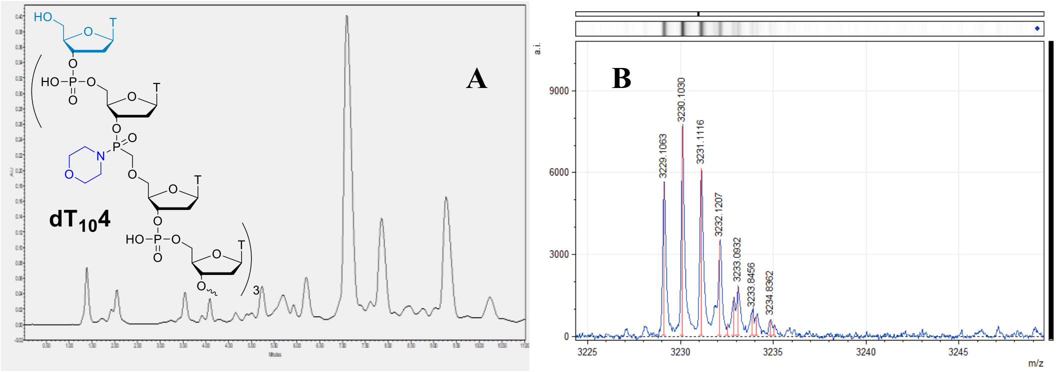

	Method	HPLC Purity	Amount*	M - Calc.	MALDI-TOF
$dT_{10}4$	**Synthetic Protocol B**	42%	135.4 nmol	3229.3859	3229.1063
	**Synthetic Protocol C**	70%	184.1 nmol		

Figure 36. (**A**) IEC HPLC of crude **$dT_{10}4$**, mobile phase **I** (GRAD 0% B to 15% B in 20min IEC C5); (**B**) MALDI TOF of **$dT_{10}4$**; (**C**) Table of results, * amount was calculated from the peak area and extinction coefficient by nearest neighbor method [22, 23] (Sequer app.).

$dT_{10}5$ – IEC HPLC profile and MALDI TOF

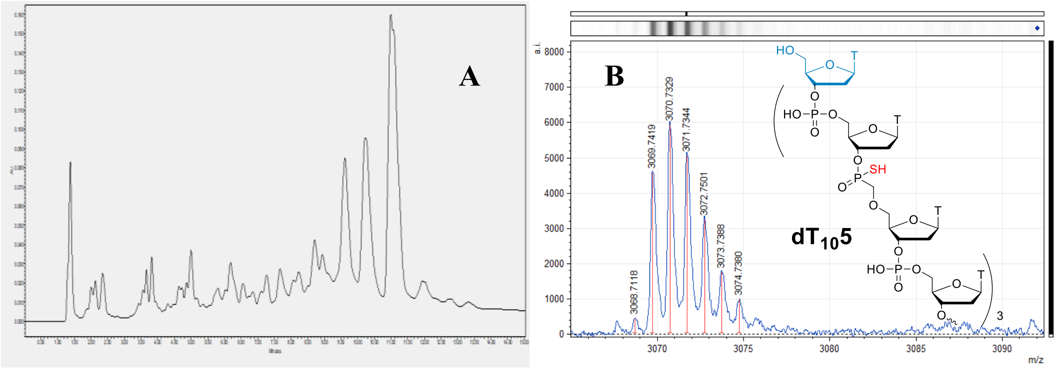

	Method	HPLC Purity	Amount*	M - Calc.	MALDI-TOF
$dT_{10}5$	**Synthetic Protocol C**	30%	116.3 nmol	3070.2479	3070.7329

Figure 37. (**A**) IEC HPLC of crude **$dT_{10}5$**, mobile phase **I** (GRAD 0% B to 25% B in 20min IEC C5); (**B**) MALDI TOF of **$dT_{10}5$**; (**C**) Table of results, * amount was calculated from the peak area and extinction coefficient by nearest neighbor method [22, 23] (Sequer app.).

dT$_{10}$6 – IEC HPLC profile and MALDI TOF

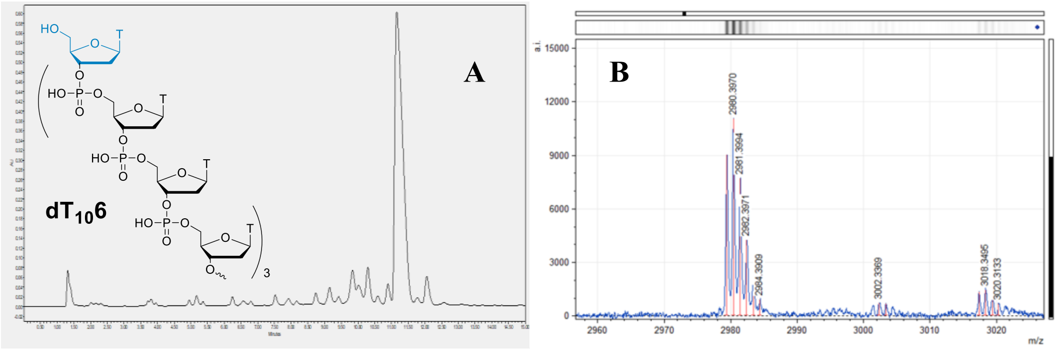

	Method	HPLC Purity	Amount*	M - Calc.	MALDI-TOF
dT$_{10}$6	**Synthetic Protocol C**	75%	398.0 nmol	2979.9839	2980.3970

Figure 38. (**A**) IEC HPLC of crude **dT$_{10}$6**, mobile phase **I** (GRAD 0%B to 25%B in 20min IEC C5); (**B**) MALDI TOF of **dT$_{10}$6**; (**C**) Table of results, * amount was calculated from the peak area and extinction coefficient by nearest neighbor method [22, 23] (Sequer app.).

dT$_{10}$7 – IEC HPLC profile and MALDI TOF

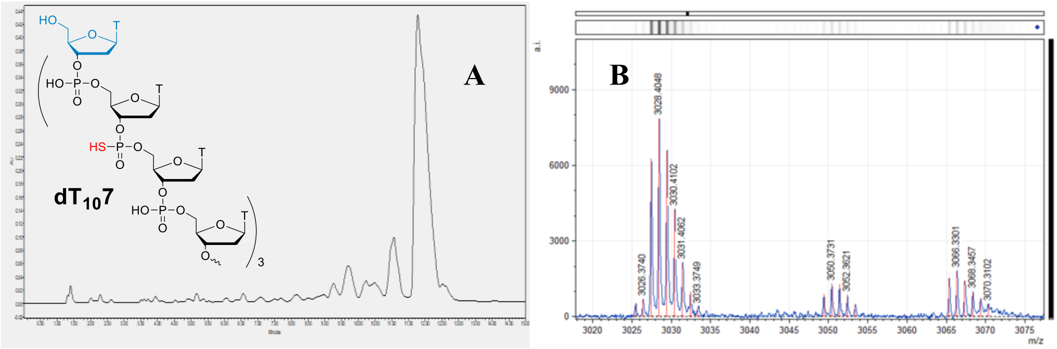

	Method	HPLC Purity	Amount*	M - Calc.	MALDI-TOF
dT$_{10}$7	**Synthetic Protocol C**	77%	471.0 nmol	3028.1696	3028.4048

Figure 39. (**A**) IEC HPLC of crude **dT$_{10}$7**, mobile phase **I** (GRAD 0%B to 20%B in 20min IEC C5); (**B**) MALDI TOF of **dT$_{10}$7**; (**C**) Table of results, * amount was calculated from the peak area and extinction coefficient by nearest neighbor method [22, 23] (Sequer app.).

ON1 – IEC HPLC profiles

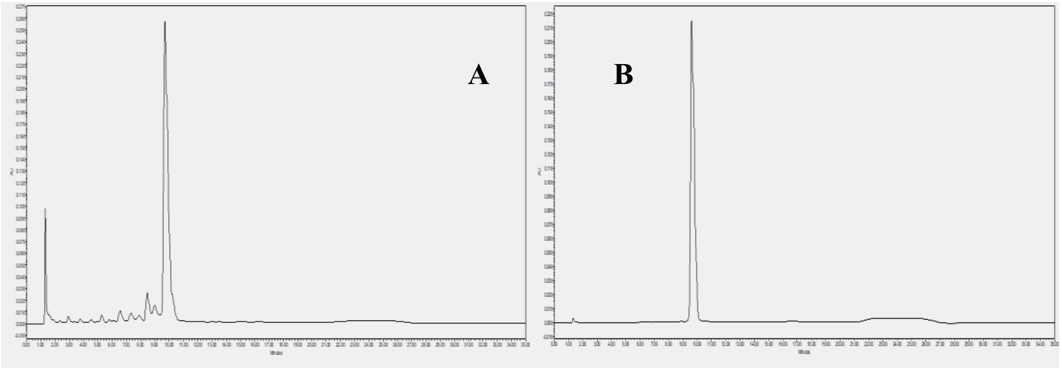

	Method	*HPLC Purity**	*Isolated yield*	*M - Calc.*	*MALDI-TOF*
ON1	**Synthetic Protocol B**	86%	202.6 nmol	2729.4322	2729.5331

Figure 40. (**A**) IEC HPLC of crude **ON1**, mobile phase **II** (GRAD 5% B to 30% B in 20min IEC C6); (**B**) IEC HPLC of purified product **ON1**, MALDI TOF in appendix Fig. A1; (**C**) Table of results, * amount was calculated from the peak area and extinction coefficient by nearest neighbor method [22, 23] (Sequer app.).

ON2 – IEC HPLC profiles

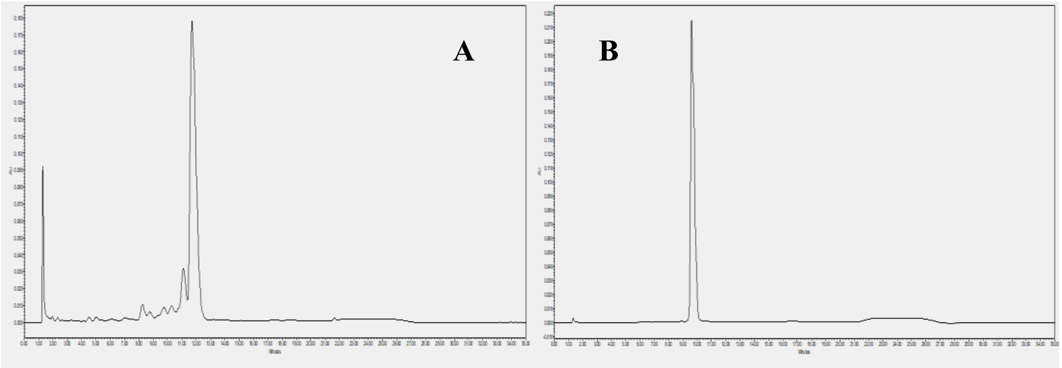

	Method	*HPLC Purity*	*Isolated yield*	*M - Calc.*	*MALDI-TOF*
ON2	**Synthetic Protocol B**	84%	189.5 nmol	2761.3865	2761.1589

Figure 41. (**A**) IEC HPLC of crude **ON2**, mobile phase **II** (GRAD 5% B 30% B in 20min IEC C6); (**B**) IEC HPLC of purified product **ON2**, MALDI TOF in appendix Fig. A2; (**C**) Table of results, * amount was calculated from the peak area and extinction coefficient by nearest neighbor method [22, 23] (Sequer app.).

ON3 – IEC HPLC profiles

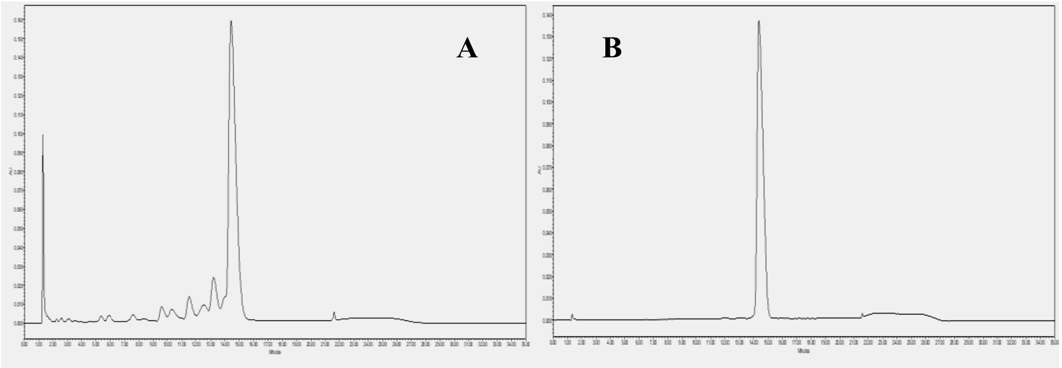

	Method	*HPLC Purity**	*Isolated yield*	*M - Calc.*	*MALDI-TOF*
ON3	**Synthetic Protocol B**	81%	201.0 nmol	2809.3180	2809.3292

Figure 42. (**A**) IEC HPLC of crude **ON3**, mobile phase **II** (GRAD 5% B to 35% B in 20min IEC C6); (**B**) IEC HPLC of purified product **ON3**, MALDI TOF in appendix Fig. A3; (**C**) Table of results, * amount was calculated from the peak area and extinction coefficient by nearest neighbor method [22, 23] (Sequer app.).

ON4 – IEC HPLC profiles

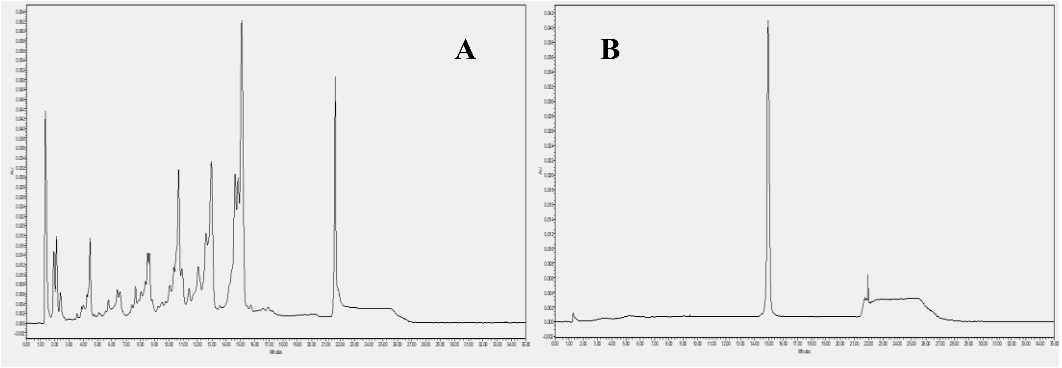

	Method	*HPLC Purity**	*Isolated yield*	*M_{Cals}*	*MALDI-TOF*
ON4	**Synthetic Protocol B**	21%	30.0 nmol	2771.4792	2771.3983

Figure 43. (**A**) IEC HPLC of crude **ON4**, mobile phase **I** (GRAD 0% B to 15% B in 20min IEC C5); (**B**) IEC HPLC of purified product **ON4**, MALDI TOF in appendix Fig. A4; (**C**) Table of results, * amount was calculated from the peak area and extinction coefficient by nearest neighbor method [22, 23] (Sequer app.).

ON5 – IEC HPLC profiles

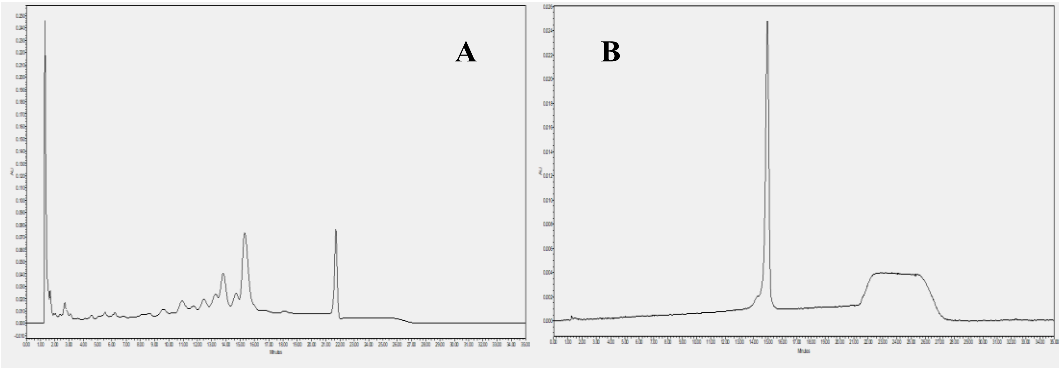

	Method	*HPLC Purity**	*Isolated yield*	*M - Calc.*	*MALDI-TOF*
ON5	**Synthetic Protocol B**	56%	67.7 nmol	2899.2964	2899.2376

Figure 44. (**A**) IEC HPLC of crude **ON5**, mobile phase **II** (GRAD 5% B to 35% B in 20min IEC C6); (**B**) IEC HPLC of purified product **ON5**, MALDI TOF in appendix Fig. A5; (**C**) Table of results, * amount was calculated from the peak area and extinction coefficient by nearest neighbor method [22, 23] (Sequer app.).

ON6 – IEC HPLC profiles

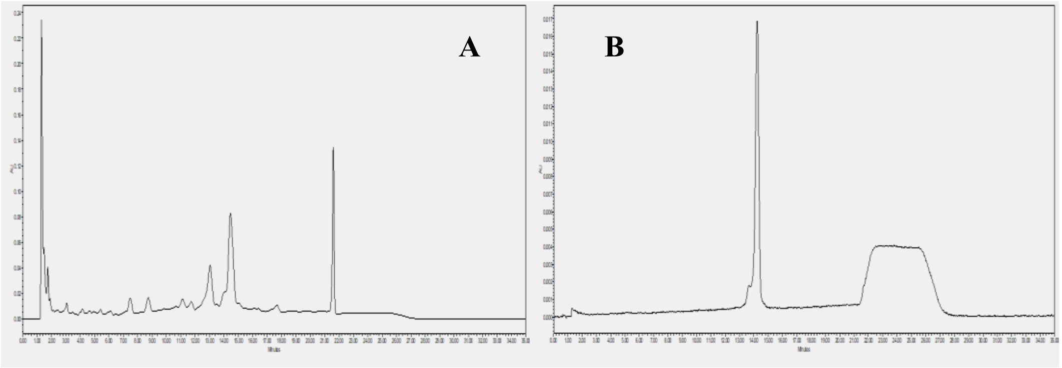

	Method	*HPLC Purity**	*Isolated yield*	*M - Calc.*	*MALDI-TOF*
ON6	**Synthetic Protocol B**	51%	57.7 nmol	2819.4106	2819.2887

Figure 45. (**A**) IEC HPLC of crude **ON6**, mobile phase **II** (GRAD 5% B to 20% B in 20min IEC C6); (**B**) IEC HPLC of purified product **ON6**, MALDI TOF in appendix Fig. A6; (**C**) Table of results, * amount was calculated from the peak area and extinction coefficient by nearest neighbor method [22, 23] (Sequer app.).

ON7 – IEC HPLC profiles

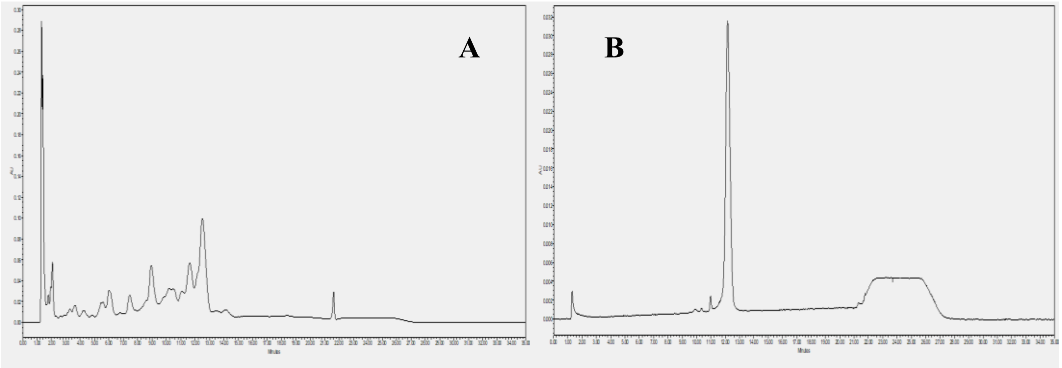

	Method	*HPLC Purity**	*Isolated yield*	*M - Calc.*	*MALDI-TOF*
ON7	**Synthetic Protocol B**	43%	57.7 nmol	2851.3650	2851.3559

Figure 46. (**A**) IEC HPLC of crude **ON7**, mobile phase **II** (GRAD 5% B to 30% B in 20min IEC C6); (**B**) IEC HPLC of purified product **ON7**, MALDI TOF in appendix Fig. A7; (**C**) Table of results, * amount was calculated from the peak area and extinction coefficient by nearest neighbor method [22, 23] (Sequer app.).

ON8 – IEC HPLC profiles

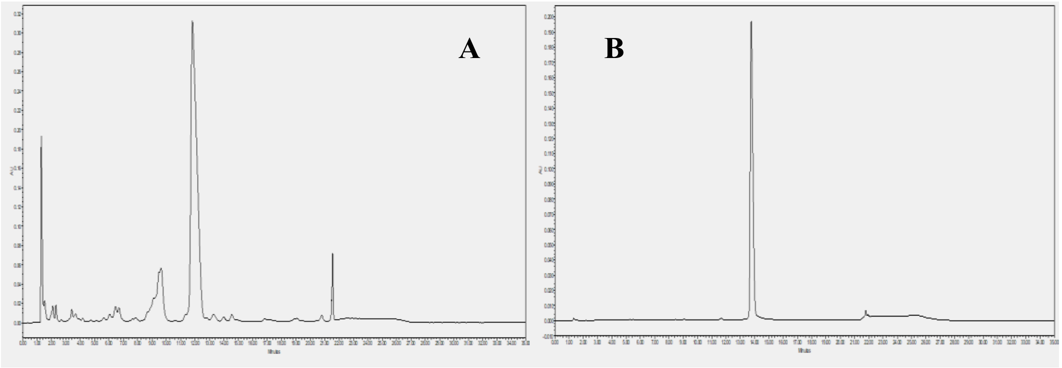

	Method	*HPLC Purity**	*Isolated yield*	*M - Calc.*	*MALDI-TOF*
ON8	**Synthetic Protocol B**	76%	341.2 nmol	2723.5477	2723.7076

Figure 47. (**A**) IEC HPLC of crude **ON8**, mobile phase **II** (GRAD 5% B to 15% B in 20min IEC C6); (**B**) IEC HPLC of purified product **ON8**, MALDI TOF in appendix Fig. A8; (**C**) Table of results, * amount was calculated from the peak area and extinction coefficient by nearest neighbor method [22, 23] (Sequer app.).

ON9 – IEC HPLC profiles

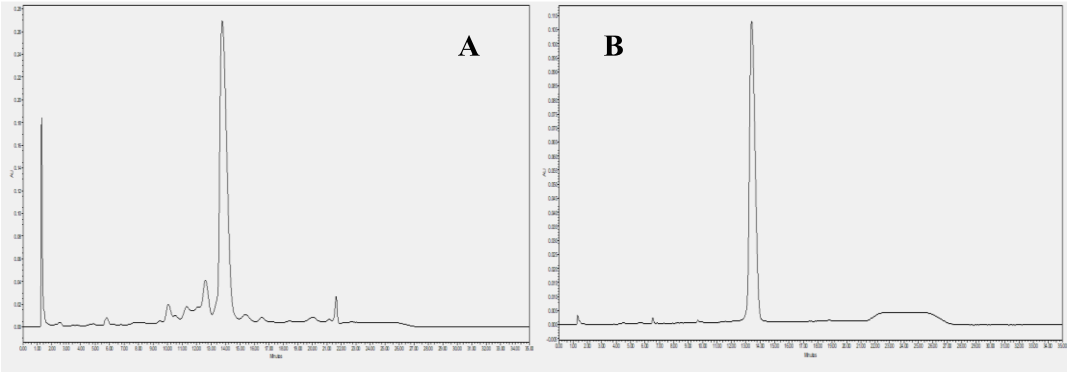

	Method	HPLC Purity*	Isolated yield	M - Calc.	MALDI-TOF
ON9	**Synthetic Protocol B**	84%	330.8 nmol	2851.3650	2851.2513

Figure 48. (**A**) IEC HPLC of crude **ON9**, mobile phase **II** (GRAD 5% B to 35% B in 20min IEC C6); (**B**) IEC HPLC of purified product **ON9**, MALDI TOF in appendix Fig. A9; (**C**) Table of results, * amount was calculated from the peak area and extinction coefficient by nearest neighbor method [22, 23] (Sequer app.).

ON10 – IEC HPLC profiles

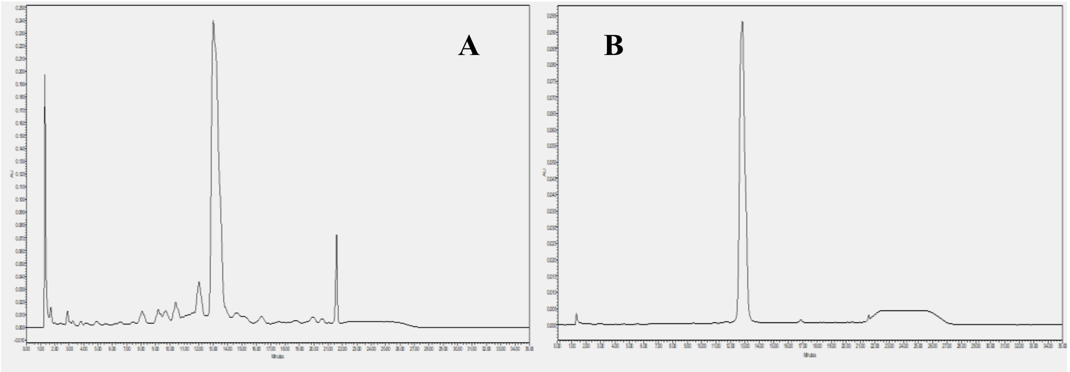

	Method	HPLC Purity*	Isolated yield	M - Calc.	MALDI-TOF
ON10	**Synthetic Protocol B**	86%	307.2 nmol	2771.4791	2771.3636

Figure 49. (**A**) IEC HPLC of crude **ON10**, mobile phase **II** (GRAD 5% B to 20% B in 20min IEC C6); (**B**) IEC HPLC of purified product **ON10**, MALDI TOF in appendix Fig. A10; (**C**) Table of results, * amount was calculated from the peak area and extinction coefficient by nearest neighbor method [22, 23] (Sequer app.).

ON11 – IEC HPLC profiles

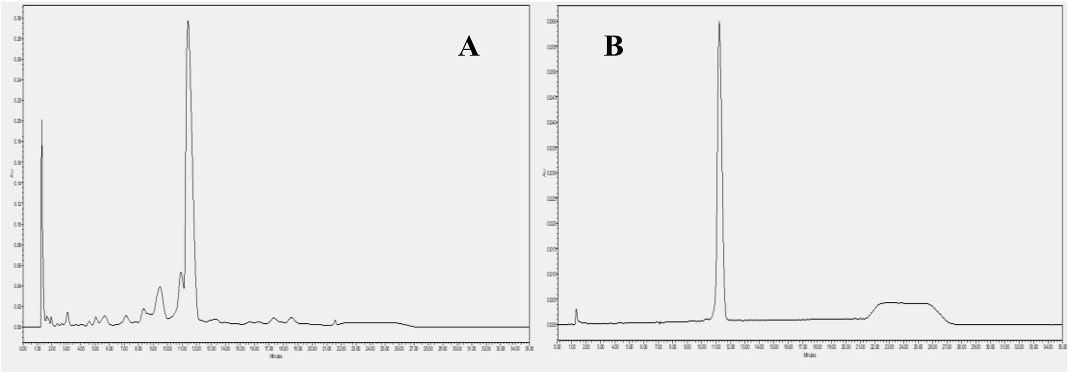

	Method	HPLC Purity*	Isolated yield	M - Calc.	MALDI-TOF
ON11	**Synthetic Protocol B**	86%	328.1 nmol	2803.4335	2803.5659

Figure 50. (**A**) IEC HPLC of crude **ON11**, mobile phase **II** (GRAD 5% B to 30% B in 20min IEC C6); (**B**) IEC HPLC of purified product **ON11**, MALDI TOF in appendix Fig. A11; (**C**) Table of results, * amount was calculated from the peak area and extinction coefficient by nearest neighbor method [22, 23] (Sequer app.).

ON12 – IEC HPLC profiles

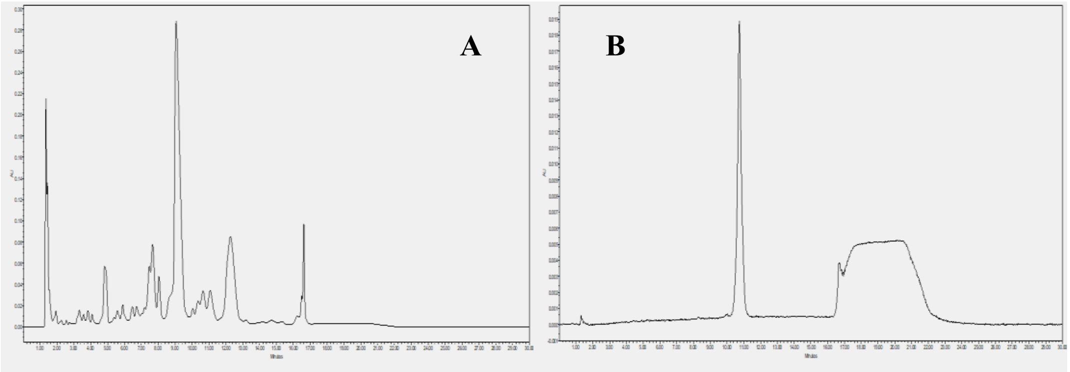

	Method	HPLC Purity*	Isolated yield	M - Calc.	MALDI-TOF
ON12	**Synthetic Protocol C**	59%	147.1 nmol	2930.7213	2930.6797

Figure 51. (**A**) IEC HPLC of crude **ON12**, mobile phase **III** (GRAD 5% B to 20% B in 20min IEC C6); (**B**) IEC HPLC of purified product **ON12**, MALDI TOF in appendix Fig. A12; (**C**) Table of results, * amount was calculated from the peak area and extinction coefficient by nearest neighbor method [22, 23] (Sequer app.).

ON13 – IEC HPLC profiles

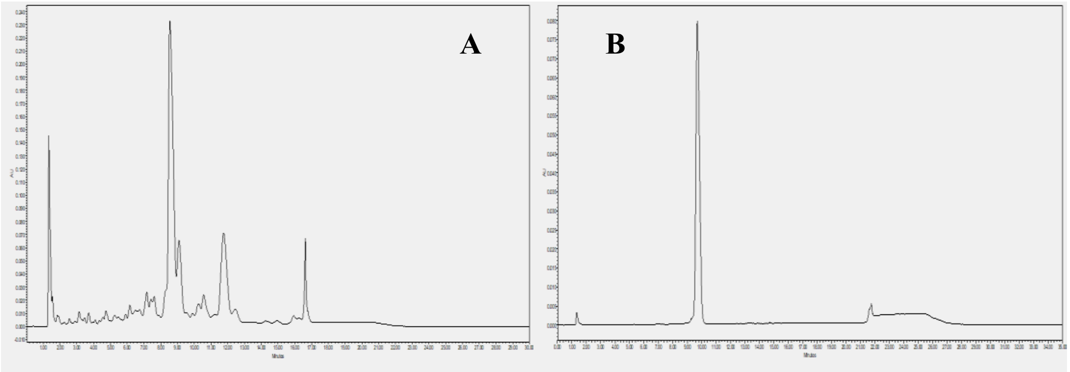

	Method	HPLC Purity*	Isolated yield	M - Calc.	MALDI-TOF
ON13	**Synthetic Protocol C**	66%	110.2 nmol	2975.8631	2975.7741

Figure 52. (**A**) IEC HPLC of crude **ON13**, mobile phase **III** (GRAD 5% B to 20% B in 20min IEC C6); (**B**) IEC HPLC of purified product **ON13**, MALDI TOF in appendix Fig. A13; (**C**) Table of results, * amount was calculated from the peak area and extinction coefficient by nearest neighbor method [22, 23] (Sequer app.).

ON14 – IEC HPLC profiles

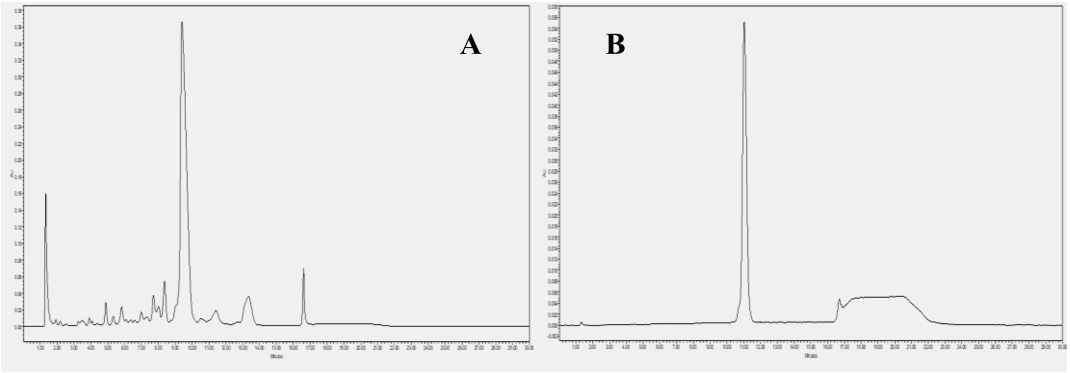

	Method	HPLC Purity*	Isolated yield	M - Calc.	MALDI-TOF
ON14	**Synthetic Protocol C**	78%	297.5 nmol	2888.6743	2888.8365

Figure 53. (**A**) IEC HPLC of crude **ON14**, mobile phase **III** (GRAD 5% B to 35% B in 20min IEC C6); (**B**) IEC HPLC of purified product **ON14**, MALDI TOF in appendix Fig. A14; (**C**) Table of results, * amount was calculated from the peak area and extinction coefficient by nearest neighbor method [22, 23] (Sequer app.).

ON15 – IEC HPLC profiles

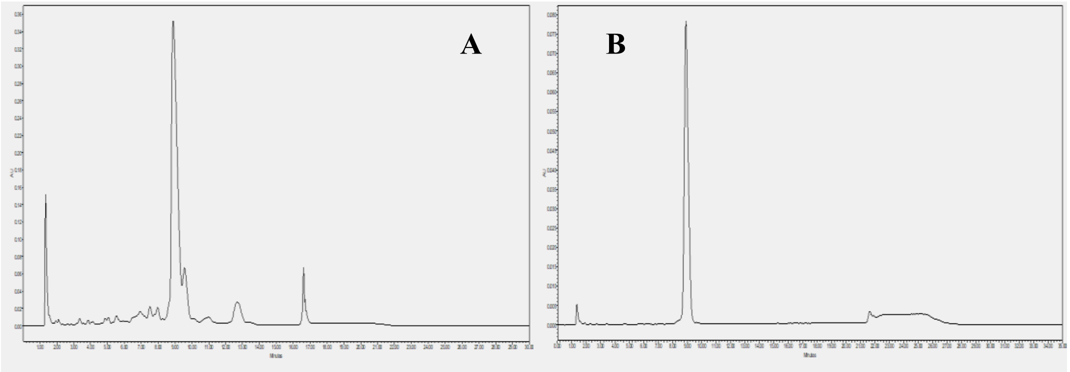

	Method	*HPLC Purity**	*Isolated yield*	*M - Calc.*	*MALDI-TOF*
ON15	**Synthetic Protocol C**	79%	233.9 nmol	2933.8162	2933.7591

Figure 54. (**A**) IEC HPLC of crude **ON15**, mobile phase **III** (GRAD 5% B to 20% B in 20min IEC C6); (**B**) IEC HPLC of purified product **ON15**, MALDI TOF in appendix Fig. A15; (**C**) Table of results, * amount was calculated from the peak area and extinction coefficient by nearest neighbor method [22, 23] (Sequer app.).

4. REFERENCES

1. Kostov, O.; Páv, O.; Buděšínský, M.; Liboska, R.; Šimák, O.; Petrová, M.; Novák, P.; Rosenberg, I., 4-Toluenesulfonyloxymethyl-(H)-phosphinate: A Reagent for the Introduction of O- and S-Methyl-(H)-phosphinate Moieties. *Organic Letters* **2016,** *18* (11), 2704-2707.
2. Kostov, O.; Páv, O.; Rosenberg, I., Nucleoside-O-Methyl-(H)-Phosphinates: Novel Monomers for the Synthesis of Methylphosphonate Oligonucleotides Using H-Phosphonate Chemistry. *Current Protocols in Nucleic Acid Chemistry* **2017,** *70* (1), 4.76.1-4.76.22.
3. Hargreaves, J.; Leproust, E. M. Methods and compositions useful in the preparation of oligonucleotides. 2008.
4. Paul, C. H.; Royappa, A. T., Acid Binding and Detritylation During Oligonucleotide Synthesis. *Nucleic Acids Research* **1996,** *24* (15), 3048-3052.
5. Glen Research UNICAP PHOSPHORAMIDITE, AN ALTERNATIVE TO ACETIC ANHYDRIDE CAPPING. http://www.glenresearch.com/GlenReports/GR17-13.html (accessed 10.9.2018).
6. Scozzari, A., Process for oligonucleotide synthesis. Google Patents: 2000.
7. Stawinski, J.; Stromberg, R., Di- and oligonucleotide synthesis using H-phosphonate chemistry. *Methods in molecular biology (Clifton, N.J.)* **2005,** *288,* 81-100.
8. Garegg, P. J.; Regberg, T.; Stawinski, J.; Stromberg, R., Nucleoside phosphonates: part 7. Studies on the oxidation of nucleoside phosphonate esters. *Journal of the Chemical Society, Perkin Transactions 1* **1987,** (0), 1269-1273.
9. Cullis, P. M.; Lee, M., The mechanism of iodine-water oxidation of H-phosphonate diesters. *Journal of the Chemical Society, Chemical Communications* **1992,** (17), 1207-1208.
10. Wada, T.; Mochizuki, A.; Sato, Y.; Sekine, M., A convenient method for phosphorylation involving a facile oxidation of H-Phosphonate monoesters via bis(trimethylsilyl) phosphites. *Tetrahedron Letters* **1998,** *39* (39), 7123-7126.
11. Maier, M. A.; Guzaev, A. P.; Manoharan, M., Synthesis of Chimeric Oligonucleotides Containing Phosphodiester, Phosphorothioate, and Phosphoramidate Linkages. *Organic Letters* **2000,** *2* (13), 1819-1822.
12. Bartoszewicz, A.; Kalek, M.; Stawinski, J., The Case for the Intermediacy of Monomeric Metaphosphate Analogues during Oxidation of H-Phosphonothioate, H-Phosphonodithioate, and H-Phosphonoselenoate Monoesters: Mechanistic and Synthetic Studies. *The Journal of Organic Chemistry* **2008,** *73* (13), 5029-5038.
13. Le Corre, S. S.; Berchel, M.; Couthon-Gourvès, H.; Haelters, J.-P.; Jaffrès, P.-A., Atherton–Todd reaction: mechanism, scope and applications. *Beilstein Journal of Organic Chemistry* **2014,** *10*, 1166-1196.

14. Xiong, B.; Zhou, Y.; Zhao, C.; Goto, M.; Yin, S.-F.; Han, L.-B., Systematic study for the stereochemistry of the Atherton–Todd reaction. *Tetrahedron* **2013,** *69* (45), 9373-9380.
15. Letsinger, R. L.; Singman, C. N.; Histand, G.; Salunkhe, M., Cationic oligonucleotides. *Journal of the American Chemical Society* **1988,** *110* (13), 4470-4471.
16. Jung, P. M.; Histand, G.; Letsinger, R. L., Hybridization of Alternating Cationic/Anionic Oligonucleotides to RNA Segments. *Nucleosides and Nucleotides* **1994,** *13* (6-7), 1597-1605.
17. Wallin, R.; Kalek, M.; Bartoszewicz, A.; Thelin, M.; Stawinski, J., On the Sulfurization of H-Phosphonate Diesters and Phosphite Triesters Using Elemental Sulfur. *Phosphorus, Sulfur, and Silicon and the Related Elements* **2009,** *184* (4), 908-916.
18. Brill, W. K. D., Thioalkylation of nucleoside-H-phosphonates and its application to solid phase synthesis of oligonucleotides. *Tetrahedron Letters* **1995,** *36* (5), 703-706.
19. Reese, C. B.; Yan, H., Solution phase synthesis of ISIS 2922 (Vitravene) by the modified H-phosphonate approach. *Journal of the Chemical Society, Perkin Transactions 1* **2002,** (23), 2619-2633.
20. Shu, C.; Li, X.; Li, P., The mechanism of double-stranded DNA sensing through the cGAS-STING pathway. *Cytokine Growth Factor Rev.* **2014,** *25* (6), 641-648.
21. Li, Y.; Wilson, H. L.; Kiss-Toth, E., Regulating STING in health and disease. *Journal of Inflammation (London, England)* **2017,** *14*, 11.
22. Warshaw, M. M.; Tinoco, I., Optical properties of sixteen dinucleoside phosphates. *Journal of Molecular Biology* **1966,** *20* (1), 29-38.
23. Cavaluzzi, M. J.; Borer, P. N., Revised UV extinction coefficients for nucleoside-5′-monophosphates and unpaired DNA and RNA. *Nucleic Acids Research* **2004,** *32* (1), e13-e13.

5. APPENDIX

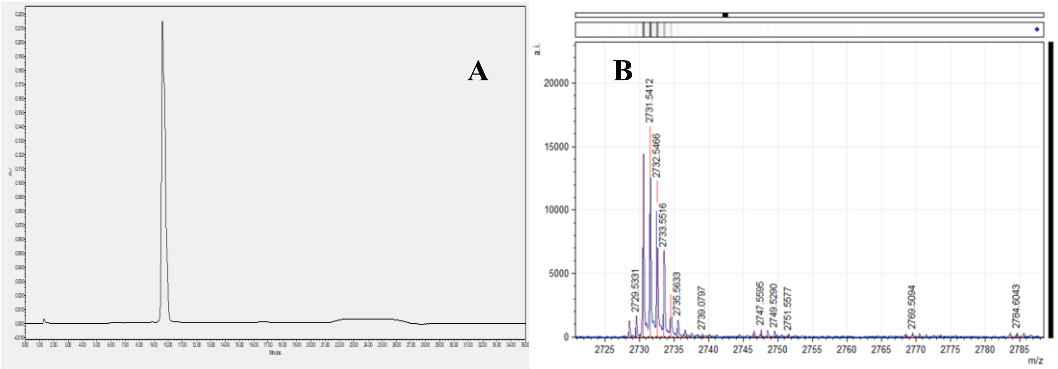

Figure A1. (**A**) IEC HPLC of purified product **ON1**; (**B**) MALDI TOF of **ON1**.

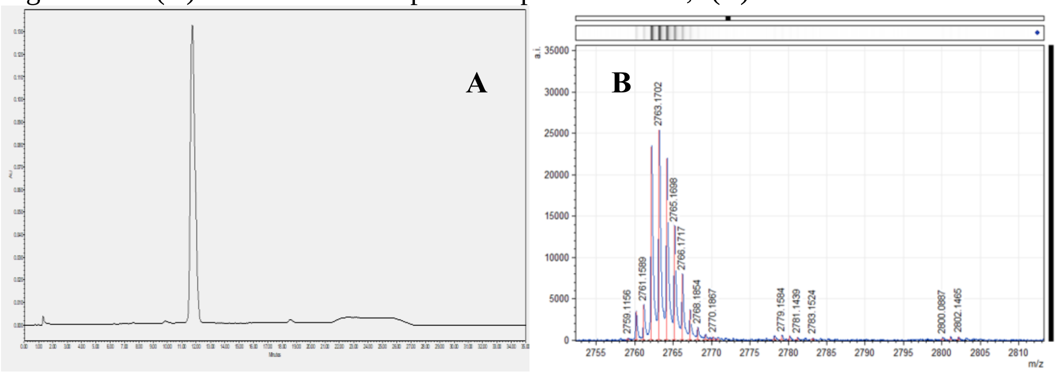

Figure A2. (**A**) IEC HPLC of purified product **ON2**; (**B**) MALDI TOF of **ON2**.

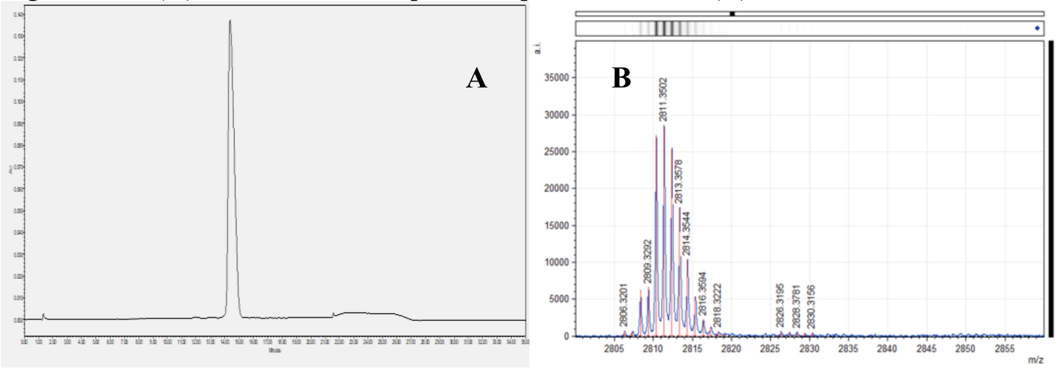

Figure A3. (**A**) IEC HPLC of purified product **ON3**; (**B**) MALDI TOF of **ON3**.

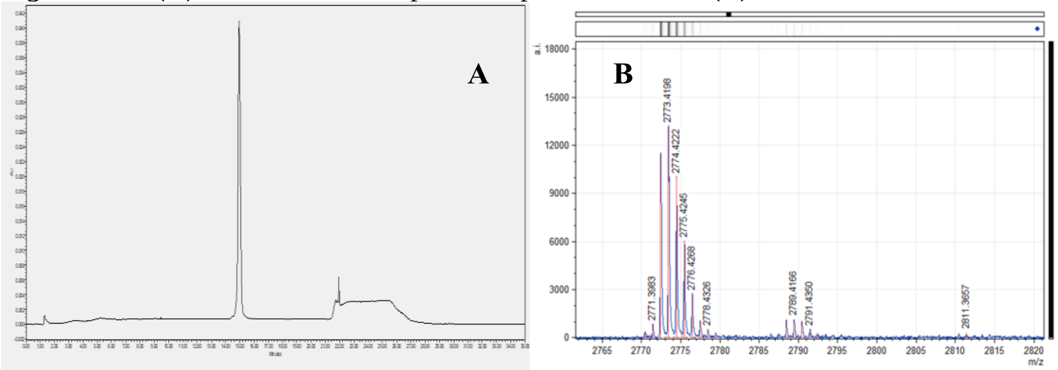

Figure A4. (**A**) IEC HPLC of purified product **ON4**; (**B**) MALDI TOF of **ON4**.

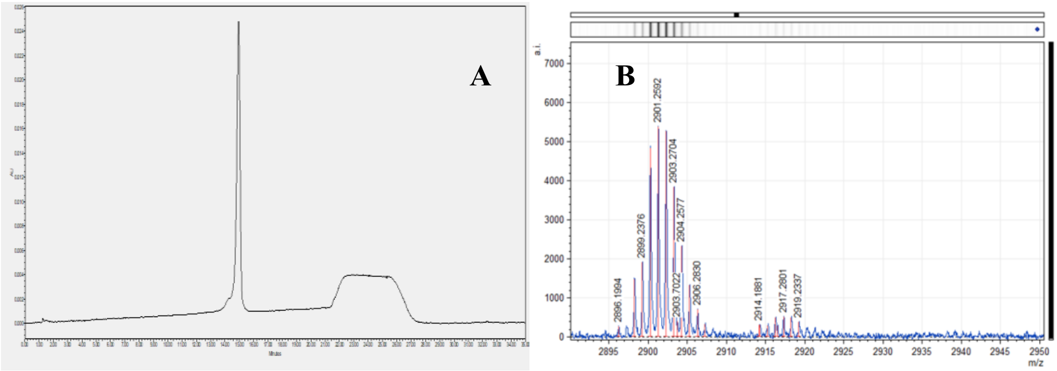

Figure A5. (**A**) IEC HPLC of purified product **ON5**; (**B**) MALDI TOF of **ON5**.

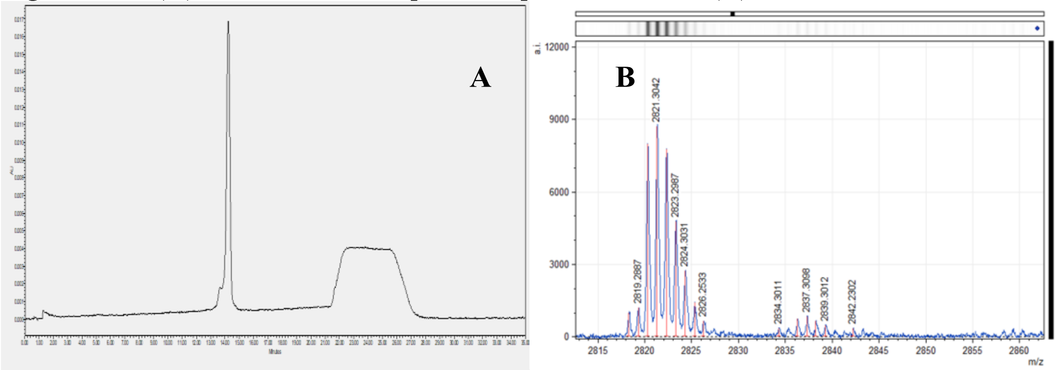

Figure A6. (**A**) IEC HPLC of purified product **ON6**; (**B**) MALDI TOF of **ON6**.

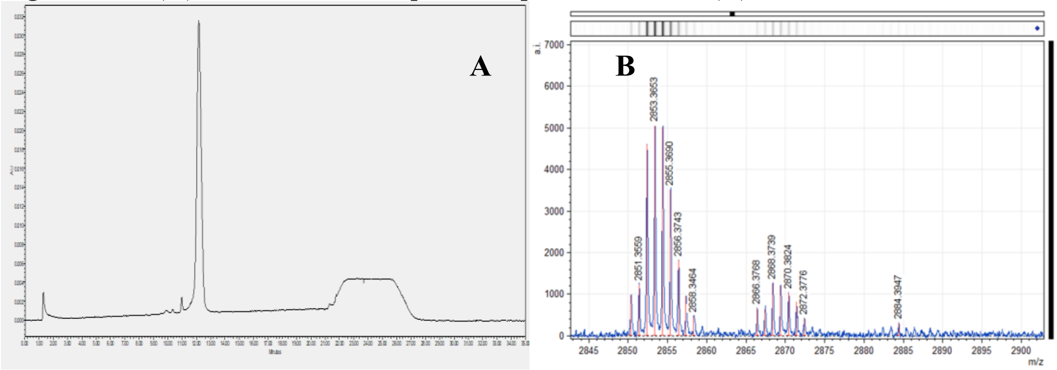

Figure A7. (**A**) IEC HPLC of purified product **ON7**; (**B**) MALDI TOF of **ON7**.

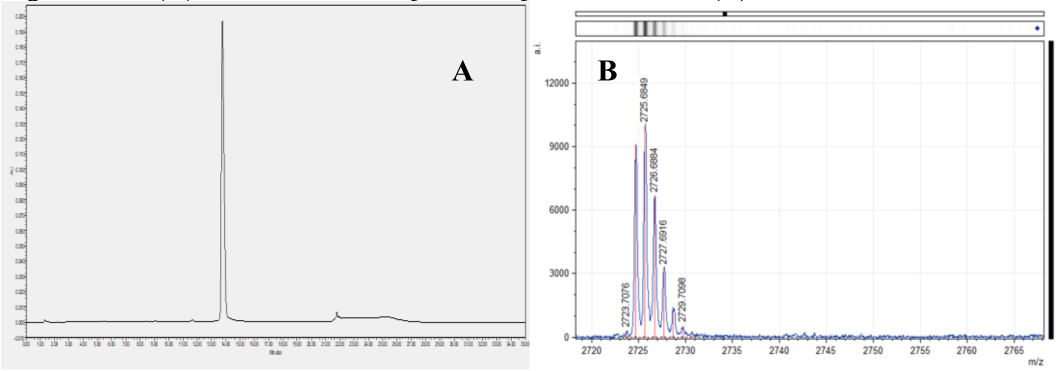

Figure A8. (**A**) IEC HPLC of purified product **ON8**; (**B**) MALDI TOF of **ON8**.

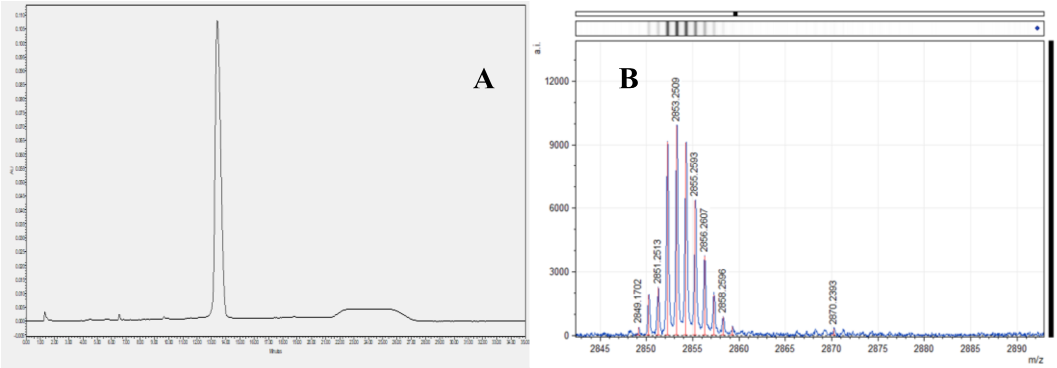

Figure A9. (**A**) IEC HPLC of purified product **ON9**; (**B**) MALDI TOF of **ON9**.

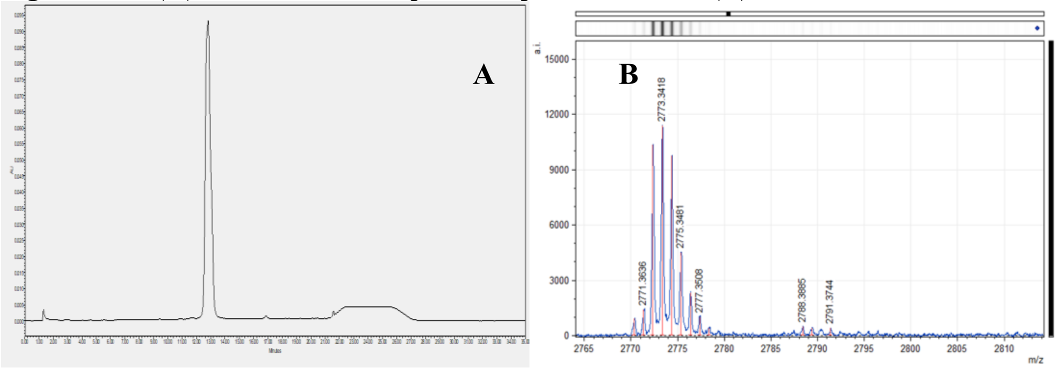

Figure A10. (**A**) IEC HPLC of purified product **ON10**; (**B**) MALDI TOF of **ON10**.

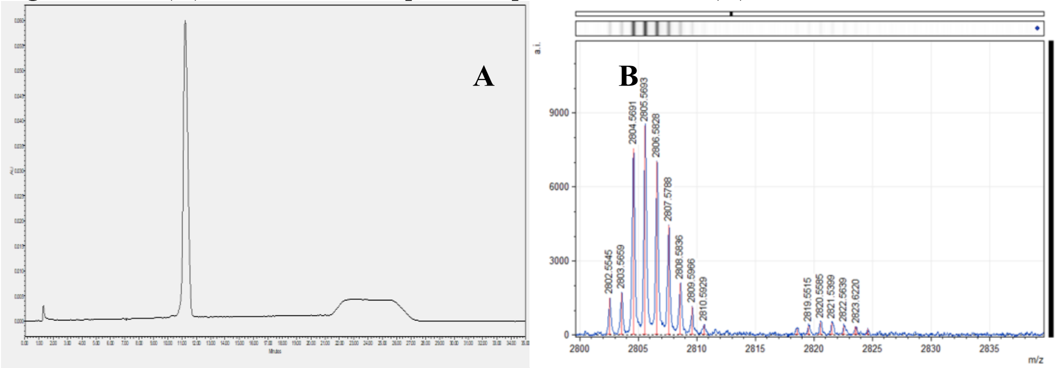

Figure A11. (**A**) IEC HPLC of purified product **ON11**; (**B**) MALDI TOF of **ON11**.

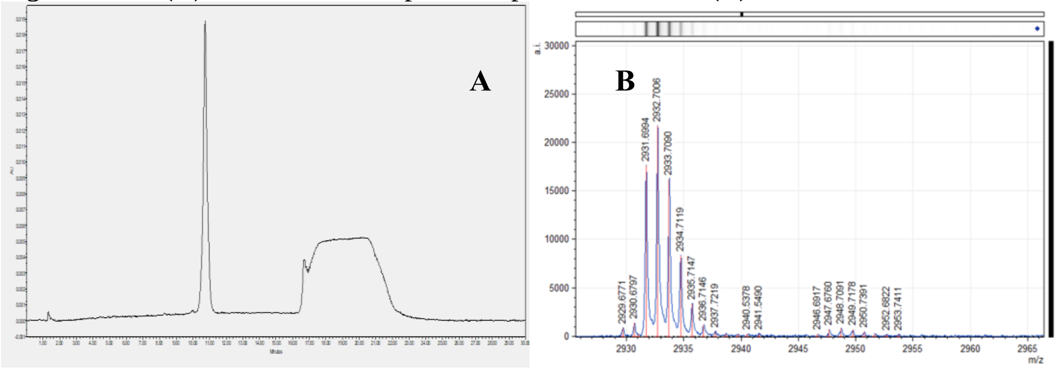

Figure A12. (**A**) IEC HPLC of purified product **ON12**; (**B**) MALDI TOF of **ON12**.

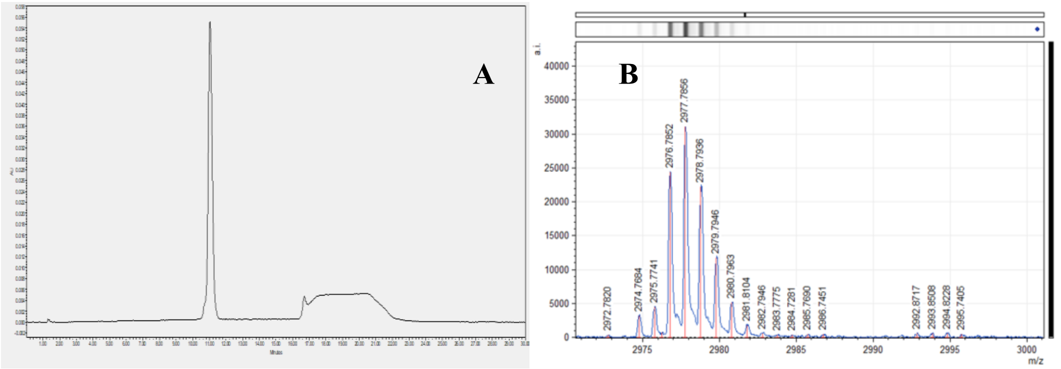

Figure A13. (**A**) IEC HPLC of purified product **ON13**; (**B**) MALDI TOF of **ON13**.

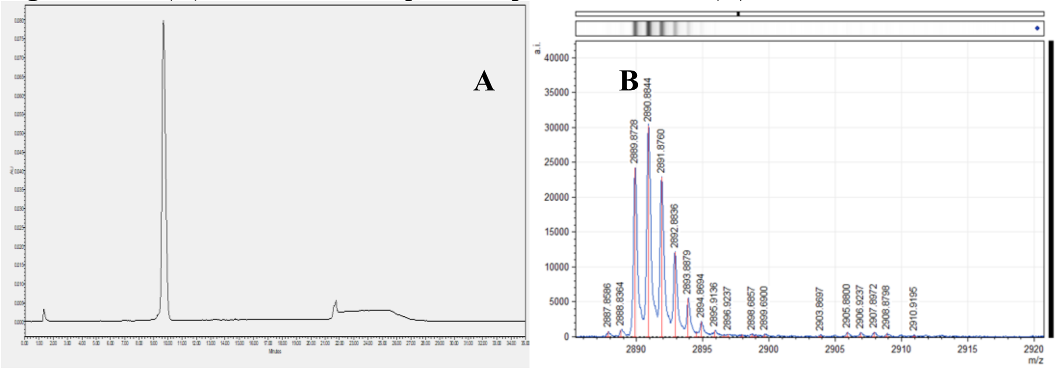

Figure A14. (**A**) IEC HPLC of purified product **ON14**; (**B**) MALDI TOF of **ON14**.

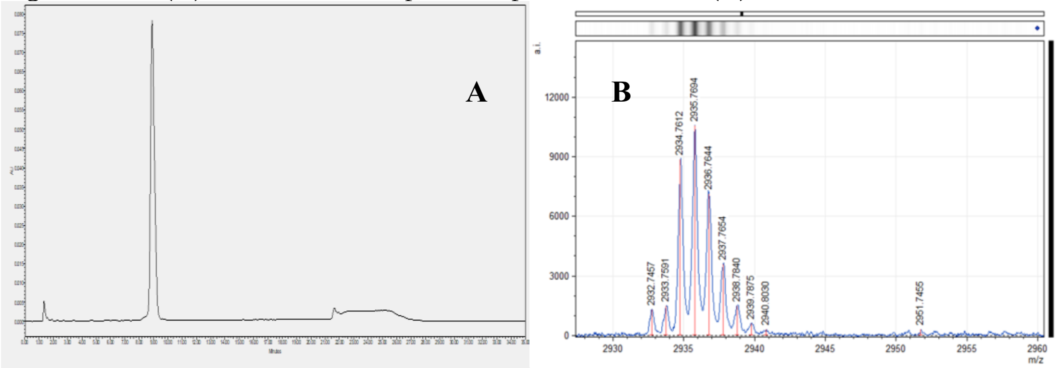

Figure A15. (**A**) IEC HPLC of purified product **ON15**; (**B**) MALDI TOF of **ON15**.
